# A Novel Immune-Related lncRNA-Based Model for Survival Prediction in Clear Cell Renal Cell Carcinoma

**DOI:** 10.1155/2021/9921466

**Published:** 2021-06-28

**Authors:** Zedan Zhang, Yanlin Tang, Yanjun Liu, Hongkai Zhuang, Enyu Lin, Lu Xie, Xiaoqiang Feng, Kaiwen Tian, Jiayi Zeng, Jiumin Liu, Yuming Yu

**Affiliations:** ^1^Department of Urology, Guangdong Provincial People's Hospital, Guangdong Academy of Medical Sciences, Guangzhou, China; ^2^Shantou University Medical College, Shantou, China; ^3^Department of Immunology, School of Basic Medical Science, Southern Medical University, Guangzhou, China; ^4^Department of General Surgery, Guangdong Provincial People's Hospital, Guangdong Academy of Medical Sciences, Guangzhou, China; ^5^Center of Stem Cell and Regenerative Medicine, Gaozhou People's Hospital, Maoming, Guangdong, China

## Abstract

**Background:**

Clear cell renal cell carcinoma (ccRCC) is the most common subtype of kidney cancer whose incidence and mortality rate are increasing. Identifying immune-related lncRNAs and constructing a model would probably provide new insights into biomarkers and immunotherapy for ccRCC and aid in the prognosis prediction.

**Methods:**

The transcription profile and clinical information were obtained from The Cancer Genome Atlas (TCGA). Immune-related gene sets and transcription factor genes were downloaded from GSEA website and Cistrome database, respectively. Tumor samples were divided into the training set and the testing set. Immune-related differentially expressed lncRNAs (IDElncRNAs) were identified from the whole set. Univariate Cox regression, LASSO, and stepwise multivariate Cox regression were performed to screen out ideal prognostic IDElncRNAs (PIDElncRNAs) from the training set and develop a multi-lncRNA signature.

**Results:**

Consequently, AC012236.1, AC078778.1, AC078950.1, AC087318.1, and AC092535.4 were screened to be significantly related to the prognosis of ccRCC patients, which were used to establish the five-lncRNA signature. Its wide diagnostic capacity was revealed in different subgroups of clinical parameters. Then AJCC-stage, Fuhrman-grade, pharmaceutical, age, and risk score regarded as independent prognostic factors were integrated to construct a nomogram, whose good performance in predicting 3-, 5-, and 7-year overall survival of ccRCC patients was revealed by time-dependent ROC curves and verified by the testing sets and ICGC dataset. The calibration plots showed great agreement of the nomogram between predicted and observed outcomes. Functional enrichment analysis showed the signature and each lncRNA were mainly enriched in pathways associated with regulation of immune response. Several kinds of tumor-infiltrating immune cells like regulatory T cells, T follicular helper cells, CD8^+^ T cells, resting mast cells, and naïve B cells were significantly correlated with the signature.

**Conclusion:**

Therefore, we constructed a five-lncRNA model integrating clinical parameters to help predict the prognosis of ccRCC patients. The five immune-related lncRNAs could potentially be therapeutic targets for immunotherapy in ccRCC in the future.

## 1. Introduction

Clear cell renal cell carcinoma (ccRCC) accounting for more than 75% of all kidney cancers has increasing incidence and mortality rate in the worldwide [1] and tends to be diagnosed in advanced stage [2]. However, the Tumor-Node-Metastasis (TNM) staging system, the widely used measure to estimate ccRCC outcomes, is revealed to be insufficient in its predictive value [3]. This would be probably as the result of the heterogeneity of the tumor itself and the complicacy of the pathogenesis [4]. Prognostic models combining TNM stage with additional values, including pathological factors, clinical factors, and molecular markers, have aroused lots of interest and have been approved to possess better prognostic value, which results in demand of identifying ideal and novel epigenetic factors [5].

lncRNAs (long noncoding RNAs) are a type of RNA with length exceeding 200 nucleotides, which do not have the protein-coding capacity [6]. Normally, they are involved in the regulation of gene transcription [7], posttranscriptional regulation [8], and epigenetic regulation [9]. Recently, mounting evidences have suggested that lncRNAs play critical roles in oncogenesis and tumor prognosis [10], some of which called immune-related lncRNAs fulfill their carcinogenic effect through affecting immune response, such as influencing immune activation, tumor-infiltrating immune cells, immune cells' development, differentiation, and migration, and killing cancer cells [11–13]. With the contribution of advanced genome-sequencing technologies, it has been revealed to be associated with multiple levels of gene expression in human cancers [14]. Tumor-infiltrating immune cells, as a tumor prognostic value being investigated recently, were revealed to be associated with prognosis of ccRCC patients [15]. Immunotherapy has been regarded as a promising therapeutic strategy to enhance the outcome of survival in ccRCC patients [16, 17]. However, the sensitive for immunotherapy is not the same for all patients, and the full potential of immunotherapy has not been reached [18, 19]. It needs more acquirement to assess the host antitumor immune response and explore the possible mechanism behind that. Therefore, identifying ideal immune-related lncRNAs as biomarkers for prognosis prediction of ccRCC patients and providing clues to develop individual immunological treatment strategies for improving the outcome of ccRCC patients are crucial and bright.

In our study, a model comprised of five immune-related prognostic lncRNAs and clinical parameters was constructed to predict outcomes of ccRCC patients. High-throughput sequencing data of ccRCC from The Cancer Genome Atlas (TCGA) were analyzed to identify immune-related differentially expressed lncRNAs. Then, the prognostic value of lncRNAs for ccRCC was explored by means of univariate Cox proportional hazards regression analysis, least absolute shrinkage and selection operator (LASSO) method, and multivariate Cox proportional hazards regression analysis after classifying the continuous expression of lncRNAs into categorical values. Next, the risk score was calculated by multiplying the categorical values of the expression of five genes and Cox coefficients, which was used to construct a nomogram together with clinical parameters. Thereafter, we utilized time-dependent receiver operating characteristic (tROC) curve analysis and calibration plot to assess the nomogram. The internal and external validations were performed to enhance conclusive force of prediction. Finally, functional enrichment analysis and coexpressed tumor-infiltrating immune cells were explored to further enrich the immune-related characteristics of the ideal lncRNAs. A regulatory relationship network comprised of coexpressed TF genes and the ideal lncRNAs was constructed to provide the global view of possible transcriptional interactions and reveal the possible regulating mechanism of the lncRNA expression.

## 2. Materials and Methods

### 2.1. Dataset Acquisition and Processing

National Cancer Institute and National Human Genome Research Institute cooperatively constructed a public database called The Cancer Genome Atlas (TCGA) containing genomic, epigenomic, transcriptomic, and proteomic data of 33 cancer types. The transcriptome profiling data including raw counts of lncRNA- and mRNA-sequencing data and corresponding clinical information of KIRC patients were obtained from TCGA dataset (https://portal.gdc.cancer.gov/) in October 2019, in which the methods of acquisition are in line with the guidelines and policies. Relevant samples were excluded according to the following criteria: (1) without clinical information or insufficient information of AJCC-stage, Fuhrman-grade, age, and pharmaceutical; (2) survival time of patients less than 30 days; (3) duplicate samples. Finally, 512 tumor samples with clinicopathological data and 72 normal samples were used for subsequent analysis. The cohort of 512 tumor samples (the whole set) was then randomly divided into two sets by a ratio of 6 to 4, the training set (*n* = 309) and the testing set (*n* = 203) using “sample” function of the R software. The related raw counts of RNA-sequencing data and clinical information were acquired from ICGC (International Cancer Genome Consortium, https://icgc.org/), which is a platform for collaboration of worldwide genomic research across 50 cancer types. After screened with the same excluding criteria as TCGA, the cohort consists of 91 tumor samples (*n* = 91) with survival time information and was used for external validation. The details of clinical characteristics were shown in Table S1 (Supplementary material 1).

The 42 gene sets related to immune response were selected from C5:biological process in Molecular Signatures Database (MSigDB) V7.0 of Gene Set Enrichment Analysis (GSEA, https://www.gsea-msigdb.org/gsea/msigdb/collections.jsp), which covered 2593 immune-related genes (Supplementary material 2 Table S2) and were used to screen immune-related lncRNA. Besides, 318 transcription factor (TF) genes were obtained from Cistrome Cancer database (http://cistrome.org/), whose prediction of TF targeted genes is based on TCGA database and public ChiP-seq profiles. Moreover, a signature matrix of the collated 547 gene expression datasets associated with 22 immune cell types were downloaded from CIBERSORT web portal (Cell type Identification By Estimating Relative Subsets of RNA Transcripts, https://cibersort.stanford.edu/) [20], which encompasses T cells, B cells, macrophages, natural killer cells, dendritic cells, eosinophils, neutrophils, etc.

### 2.2. Differentially Expression of Immune-Related lncRNAs and TF Genes

The expression profiles of lncRNA and mRNA were divided on the basis of GENCODE version 30 (GRCh38, https://www.gencodegenes.org/) by converting Entrez IDs to gene IDs. Then, the gene was excluded if the sum of gene expression level for each sample is less than 1 or it is an unrecognized gene. Finally, 12679 mRNAs and 9063 lncRNAs were obtained. Immune-related gene selected from GSEA and TF gene expression data were extracted from the mRNA profile.

The raw count data of lncRNA and mRNA profiles from TCGA including tumor and normal samples were normalized by TMM normalization using CalcNormFactors function in the “edgeR” package of the R software, which was further used to screen differentially expressed lncRNAs (DElncRNA) and differentially expressed TF genes (DETFs) by comparing expression differences of genes between normal and tumor samples based on the following cutoff values: FDR < 0.01 and absolute log2 fold change (FC) > 1. The raw count data of lncRNA and mRNA profiles from ICGA were normalized by TMM normalization as well for the following validation analysis.

Then, a cohort of immune-related DElncRNAs (IDElncRNAs) was identified according to Pearson correlation analysis between immune-related gene expression level and DElncRNA expression level in all tumor samples (∣*R* | >0.7, *P* < 0.01).

### 2.3. Selection of Prognostic IDElncRNAs

The normalized continuous values of expression data (TMM) were classified into categorical variables (low and high expression, written as “1” and “2,” respectively) according to the median expression value of each IDElncRNA. Then, these categorical values of IDELncRNA and corresponding clinical information from the training set (*n* = 309) were used to identify the prognostic DElncRNAs using univariate Cox proportional hazards regression analysis (hazard ratio (HR) ≠ 1 and *P* < 0.05). Then, the risky prognostic IDElncRNAs (HR > 1) were intersected with the upregulated IDElncRNAs, while the protective prognostic IDElncRNAs (HR < 1) were intersected with the downregulated IDElncRNAs, whose purpose was to filter out the controversial genes, the upregulated IDElncRNAs with HR < 1, and the downregulated IDElncRNAs with HR > 1. Subsequently, these intersected prognosis-related IDElncRNAs were used for further screening analysis.

LASSO (least absolute shrinkage and selection operator) regression was performed to select the more significant prognostic IDElncRNAs and eliminate genes that would overfit the model using “glmnet” package of the R software [21]. Tenfold cross validation was also performed to identify the optimal lambda value from the minimum partial likelihood deviance in order to improve the objectivity and reliability of analysis result. Afterward, the patients from the training set were divided into two groups, high- and low-expression groups by the median cutpoint of expression level of each IDElncRNA, which was determined by “survminer” package of the R software [22]. Then, “survival” package of the R software [23] was performed to implement a log-rank test and draw Kaplan-Meier curves to compare overall survival (OS) between the high- and low-expression levels of IDElncRNAs.

### 2.4. Establishment and Assessment of Multi-lncRNA Signature

The prognostic IDElncRNAs screened from LASSO regression were enrolled in stepwise multivariate Cox proportional hazards regression method, which selected the preferred model with the minimum AIC value and provided the regression coefficients of final prognostic IDElncRNAs. Subsequently, the risk score of each patient was calculated by multiplying categorical value (1 or 2) of expression level (TMM) and coefficient of each PIDElncRNA and summing, which is as follows:

(1) $$\begin{array}{c} \text{Risk score}=\overset{n}{\underset{i=1}{\sum }}\beta_{i}*{\text{CV}}_{i}, \end{array}$$

where *n* is the number of the IDElncRNAs in the best model, CV is the categorical value (1 or 2) of TMM value of each prognostic gene, and *β* is the regression coefficient of it.

The optimal cutoff point of risk score determined by using the “survminer” R package stratified the patients in the training set into high-risk and low-risk groups. Then, the Kaplan-Meier (KM) survival analysis was used to compare the OS between these two groups. Besides, the time-dependent receiver operating characteristic (tROC) curve was performed to evaluate the sensitivity and specificity of the OS prediction using the R package “survivalROC” [24]. In addition, the KM curves were drawn to reveal the diagnostic capacity of the risk score in different subgroups of clinical parameters such as AJCC-stage, Fuhrman-grade, metastasis (yes or no), pharmaceutical (yes or no), gender, and age.

In order to determine whether the risk score could be an independent prognostic marker for prognosis prediction of ccRCC patients, univariate and multivariate Cox proportional hazards regression methods were performed. Other clinical parameters such as age, gender, race, smoking, radiotherapy, pharmaceutical, Fuhrman-grade, AJCC-stage, and TNM were also incorporated in the analysis.

To assess the prediction value of the signature, the testing set (*n* = 203), the whole set (*n* = 512), and the independent external set (*n* = 91) were used to validate the findings. The risk score of each ccRCC patient was obtained by the above formula with the same coefficients used in the training set. Then, the patients in the validation sets were stratified into high-risk and low-risk groups by the same cutoff point in the training set. The KM survival analysis with log-rank test and tROC analysis were performed to validate the signature. In addition, principal component analysis (PCA) was performed using “scatterplot3d” R package [25] to contour the expression pattern of the samples in the training set, the testing set, and the whole set, which visualized whether the high-risk samples and low-risk samples could demonstrate distinctly different immune phenotypes based on the expression of final immune-related lncRNAs via dimensionality reduction. The expression pattern of the high-risk and low-risk samples based on the expression of whole immune-related lncRNA in three sets was plotted as well to compare.

### 2.5. Construction and Evaluation of lncRNA-Based Nomogram

A nomogram based on our lncRNA signature was constructed using “rms” R package [26] to predict the probability of 3-, 5-, and 7-year OS. Other independent prognostic clinical parameters identified by univariate and multivariate Cox proportional hazards regression analyses were also incorporated into the model. To further assess the predictive performance of the prognostic nomogram, the tROC analysis was accomplished and the area under the tROC curve of 3-, 5-, and 7-year OS was calculated, which was also performed in the validation sets. Besides, calibration curve was plotted to visualize the performance of the model with the observed rates of the training set at corresponding time points by a bootstrap method with 1000 resamples.

### 2.6. Immune-Related Functional Enrichment Analysis of Multi-lncRNA Signature

For identifying the biological pathways of the multi-lncRNA signature, Gene Set Enrichment Analysis (GSEA) was performed by the GSEA software (v4.0.3, http://software.broadinstitute.org/gsea/) on JAVA 8.0 platform. The annotated gene set “c2.cp.kegg.v7.0.symbols” and “c5.all.v7.0.symbols” obtained from the Molecular Signatures Database (MSigDB) were conducted as the reference sets to calculate Enrichment Score (ES). The relevant enrichment pathways with normalized *P* value <0.05 and FDR score < 0.25 were considered as significant [27].

For better understanding of infiltrating immune cells in ccRCC microenvironment related to the multi-lncRNA signature, the relative proportion of tumor-infiltrating immune cells was calculated using the CIBERSORT algorithm. Then, the composition fraction of infiltrating immune cells between high-risk group and low-risk group was compared using Wilcoxon test, which considered *P* value <0.05 as significant. Besides, the ICGC dataset was used for validating the relationship above. Moreover, Pearson correlation analysis was performed to figure out the relationship between each ideal lncRNA and significantly infiltration immune cell.

The immune-related biological roles of the final prognostic IDElncRNAs were also investigated through exploring the functional enrichment analysis of coexpressed immune-related mRNA with prognostic IDElncRNAs, in which Gene Ontology (GO) enrichment analysis was performed by online-tool Metascape (http://metascape.org/gp/index.html#/main/step1) [28]. A *P* value of <0.05 was considered to be statistically significant.

As for the important role of transcriptional regulation in gene expression, it is meaningful to explore the relationship between prognostic IDElncRNAs and DETFs. Pearson correlation analysis was performed, and the regulatory relationship was considered as significant when ∣*R* | >0.3 and *P* value<0.01. The visualization of the regulatory network was constructed using Cytoscape V3.7.2. Then, GO analysis was carried out for these coexpressed DETFs by online-tool Metascape. These relationships were validated with ICGC dataset as well.

### 2.7. Statistical Analysis

The R software version 3.6.1 (The R Foundation for Statistical Computing, 2019) was applied to perform all statistical analysis. Volcano plot of DEGs was plotted using “ggrepel” R package [29], while heatmap of DEGs was plotted using “pheatmap” R package [30] with zero-mean normalization. The boxplot between normal and tumor groups was analyzed using Wilcoxon test. For Kaplan-Meier curves, *P* values and hazard ratio (HR) with 95% confidence interval (CI) were generated by log-rank tests and Cox regression methods. The plots of correlation between the expression of lncRNAs and the composition fraction of infiltrating immune cells were drawn by Graphpad Prims 8. All statistical tests were two-sided. *P* value<0.05 was considered as statistically significant.

## 3. Results

### 3.1. Identification of IDElncRNAs and DETFs

**Figure 1:**
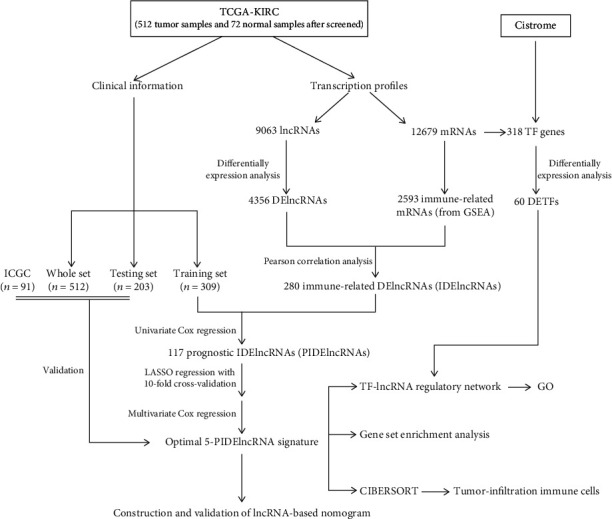
Flowchart of the whole study.

**Figure 2:**
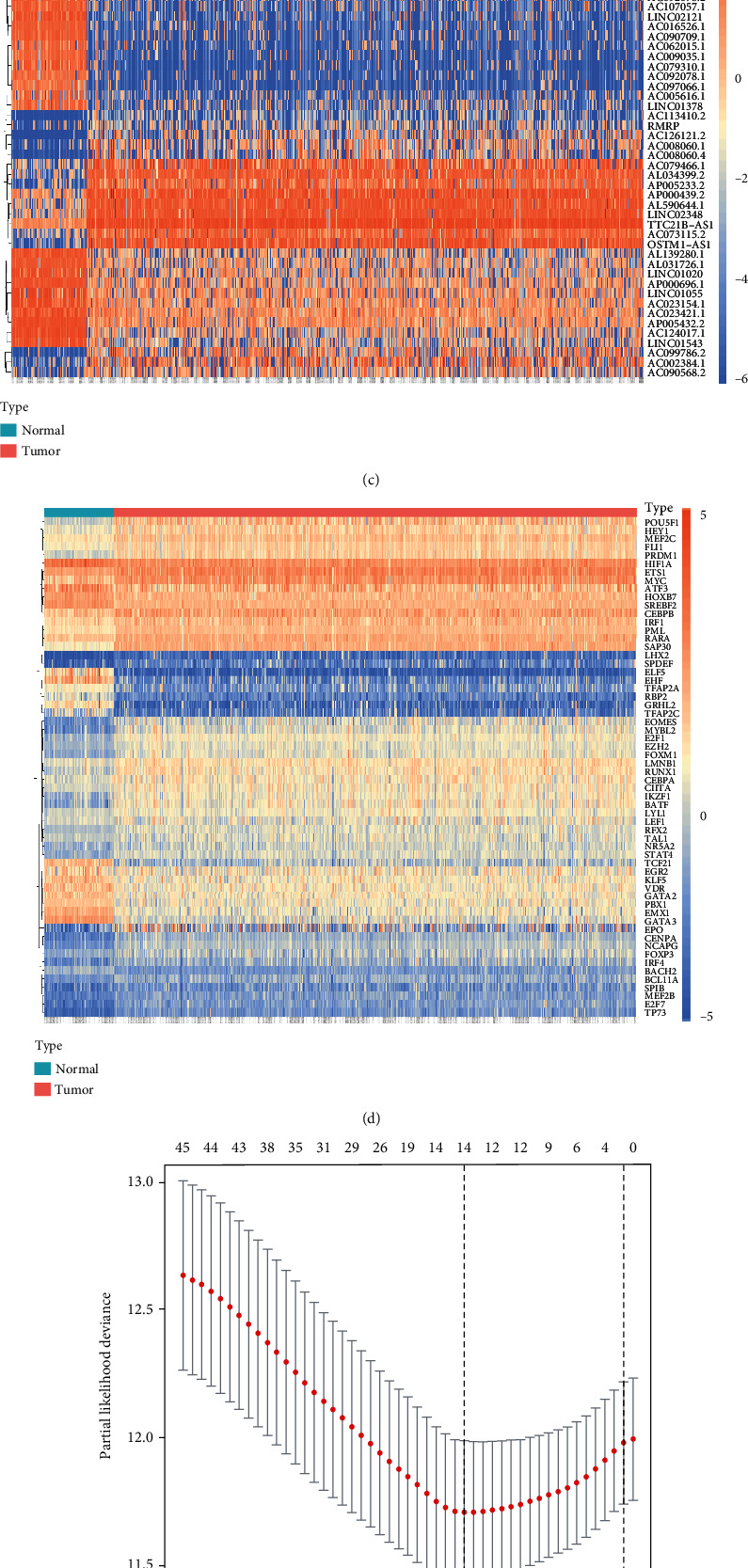
Screening of the DElncRNAs, DETFs (FDR < 0.01 and absolute log2 fold change (FC) > 1), and PIDElncRNAs. (a) Volcano plot of DElncRNAs in ccRCC compared with normal tissues. (b) Volcano plot of DETFs in ccRCC compared with normal tissues. (c) Heatmap of top 50 DElncRNAs in ccRCC. (d) Heatmap of 60 DETFs in ccRCC. (e) LASSO coefficient profiles of 117 PIDElncRNAs. (f) LASSO regression with tenfold cross validation obtained 14 PIDElncRNAs using minimum lambda value. (g) Multivariate Cox regression analysis of 5 PIDElncRNAs. DElncRNAs: differentially expressed lncRNAs; DETFs: differentially expressed transcription factor genes; PIDElncRNAs: prognostic immune-related differentially expressed lncRNAs; FDR: false discovery rate; ccRCC: clear cell renal cell carcinoma; LASSO: least absolute shrinkage and selection operator. ^∗^*P* < 0.05, ^∗∗^*P* < 0.01, and ^∗∗∗^*P* < 0.001.

The flowchart of the whole study is presented in Figure 1. After processing the data, a total of 4356 DElncRNAs and 60 DETFs were identified with the criteria of FDR < 0.01 and ∣log2FC | >1 (Figures 2(a) and 2(b)). The heatmaps of top 50 DElncRNAs and 60 DETFs were shown as well (Figures 2(c) and 2(d)). Then, 280 immune-related DElncRNAs (IDElncRNAs) which included 254 upregulated and 26 downregulated genes were screened by the correlation analysis with 2593 immune-related genes from GSEA with the criteria of ∣*R* | >0.7 and *P* value<0.05. The 280 IDElncRNAs were recorded in Table S3 (Supplementary material 3).

### 3.2. Screening of Prognostic IDElncRNA

The processed survival data of each tumor sample in the training set were subjected to univariate Cox proportional hazards regression analysis, in which the significant threshold was set at *P* value<0.05. Therefore, 166 prognosis-related IDElncRNAs (PIDElncRNAs) containing 136 risky PIDElncRNAs (HR > 1) and 30 protective PIDElncRNAs (HR < 1) were identified in total, which were used to intersect with 254 upregulated IDElncRNAs and 26 downregulated IDElncRNAs, respectively. Finally, 49 controversial PIDElncRNAs were filtered out, and 116 risky prognostic with upregulated IDElncRNAs and 1 protective prognostic with downregulated IDElncRNAs were discovered.

LASSO regression with tenfold cross validation was performed to further screen the IDElncRNAs that significantly correlated with the prognosis of ccRCC patients. The optimal lambda value was obtained from the minimum partial likelihood deviance (*λ*_min_ = 0.00031) (Figures 2(e) and 2(f)). Therefore, 14 PIDElncRNAs derived from this regression method were used in subsequent stepwise multivariate Cox proportional hazards regression analysis, and finally, the optimal 5-PIDElncRNA model was obtained with the lowest AIC value (Figure 2(g)), which contained AC012236.1, AC078778.1, AC078950.1, AC087318.1, and AC092535.4.

### 3.3. Expression Profiles and Survival Analysis of the Optimal 5 IDElncRNAs

**Figure 3:**
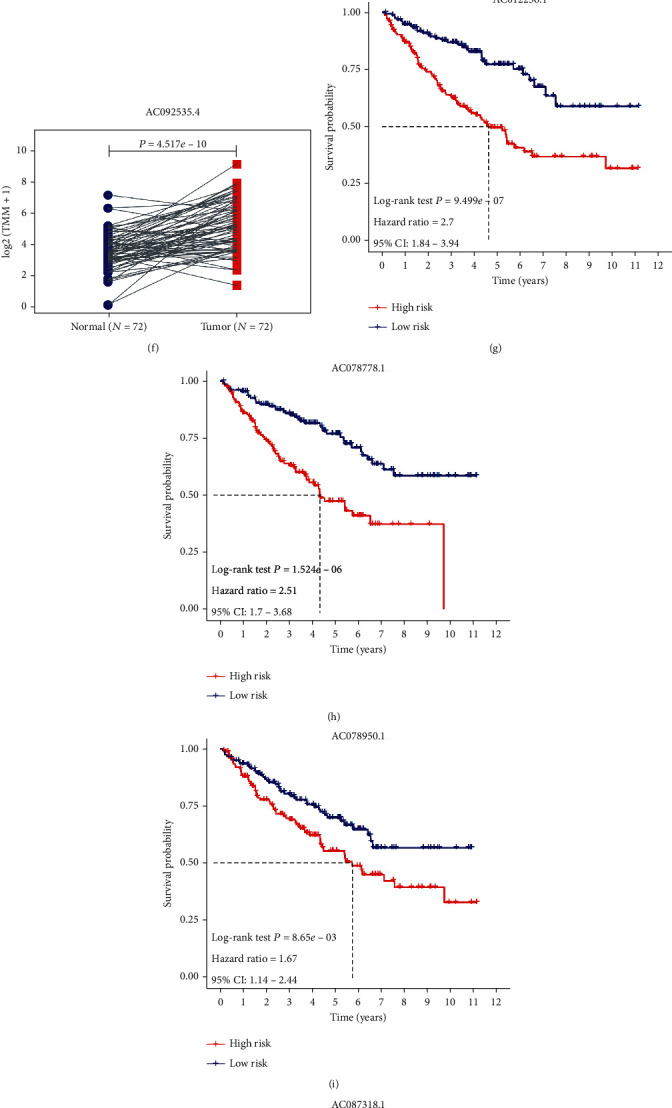
Expression pattern and Kaplan-Meier survival analysis of the five PIDElncRNAs. (a) Expression pattern of the five PIDElncRNAs between 72 normal samples and 512 tumor samples. (b–f) Paired expression pattern of the five PIDElncRNAs between 72 normal samples and 72 tumor samples. (g–k) Survival analysis of the five PIDElncRNAs. PIDElncRNAs: prognostic immune-related differentially expressed lncRNAs; ^∗^*P* < 0.05, ^∗∗^*P* < 0.01, and ^∗∗∗^*P* < 0.001.

The expression profiles of the optimal 5 PIDElncRNAs between 512 carcinoma and 72 normal tissues were shown in Figure 3(a), which indicated that the 5 PIDElncRNAs all significantly upregulated in the ccRCC (*P* < 0.05).Besides, the expression levels of the five genes between paired 72 normal tissues and 72 carcinoma tissues from the same patient were also illustrated in Figures 3(b)–3(f). Furthermore, the relationship between the expression levels and histopathological information including AJCC-stage and tumor grade was shown in Figure S1A-J (Supplementary material 4). Among the 5 PIDElncRNAs in ccRCC, the expression of AC012236.1, AC078778.1, AC087318.1, and AC092535.4 was significantly associated with AJCC-stage (*P* < 0.001) and tumor grade (*P* < 0.001).

The prognostic value of the final 5 IDElncRNAs was reflected by KM survival curves in Figures 3(g)–3(k), where the median expression value of each IDElncRNA was regarded as a cutoff to partition the training set samples into high- and low-expression groups. Overexpression of the 5 IDElncRNAs was associated with the poor prognosis of ccRCC patients, which meant that they were all risky prognosis-related IDElncRNAs. The KM survival curves of other 9 prognostic IDElncRNAs screened from LASSO regression method were shown in Figure S3 (Supplementary material 6).

### 3.4. Establishment, Estimation, and Validation of the 5-PIDElncRNA Signature

**Figure 4:**
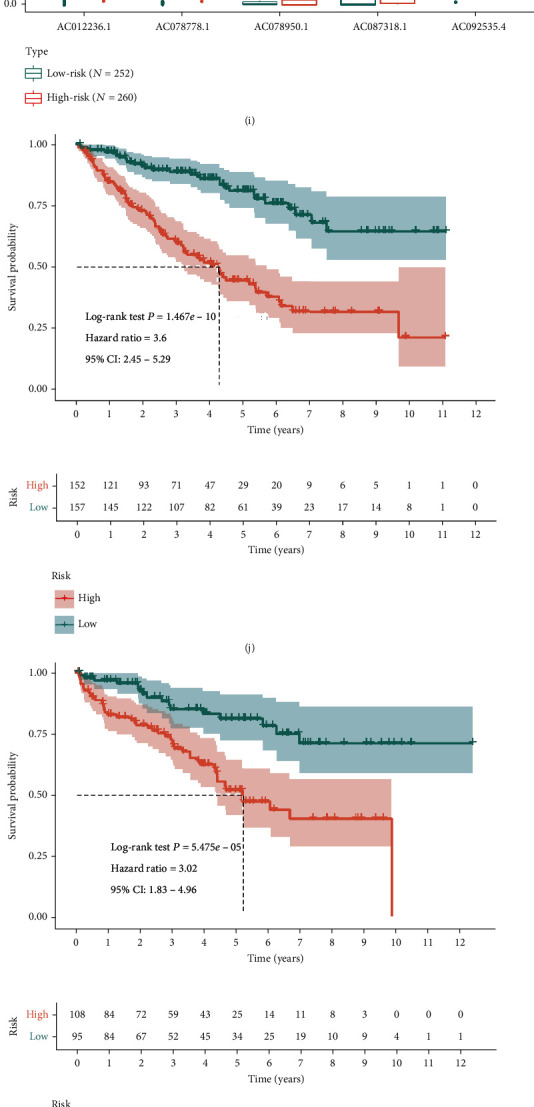
Prognostic analysis of the five-lncRNA signature in three sets. (a–c) The curves of risk score in the training set, the testing set, and the whole set. The dotted line represents the cutoff score and divides the patients into the low-risk and high-risk groups. (d–e) Survival status of the patients in the training set, the testing set, and the whole set. The orange dots represent dead patients while the green dots represent alive patients. More dead patients correspond to the higher risk score. (g–i) Boxplots of the expression profiles of the five PIDElncRNAs in the training set, the testing set, and the whole set. (j–l) Kaplan-Meier survival analysis of the five-lncRNA signature in the training set, the testing set, and the whole set. (m–o) Time-dependent ROC curves of the five-lncRNA signature in the training set, the testing set, and the whole set. PIDElncRNAs: prognostic immune-related differentially expressed lncRNAs; ROC: receiver operating characteristic.

**Figure 5:**
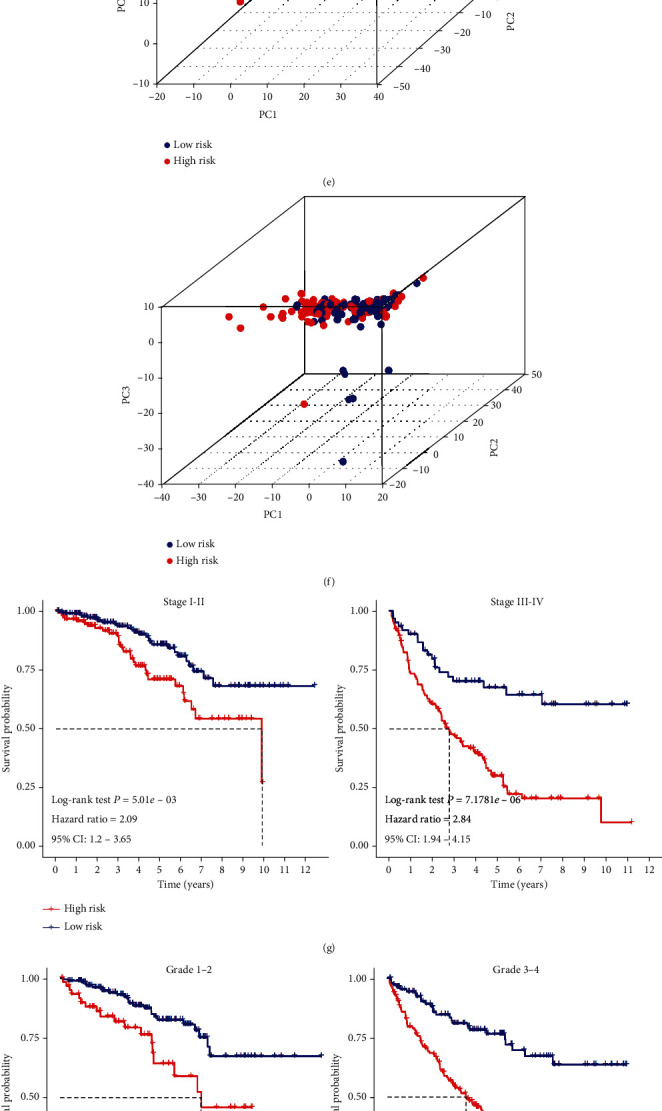
Principal component analysis of three sets and Kaplan-Meier survival analysis of the five-lncRNA signature in different subgroups of clinical parameters. (a–c) Principal component analysis of the training set, the testing set, and the whole set based on the 5-lncRNA expression. (d–f) Principal component analysis of the training set, the testing set, and the whole set based on the whole immune-related lncRNA expression. The red dots represent the high-risk patients, while the blue dots represent the low-risk patients. (g–l) Survival analysis of the five-lncRNA signature in stages I and II, stages III and IV, grades 1 and 2, grades 3 and 4, metastasis, without metastasis, pharmaceutical, without pharmaceutical, male, female, elder, and younger.

The Cox coefficients of the 5 PIDElncRNAs were obtained from the multivariate Cox proportional hazards regression analysis. Then, the risk score was constructed based on the coefficients and categorical values of expression level as the following: risk score = 0.615626∗CV_(AC012236.1)_ + 0.641071∗CV_(AC078778.1)_ + 0.455388∗CV_(AC078950.1)_ + 0.421934∗CV_(AC087318.1)_ + 0.599496∗CV_(AC092535.4)_. The median expression values of AC012236.1, AC078778.1, AC078950.1, AC087318.1, and AC092535.4 were as cutoff points, and they were 14.17589, 13.3747, 0.071091, 0.103646, and 49.81774, respectively (expression values are TMM). The risk score of each tumor patient in the training set was calculated. Next, the patients in the training set were divided into high-risk group and low-risk group based on the cutoff of the risk score determined by “survminer” R package (Figure 4(a)). The correlated survival status of the patients in the training set was shown in Figure 4(d), which suggested that more patients got dead in the high-risk group. The boxplot of the expression of 5 PIDElncRNAs in the training set was shown in Figure 4(g), which showed that the expression levels of these five lncRNAs were higher in the high-risk group when compared with that of the low-risk group. The high-risk group patients had worse OS than that of low-risk group patients plotted in Figure 4(j) by KM survival analysis. In order to validate the 5-PIDElncRNA signature, the testing set (Figures 4(b), 4(e), 4(h), and 4(k)) and the whole set (Figures 4(c), 4(f), 4(i), and 4(l)) were used to do the above analysis, whose results were consistent with that of the training set. Besides, the independent external dataset, ICGC, was used for verifying the signature as well in Figure S2A (Supplementary material 5). Moreover, the tROC analysis was performed, and the AUC values of 3-, 5-, and 7-year OS in the training set were 0.755, 0.772, and 0.751, respectively (Figure 4(m)), in the meanwhile, the AUC values of 3-, 5-, and 7-year OS in the internal validation set, the whole set, and the external validation set also suggested good performance of the signature (Figures 4(n) and 4(o); Supplementary material 5: Figure S2B). Besides, PCA of the training testing and whole set showed that the high-risk and low-risk samples clustered separately in three-dimensional space based on the 5-PIDElncRNA expression (Figures 5(a)–5(c)). However, there was no observable separation between high-risk and low-risk samples on the basis of the whole immune-related lncRNA expression profiles (Figures 5(d)–5(f)). The result demonstrated a distinguishing distribution pattern of the high-risk and low-risk groups grounded on the immune-related signature, reflecting that our constructed signature had more discriminatory ability to identify the difference in immune phenotype among the samples when compared to the whole immune-related lncRNA expression profiles.

We performed risk stratification in patients with different subgroups of AJCC-stage, Fuhrman-grade, gender, age, metastasis, and pharmaceutical and did the KM survival analysis (Figures 5(g)–5(l)). The patients with high-risk scores had worse OS than the patients with low-risk scores in stages I and II, stages III and IV, grades 1 and 2, grades 3 and 4, metastasis yes or no, no pharmaceutical, male, female, younger, and elder subgroup (*P* < 0.05), while there was no difference in pharmaceutical subgroup (Supplementary material 6: Figure S3A-J). The comparisons of risk score in different AJCC-stage and grade were also shown in Figure S3K-L (Supplementary material 6), which indicates that the higher risk score, the more advanced of the carcinoma is.

### 3.5. Construction and Validation of the lncRNA-Based Nomogram

**Figure 6:**
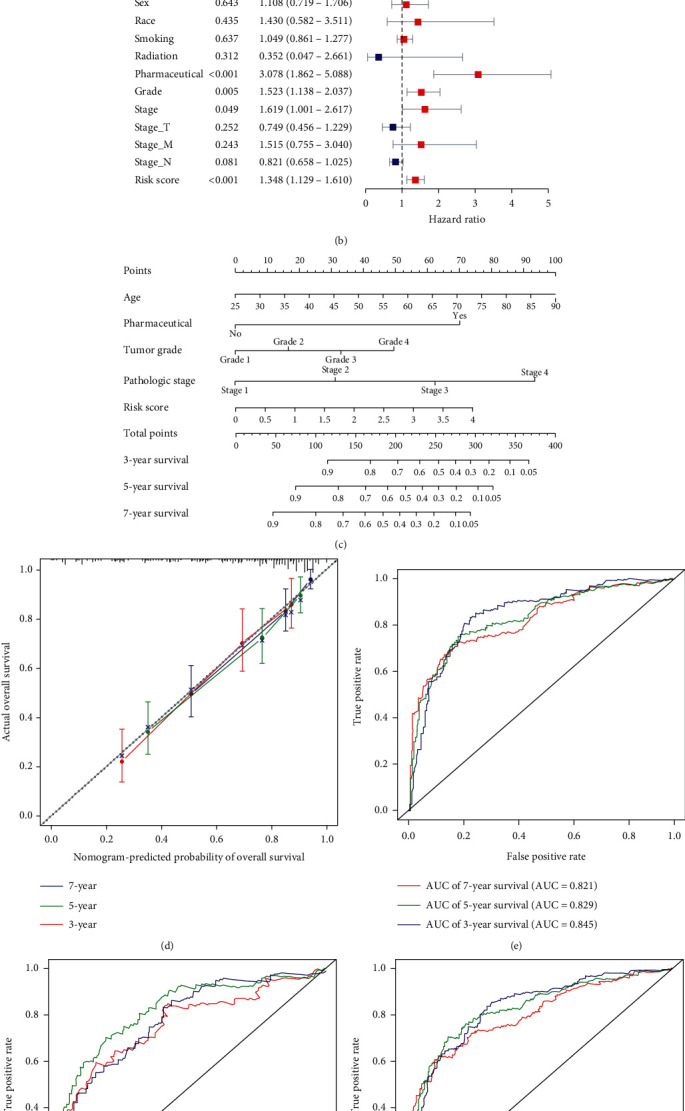
Identifying independent prognostic parameters and constructing a lncRNA-based nomogram. (a) Forrest plot of univariate Cox regression analysis in ccRCC. (b) Forrest plot of multivariate Cox regression analysis in ccRCC. (c) A lncRNA-based nomogram integrating AJCC-stage, Fuhrman-grade, age, pharmaceutical, and risk score to predict 3-, 5-, and 7-year OS of ccRCC patients. (d) The calibration plot of the nomogram for predicting 3-, 5- and 7-year OS of ccRCC patients. E-G, Time-dependent ROC curves of the nomogram for 3-, 5-, and 7-year OS prediction in the training set, the testing set, and the whole set. AJCC: American Joint Committee on Cancer; OS: overall survival; ccRCC: clear cell renal cell carcinoma; ROC: receiver operating characteristic.

In order to build a more individualized and applicable predictive model, a good performance of prediction nomogram was constructed based on the 5-PIDElncRNA signature. The 5-PIDElncRNA signature and other clinical parameters such as AJCC-stage, age, pharmaceutical, and Fuhrman-grade could be an independent prognostic factor, respectively, for OS prediction of ccRCC patients in the training set after the univariate and multivariate Cox proportional hazards regression analyses (Figures 6(a) and 6(b)). Then, these independent prognostic factors were integrated together into this nomogram to predict 3-, 5-, and 7-year OS of ccRCC patients (Figure 6(c)).

The calibration plot for predicting 3-, 5-, and 7-year OS showed that the lncRNA-based nomogram exhibited excellent performance with high agreement between model predicted outcome and actual outcome (Figure 6(d)). Besides, the tROC curves were drawn, and the AUC values of the nomogram at 3-, 5-, and 7-year were 0.845, 0.829, and 0.821, respectively (Figure 6(e)).

To further validate the predictive value of the 5-PIDElncRNA prognostic nomogram, the testing set and the whole set were used to perform the tROC analysis and test the results which were from the training set. The AUC values of the testing and whole set at 3-, 5-, and 7-year OS were 0.803, 0.833, 0.770, 0.826, 0.825, and 0.796, respectively (Figures 6(f) and 6(g)).

### 3.6. Functional Enrichment Analysis and Associated Immune Cell Infiltration of the 5-PIDElncRNA Signature

**Figure 7:**
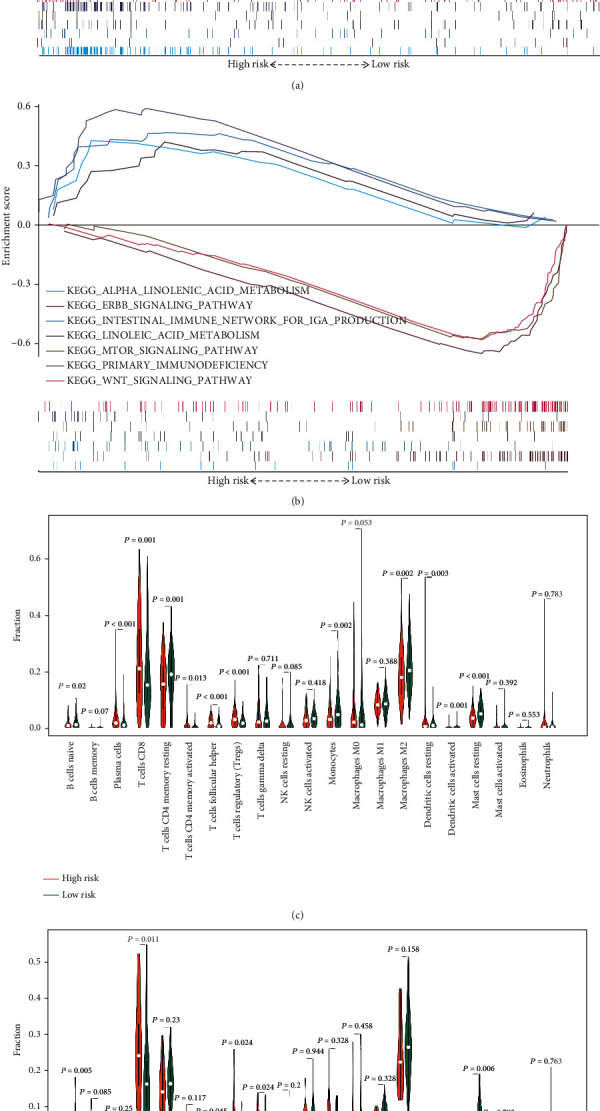
GSEA and the relation of the tumor-infiltrating immune cells to the five-lncRNA signature. (a) Immune-related GO terms enriched in the high-risk group. (b) Associated KEGG terms enriched in the high-risk and low-risk groups. (c) Comparison of tumor-infiltrating immune cell proportion between the high-risk group and low-risk group in TCGA dataset. (d) Comparison of tumor-infiltrating immune cell proportion between the high-risk group and low-risk group in ICGC dataset. GSEA: Gene Set Enrichment Analysis; GO: Gene Ontology; KEGG: Kyoto Encyclopedia of Genes and Genomes.

The biological function of the 5-PIDElncRNA signature was identified by GO and KEGG enrichment analyses using GSEA. In GO biological analysis, the signature was enriched in some immune-related biological function, such as immune response to tumor cell, natural killer cell activation involved in immune response, regulation of humoral and adaptive immune response, and T cell activation and differentiation involved in immune response (Figure 7(a)). In KEGG pathway analysis, intestinal immune networks for IgA production, primary immunodeficiency, ERBB signaling pathway, MTOR signaling pathway, WNT signaling pathway, etc. were identified for the 5-PIDElncRNA signature (Figure 7(b)). Furthermore, the GO and KEGG functional enrichment analyses of each lncRNA of the ideal five lncRNAs were explored by GSEA and shown in Figure S4 (Supplementary material 7). The potential function of AC012236.1 was enriched in “regulation of humoral immune response” biological process and involved in “JAK-STAT signaling pathway” in ccRCC (Supplementary material 7: Figure S4A, F). AC078778.1 was related to “negative regulation of production of molecular mediated of immune response” and revealed to be associated with “homologous recombination pathway” (Supplementary material 7: Figure S4B, G). For AC078950.1, “pre-mRNA 5 splice site binding” was enriched by GO analysis and the pathway, “Nod-like receptor signaling pathway,” was disclosed by KEGG analysis (Supplementary material 7: Figure S4C, H). Highly expressed AC087318.1 was linked to the biological process “regulation of T cell differentiation” while its involved KEGG pathway was “Toll-like receptor signaling pathway” (Supplementary material 7: Figure S4D, I). AC092535.4 was suggested to be associated with “DNA polymerase complex” biological characteristic, and high expression of it probably played a critical role in “p53 signaling pathway” according to what we found (Supplementary material 7: Figure S4E, J).

In order to determine whether the 5-PIDElncRNA signature was associated with immune cell infiltration in tumor immune microenvironment, the infiltrating immune cell composition fraction between the high-risk group and low-risk group was compared in TCGA and ICGC cohorts (Figures 7(c) and 7(d)). As shown in the figure, the fraction of CD8^+^ T cells, regulatory T cells, T follicular helper cells, resting mast cells, and naïve B cells was significantly different between the high-risk and low-risk groups in both datasets. The proportion of CD8^+^ T cells, regulatory T cells, and T follicular helper cells was significantly higher in the high-risk group compared with that of the low-risk group, while the proportion of naïve B cells and resting mast cells was significantly lower in the high-risk group. Because the composition fraction of naïve CD4^+^ T cells calculated in 22 immune cell types from TCGA and ICGA datasets was zero, it was dislodged in the figure. In order to specify the correlation between the signature and infiltrating immune cells, Pearson correlation analysis was performed between each one of the five lncRNAs and significantly infiltrating immune cells (Supplementary material 8: Figure S5). AC012236.1 was correlated positively with the infiltration of regulatory T cells (*R* = 0.2547, *P* < 0.0001) and T follicular helper cells (*R* = 0.1733, *P* = 0.0007), while it was correlated negatively with the infiltration of resting mast cells (*R* = −0.3050, *P* < 0.0001). AC078778.1 was positively correlated with the infiltration of regulatory T cells (*R* = 0.2556, *P* < 0.0001), T follicular helper cells (*R* = 0.3336, *P* < 0.0001), and CD8^+^ T cells (*R* = 0.2346, *P* < 0.0001), but negatively correlated with resting mast cells (*R* = −0.1822, *P* = 0.0003) and naïve B cells (*R* = 0.1479, *P* = 0.0037). As for AC087318.1, it was negatively associated with naïve B cells (*R* = −0.2442, *P* < 0.0001) and resting mast cells (*R* = −0.3083, *P* < 0.0001) and positively associated with CD8+ T cells (*R* = 0.6696, *P* < 0.0001) and T follicular helper cells (*R* = 0.4489, *P* < 0.0001). Besides, AC092535.4 was found to be related positively with regulatory T cells (*R* = 0.2448, *P* < 0.0001) and negatively with resting mast cells (*R* = −0.1604, *P* = 0.0016). However, the relationship between AC078950.1 and the infiltration immune cells could not be disclosed significantly.

### 3.7. Construction and Functional Annotations of Immune-Related TF-lncRNA Regulatory Relationship Network

**Figure 8:**
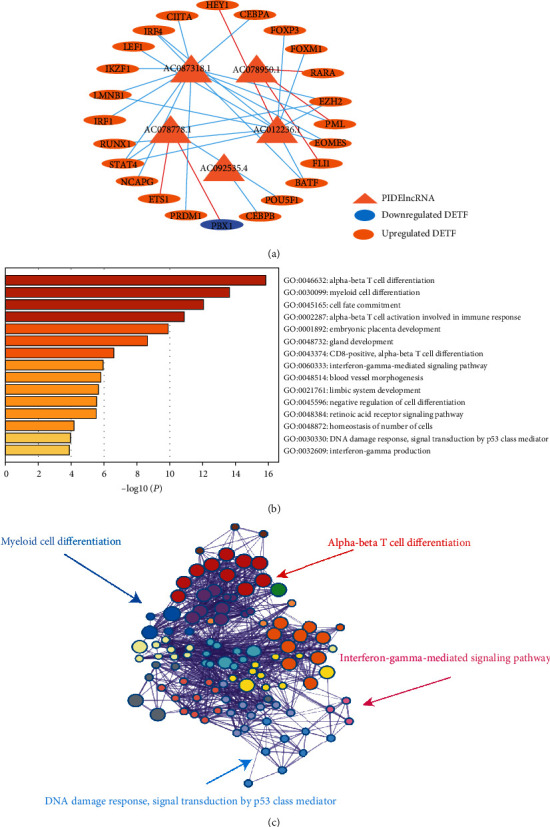
Regulatory relationship network of DETFs and five PIDElncRNAs and GO enrichment analysis. (a) The network of DETFs and five PIDElncRNAs based on TCGA and ICGC datasets. The orange triangles represent PIDElncRNAs; the orange nodes represent upregulated DETFs, while the blue nodes represent downregulated DETFs; the blue lines indicate positive regulatory relationships, while the red lines indicate negative regulatory relationships. (b) The relevant GO enrichment pathways of the coexpressed DETFs used in the lncRNA-TF regulatory relationship network. (c) The network of enriched GO terms for the coexpressed DETFs. DETFs: differentially expressed transcription factor genes; PIDElncRNAs: prognostic immune-related differentially expressed lncRNAs; GO: Gene ontology.

Transcription factor plays a critical role in controlling gene expression including lncRNAs. Therefore, it is significative to explore possible mechanisms that cause dysregulation of the 5 PIDElncRNAs. The coexpression analysis was performed to identify the TF-lncRNA pairs (Supplementary material 9: Table S4). ICGC dataset was also used to validate the correlation relationship between the DETF and PIDElncRNAs (Supplementary material 10: Table S5). The common TF-lncRNA pairs between TCGA and ICGC datasets were screened for constructing the DETFs-PIDElncRNA regulatory network (Figure 8(a)). From this regulatory relationship network, the expression of BATF (*r* = 0.209), EOMES (*r* = 0.207), EZH2 (0.246), FOXM1 (*r* = 0.297), IRF4 (*r* = 0.391), LEF1 (*r* = 0.284), LMNB1 (*r* = 0.347), RUNX1 (0.225), and STAT4 (0.325) was correlated positively with the expression of AC012236.1, while the expression of HEY1 (*r* = −0.429) was correlated negatively with that of AC012236.1, which speculates that the expression of AC012236.1 is probably regulated by these TFs. The expression of EZH2 (*r* = 0.332), POU5F1 (*r* = 0.446), and STAT4 (*r* = 0.318) was correlated positively with that of AC078778; the expression of ETS1 (*r* = −0.071) and PBX1 (*r* = −0.245) was correlated negatively with that of AC078778.1. FLI1 (*r* = −0.269), PML (*r* = −0.169), and RARA (*r* = −0.313) expression was negatively associated with AC078950.1. Besides, the expression of BATF (*r* = 0.474), CEBPA (*r* = 0.183), CIITA (*r* = 0.276), EOMES (*r* = 0.534), EZH2 (*r* = 0.259), IKZF1 (*r* = 0.257), IRF1 (*r* = 0.297), IRF4 (*r* = 0.452), LMNB1 (*r* = 0.273), NCAPG (*r* = 0.299), PML (*r* = 0.231), PRDM1 (*r* = 0.192), and STAT4 (*r* = 0.242) was positively related to the expression of AC087318.1, and in the meanwhile, the expression of CEBPB (*r* = 0.191) was positively related to that of AC092535.4. The regulatory order between these DETFs and PIDElncRNAs is not certain, and we just surmise that these TFs probably play a role in regulating the expression of the five lncRNAs based on the correlation analysis.

Moreover, GO enrichment analysis of the DETFs identified transcriptional and immune-related processes such as alpha-beta T cell differentiation, myeloid cell differentiation, and interferon-gamma-mediated signaling pathway (Figure 8(b)). The corresponding network of biological process was also shown (Figure 8(c)).

## 4. Discussion

ccRCC is the most common type of renal cancer accounting for 2% of the global cancer burden, while its incidence is on the rise [31]. Immunotherapy such as anti-CTLA-4 antibody and anti-PD-1 antibody has been identified as promising strategy for ccRCC [18, 32]. However, only a fraction of patients shows durable responses, which indicates that alternative mechanism restricts the immune response and helps cancer escape the immunosurveillance. lncRNAs have been considered as a critical part in cancer immunotherapy as they could mediate innate and adaptive immunity by regulating immune response genes [33]. Rising evidence has shown that aberrant expression of lncRNAs would affect the prognosis of cancer patients. Dysregulated expression of lncRNAs would contribute to oncogenesis through giving impact on many biological processes [34]. However, the character of lncRNAs in immune response for ccRCC stays at a preliminary stage. Therefore, it is meaningful to screen ideal immune-related lncRNAs as biomarkers and construct a prognostic model to predict the prognosis of ccRCC patients, which could provide more hints for further exploring the novel immunotherapy of ccRCC.

In this study, novel prognostic immune-related lncRNAs were identified through differentially expressed analysis, univariate Cox proportional hazards regression, LASSO with tenfold cross validation, and multivariate Cox proportional hazards regression. Finally, 5 ideal novel risky PIDElncRNAs, AC012236.1, AC078778.1, AC078950.1, AC087318.1, and AC092535.4, were selected, whose biological function and specific roles in cancer have not been investigated widely and deeply (Supplementary material 11: Table S6). AC078778.1 is a novel transcript and sense overlapping to COPZ1 and HNRNPA1 [35]. It was found to be differentially expressed in bladder urothelial cancer (BUC) and positively related to its OS, which could be an independent prognostic factor for OS of BUC [36]. Hu et al. reported that AC078778.1 was upregulated in lung squamous cell carcinoma (LUSC), and they identified a three-lncRNA signature containing AC078778.1 as a potentially prognostic biomarker for LUSC [37]. Liu and Ye also discovered that it was differentially expressed in laryngeal squamous cell carcinoma [38]. But the specific mechanism that how dysregulated AC078778.1 gives rise to oncogenesis, especially in ccRCC, remains unclear, and it is necessary to be explored further. Our study provides some evidence that it was an immune-related risky prognostic lncRNA for ccRCC patients, and it would be related to negative regulation of production of molecular mediator of immune response and probably plays an important role in homologous recombination signaling pathway in ccRCC carcinogenesis and promotion according to GSEA (Supplementary material 7: Figure S4A,F Besides, the correlation analysis also suggested that the expression of AC078778.1 was correlated positively or negatively with regulatory T cells, T follicular helper cells, resting mast cells, CD8^+^ T cells, and naïve B cells. AC092535.4, an alias for LOC105374344 [35], was found to be one of top 10 upregulated lncRNAs in endometrial cancer [39]. As far as we know, no more research related to AC092535.4 was reported. Based on our findings, AC092535.4 was upregulated in ccRCC and its high expression level was associated with the poor survival of ccRCC patients (Figure 3), which was probably due to its facilitating role in tumorigenesis and tumor promotion (Supplementary material 4: Figure S1I-J). Besides, it was revealed that it probably exerts its role in DNA polymerase complex from GO biological analysis and P53 signaling pathway from KEGG pathway analysis (Supplementary material 7: Figure S4E, J). Moreover, its expression was correlated positively and negatively with regulatory T cells and resting mast cells, respectively. As for AC012236.1, AC078950.1, and AC087318.1, the relative information could not be available because of the little research on these three novel lncRNAs. What we found was that they were all immune-related risky prognostic lncRNAs in ccRCC. They were related to the advance of ccRCC except AC078950.1 (Supplementary material 5: Figure S2 Moreover, based on our discovery, AC012236.1 would probably make a difference in regulation of humoral immune response and also involve in JAK-STAT signaling pathway in ccRCC (Supplementary material 7: Figure S4A, F); AC078950.1 played an important role in the biological process about pre-mRNA 5 splice site binding and Nod-like receptor signaling pathway in ccRCC development (Supplementary material 7: Figure S4C, H); AC087318.1 potentially emitted its effect in regulation of T cell differentiation process and Toll-like receptor signaling pathway (Supplementary material 7: Figure S4D, I). In addition to this, Pearson correlation analysis revealed that their expression was correlated positively or negatively with some infiltration immune cells in ccRCC microenvironment except AC078950.1, such as regulatory T cells, T follicular helper cells, resting mast cells, CD8^+^ T cells, and naïve B cells (Supplementary material 8: Figure S5). Because of their significant prognostic value for ccRCC patients, immune-related biological function and correlation with infiltrating immune cells and their specific mechanism inducing oncogenesis are deserved to be explored in the future.

Transcription factors (TFs) play a critical role in regulating gene expression including lncRNA. The dysregulated expression of TFs and their downstream targets have been demonstrated to be associated with carcinogenesis [40]. For instance, as reported by Fan et al., Octamer-binding TF4 protein exerts a promoting effect in osteosarcoma through regulating lncRNA AK055347 expression [41]. Besides, lncRNAs are also able to interact with TF and affect their regulatory function [42]. Hung et al. proved that PANDA, a lncRNA transcribed from CDKN1A promoter, can interact with the transcription factor NF-YA to limit expression of proapoptotic genes [43]; Willingham et al. suggested that the noncoding repressor of NFAT (nuclear factor of activated T cell) lncRNA can form a complex with some other proteins to repress the transcription factor NFAT [44]. Although the roles of lncRNAs in regulating TF activities have been inferred by these studies, the direct physical interaction has not been reported [45]. Therefore, in our study, we simply analyzed the correlation relationship between the five lncRNAs and DETFs to construct the TF-lncRNA regulatory network, which probably reveals the underlying interactions between them and transcriptional mechanism of the five lncRNAs. This would potentially provide the foundation for the future research on the mechanism of ccRCC development. Moreover, the GO analysis of DETF revealed the possible transcriptional mechanisms and immune-related biological function which further proved the immune-related properties of 5 lncRNAs.

Then, these 5 ideal PIDElncRNAs were used to establish an immune-related multi-lncRNA signature to evaluate the prognosis of ccRCC patients through risk stratification. The patients in high-risk group showed poorer prognosis compared with the patients in low-risk groups, and the internal validation set verified the prognostic value. The tROC curves of the signature in three sets suggested the good prognostic value of the immune-related signature, especially in 5- and 7-year OS of ccRCC patients. Then, in order to investigate the wide applicability of the immune-related signature, the associations between the risk score and different clinicopathological parameters were evaluated. It was observed that the signature could accurately stratify the patients into the high-risk group with shorter OS and the low-risk group with longer OS in different subgroups such as AJCC-stage, Fuhrman-grade, metastasis, pharmaceutical, gender, and age. Meanwhile, the high-risk group could show advanced stage and malignant grade (Supplementary material 4: Figure S1k-l). These findings suggest that the signature is tightly associated with progression and poor outcome of ccRCC. In order to improve the ability of prognosis prediction, the signature and other independent prognostic factors (age, pharmaceutical, Fuhrman-grade, AJCC-stage, and risk level) screened from univariate and multivariate Cox regression analyses were integrated together to construct a highly accurate predictive nomogram. The 3-, 5-, and 7-year OS of individual ccRCC patients could be predicted by the nomogram. Then, tROC analysis of the nomogram in three sets showed great power to predict the OS of ccRCC patients. Perfect agreement was also observed in the calibration plot between the predicted and observed outcomes. Therefore, the immune-related five-lncRNA-based nomogram may help clinicians predict the 3-, 5-, and 7-year OS of ccRCC patient personally and accurately.

Our 5-PIDElncRNA signature was shown to have robust connection to immune response by GO terms of GSEA. The high-risk group was more related to immune response to tumor cells, T cell activation and differentiation involved in immune response, regulation of humoral and adaptive immune response, and natural killer cell activation involved in immune response. These findings suggest that the patients in the high-risk group based on this immune-related signature would easily get ccRCC formation and progression as the result of the affected immune system. Therefore, it is necessary to further study the specific immune-related mechanism behind carcinogenesis.

Recently, emerging studies have corroborated that genetic aberrations in tumor cells can affect the immune landscape of tumor [46, 47]. Some research has demonstrated that tumor-infiltrating immune cells were critical for the therapeutic responsiveness and prognosis of cancer including ccRCC [48, 49]. Liu et al. stated that tumor-infiltrating immune cells would activate the immune response and promote cancer progression in ccRCC [50]. It is believed that CD8^+^ T cells play an important role in tumor control [51]. However, Nakano et al. found that the overall and disease-free survival rates were shorter in RCC patients with abundant intratumoral CD8^+^ T cells than in those having a small number of intratumoral CD8^+^ T cells [52], while Nicolas et al. revealed that only with the presence of functional mature dendritic cells recruited CD8^+^ T cells correlated with favorable prognosis in RCC [53]. In our study, we analyzed the relationship between the risk score and tumor-infiltrating immune cells and found that the high-risk group had higher proportion of CD8^+^ T cells, T follicular helper cells, and regulatory T cells and lower proportion of naïve B cells and resting mast cells in ccRCC microenvironment compared with the low-risk group, which revealed the immunological environment of ccRCC is associated with our immune-related signature. Besides, the correlation between each lncRNA and these significantly infiltrated immune cells was analyzed and discussed above, which further specifies the relationship between the signature and immune microenvironment. These results suggested that on the basis of our risk score model, the tumor immune microenvironment in ccRCC could be conjectured that which kind of immune cells would be more likely to infiltrate, which may offer important insights into tumor-immune interactions of ccRCC and provide the basis and evidence for future novel immunotherapy in ccRCC.

Although we have identified several ideal novel immune-related lncRNAs which could be biomarkers for ccRCC and constructed an integrated nomogram with good performance in prognosis prediction of ccRCC patients, several limitations of our study should be acknowledged as follows. Firstly, to our best of knowledge, the expression data and clinical information of 5 novel lncRNAs are not available from other databases such as Gene Expression Omnibus (GEO) or cBioPortal. Therefore, the validation of our model was only verified by the internal testing set and one independent external validation set. Secondly, our study was based on the publicly databases. With these two concerning, we intended to collect samples from ccRCC patients in our hospital for experimental analysis like RNA-sequencing analysis and flow cytometry analysis to explore the concrete mechanism of the five lncRNAs in immune system and to discover the potential immune-related mechanisms of our signature, which might provide evidence for clinical application in the future.

## 5. Conclusion

In summary, we identified five novel prognostic immune-related lncRNAs through mining publicly databases and constructed a powerful five-lncRNA based nomogram to predict 3-, 5-, and 7-year OS of ccRCC patients, which was probably used to guide clinicians in decisions of clinical diagnosis, prognosis, and treatment. Functional enrichment analysis and relation to tumor-infiltrating immune cells reveal immune-related characteristics of each lncRNA and the multi-lncRNA signature, which may provide new insights into immunotherapy for ccRCC.

## Data Availability

The datasets generated and analyzed during the current study are available in The Cancer Genome Atlas (TCGA), https://portal.gdc.cancer.gov/, International Cancer Genome Consortium, https://icgc.org/, Cistrome Cancer database, http://cistrome.org/, and CIBERSORT, https://cibersort.stanford.edu/.

## Supplementary Materials

Supplementary 1 Table S1: clinical information of TCGA KIRC and ICGC cohort.

Supplementary 2 Table S2: immune-related gene sets downloaded from GSEA.

Supplementary 3 Table S3: 280 immune-related DElncRNAs.

Supplementary 4 Figure S1: the expression pattern of the five PIDElncRNAs and the risk score in different AJCC-stage and Fuhrman grade. (A-J) AC012236.1, AC078778.1, AC078950.1, AC087318.1, and AC092535.4. (K-L) The risk score.

Supplementary 5 Figure S2: prognostic analysis of the five-lncRNA signature in ICGC dataset. (A) Kaplan-Meier survival analysis of the five-lncRNA signature. (B) Time-dependent ROC curves of the five-lncRNA signature.

Supplementary 6 Figure S3: Kaplan-Meier survival analysis of other 9 PIDElncRNAs screened after LASSO regression.

Supplementary 7 Figure S4: GSEA of the five lncRNAs. GO enrichment analysis of AC012236.1 (A), AC078778.1 (B), AC078950.1 (C), AC087318.1 (D), and AC092535.4 (E). KEGG enrichment analysis of AC012236.1 (F), AC078778.1 (G), AC078950.1 (H), AC087318.1 (I), and AC092535.4 (J).

Supplementary 8 Figure S5: Pearson correlation analysis of the expression of five lncRNAs and tumor-infiltrating immune cells. The expression of AC012236.1 was positively correlated with the infiltration of regulatory T cells (A) and follicular helper T cell (B) and negatively correlated with that of resting mast cells (C). The expression of AC078778.1 was positively correlated with the infiltration of regulatory T cells (D), follicular helper T cells (E), and CD8 T cells (G) and negatively correlated with that of resting mast cells (F) and naive B cells (H). The expression of AC087318.1 was negatively correlated with the infiltration of naive B cells (I) and resting mast cells (J) and positively correlated with that of CD8 T cells (K) and follicular helper T cells (L). The expression of AC092535.4 was positively correlated with the infiltration of regulatory T cells (M) and negatively correlated with that of mast cell resting (N).

Supplementary 9 Table S4: the coexpression analysis of DETFs and final 5 PIDElncRNAs in TCGA dataset.

Supplementary 10 Table S5: the coexpression analysis of DETFs and final 5 PIDElncRNAs in ICGC dataset.

Supplementary 11 Table S6: the coexpression analysis between the 5 PIDElncRNAs and immune-related mRNA.
