# Supplementary material for: A Novel Immune-Related lncRNA-Based Model for Survival Prediction in Clear Cell Renal Cell Carcinoma
Source: J Immunol Res. 2021 Jun 28;2021:9921466. doi: 10.1155/2021/9921466 (PMC8339875; doi:10.1155/2021/9921466)
Supplement: Supplementary 1 — Table S1: clinical information of TCGA KIRC and ICGC cohort. [file 9921466.f1.docx]

| **Table S1: Clinical information of TCGA and ICGC KIRC cohort** | | | | | | | | |  |  |
| --- | --- | --- | --- | --- | --- | --- | --- | --- | --- | --- |
|  | | TCGA | | | | | | ICGC | | |
|  |  | Training Set (n=309) | | Testing set (n=203) | | Whole set (n=512) | | (n=91) | | |
|  |  |  |  |  |  |  |  |  |  |  |
| Gender | Male | 200 | 64.72% | 136 | 67.00% | 336 | 65.63% | 52 | | 57.14% |
|  | Female | 109 | 35.28% | 67 | 33.00% | 176 | 34.38% | 39 | | 42.86% |
| Age | Younger (≦65y) | 210 | 67.96% | 129 | 63.55% | 339 | 66.21% | 63 | | 69.23% |
|  | Older (﹥65y) | 99 | 32.04% | 74 | 36.45% | 173 | 33.79% | 28 | | 30.77% |
| Race | Asian | 3 | 0.97% | 5 | 2.46% | 8 | 1.56% |  | |  |
|  | Black or African American | 30 | 9.71% | 21 | 10.34% | 51 | 9.96% |  | |  |
|  | White | 276 | 89.32% | 177 | 87.19% | 453 | 88.48% |  | |  |
| T stage | T1 | 152 | 49.19% | 108 | 53.20% | 260 | 50.78% | 53 | | 58.24% |
|  | T2 | 41 | 13.27% | 27 | 13.30% | 68 | 13.28% | 12 | | 13.19% |
|  | T3 | 111 | 35.92% | 62 | 30.54% | 173 | 33.79% | 21 | | 23.08% |
|  | T4 | 5 | 1.62% | 6 | 2.96% | 11 | 2.15% | 2 | | 2.20% |
| N stage | N0 | 134 | 43.37% | 94 | 46.31% | 228 | 44.53% | 79 | | 86.81% |
|  | N1 | 10 | 3.24% | 6 | 2.96% | 16 | 3.13% | 2 | | 2.20% |
|  | Nx | 165 | 53.40% | 103 | 50.74% | 268 | 52.34% | 10 | | 10.99% |
| M stage | M0 | 239 | 77.35% | 167 | 82.27% | 406 | 79.30% | 81 | | 89.01% |
|  | M1 | 53 | 17.15% | 25 | 12.32% | 78 | 15.23% | 9 | | 9.89% |
|  | Mx | 17 | 5.50% | 11 | 5.42% | 28 | 5.47% | 1 | | 1.10% |
| TNM stage | I | 150 | 48.54% | 106 | 52.22% | 256 | 50.00% |  | |  |
|  | II | 31 | 10.03% | 25 | 12.32% | 56 | 10.94% |  | |  |
|  | III | 73 | 23.62% | 45 | 22.17% | 118 | 23.05% |  | |  |
|  | IV | 55 | 17.80% | 27 | 13.30% | 82 | 16.02% |  | |  |
| Grade | 1 | 9 | 2.91% | 2 | 0.99% | 11 | 2.15% |  | |  |
|  | 2 | 126 | 40.78% | 93 | 45.81% | 219 | 42.77% |  | |  |
|  | 3 | 124 | 40.13% | 79 | 38.92% | 203 | 39.65% |  | |  |
|  | 4 | 48 | 15.53% | 25 | 12.32% | 73 | 14.26% |  | |  |
|  | X | 2 | 0.65% | 4 | 1.97% | 6 | 1.17% |  | |  |
| Pharmaceutical | No | 261 | 84.47% | 178 | 87.68% | 439 | 85.74% |  | |  |
|  | Yes | 48 | 15.53% | 25 | 12.32% | 73 | 14.26% |  | |  |
| Radiation | No | 305 | 98.71% | 202 | 99.51% | 507 | 99.02% |  | |  |
|  | Yes | 4 | 1.29% | 1 | 0.49% | 5 | 0.98% |  | |  |
| Total | | 309 | 100.00% | 203 | 100.00% | 512 | 100.00% | 91 | | 100.00% |
