# Supplementary material for: A Novel Immune-Related lncRNA-Based Model for Survival Prediction in Clear Cell Renal Cell Carcinoma
Source: J Immunol Res. 2021 Jun 28;2021:9921466. doi: 10.1155/2021/9921466 (PMC8339875; doi:10.1155/2021/9921466)
Supplement: Supplementary 2 — Table S2: immune-related gene sets downloaded from GSEA. [file 9921466.f2.docx]

| **Table S2: Immune-related gene sets downloaded from GSEA** |
| --- |
| GO_ACTIVATION_OF_IMMUNE_RESPONSE |
| GO_ACTIVATION_OF_INNATE_IMMUNE_RESPONSE |
| GO_ADAPTIVE_IMMUNE_RESPONSE |
| GO_ADAPTIVE_IMMUNE_RESPONSE_BASED_ON_SOMATIC_RECOMBINATION_OF_IMMUNE_  RECEPTORS_BUILT_FROM_IMMUNOGLOBULIN_SUPERFAMILY_DOMAINS |
| GO_B_CELL_ACTIVATION_INVOLVED_IN_IMMUNE_RESPONSE |
| GO_CELL_ACTIVATION_INVOLVED_IN_IMMUNE_RESPONSE |
| GO_CYTOKINE_PRODUCTION_INVOLVED_IN_IMMUNE_RESPONSE |
| GO_CYTOKINE_SECRETION_INVOLVED_IN_IMMUNE_RESPONSE |
| GO_DENDRITIC_CELL_APOPTOTIC_PROCESS |
| GO_DENDRITIC_CELL_DIFFERENTIATION |
| GO_HUMORAL_IMMUNE_RESPONSE |
| GO_IMMUNE_EFFECTOR_PROCESS |
| GO_IMMUNE_RESPONSE_INHIBITING_SIGNAL_TRANSDUCTION |
| GO_IMMUNE_RESPONSE_REGULATING_CELL_SURFACE_RECEPTOR_SIGNALING_PATHWAY |
| GO_IMMUNE_RESPONSE_REGULATING_SIGNALING_PATHWAY |
| GO_IMMUNE_RESPONSE_TO_TUMOR_CELL |
| GO_IMMUNOGLOBULIN_PRODUCTION_INVOLVED_IN_IMMUNOGLOBULIN_MEDIATED_IMMUNE_  RESPONSE |
| GO_IMMUNOLOGICAL_MEMORY_PROCESS |
| GO_INNATE_IMMUNE_RESPONSE |
| GO_LEUKOCYTE_MEDIATED_IMMUNITY |
| GO_LEUKOCYTE_CHEMOTAXIS |
| GO_LYMPHOCYTE_CHEMOTAXIS |
| GO_LYMPHOCYTE_MEDIATED_IMMUNITY |
| GO_MACROPHAGE_CHEMOTAXIS |
| GO_MAST_CELL_MEDIATED_IMMUNITY |
| GO_NATURAL_KILLER_CELL_ACTIVATION |
| GO_NATURAL_KILLER_CELL_MEDIATED_IMMUNITY |
| GO_NEGATIVE_REGULATION_OF_CYTOKINE_PRODUCTION_INVOLVED_IN_IMMUNE_RESPONSE |
| GO_NEGATIVE_REGULATION_OF_CYTOKINE_SECRETION_INVOLVED_IN_IMMUNE_RESPONSE |
| GO_POSITIVE_REGULATION_OF_IMMUNE_RESPONSE |
| GO_POSITIVE_REGULATION_OF_IMMUNE_EFFECTOR_PROCESS |
| GO_POSITIVE_REGULATION_OF_IMMUNE_SYSTEM_PROCESS |
| GO_REGULATION_OF_ADAPTIVE_IMMUNE_RESPONSE |
| GO_REGULATION_OF_HUMORAL_IMMUNE_RESPONSE |
| GO_REGULATION_OF_IMMUNE_EFFECTOR_PROCESS |
| GO_REGULATION_OF_IMMUNE_RESPONSE |
| GO_REGULATION_OF_IMMUNE_SYSTEM_PROCESS |
| GO_REGULATION_OF_INNATE_IMMUNE_RESPONSE |
| GO_REGULATION_OF_RESPONSE_TO_TUMOR_CELL |
| GO_REGULATORY_T_CELL_DIFFERENTIATION |
| GO_T_CELL_CHEMOTAXIS |
| GO_T_CELL_ACTIVATION_INVOLVED_IN_IMMUNE_RESPONSE |
| **Immune-related gene removing duplicates** |
| A2M |
| ABI1 |
| ABL1 |
| ACOD1 |
| ACTB |
| ACTG1 |
| ACTR2 |
| ACTR3 |
| ADA |
| AIM2 |
| ALPK1 |
| ANKRD17 |
| APCS |
| APOB |
| APPL1 |
| APPL2 |
| ARPC1A |
| ARPC1B |
| ARPC2 |
| ARPC3 |
| ARPC4 |
| ARPC5 |
| ARRB2 |
| BAG6 |
| BAIAP2 |
| BAX |
| BCAR1 |
| BCL10 |
| BCL2 |
| BIRC2 |
| BIRC3 |
| BLK |
| BMX |
| BPIFB1 |
| BRAF |
| BRK1 |
| BTK |
| BTN1A1 |
| BTN2A1 |
| BTN2A2 |
| BTN2A3P |
| BTN3A1 |
| BTN3A2 |
| BTN3A3 |
| BTNL10 |
| BTNL2 |
| BTNL3 |
| BTNL8 |
| BTNL9 |
| BTRC |
| C1QA |
| C1QB |
| C1QBP |
| C1QC |
| C1R |
| C1RL |
| C1S |
| C2 |
| C3 |
| C3AR1 |
| C4A |
| C4B |
| C4BPA |
| C4BPB |
| C5 |
| C5AR1 |
| C5AR2 |
| C6 |
| C7 |
| C8A |
| C8B |
| C8G |
| C9 |
| CACNA1F |
| CACNB3 |
| CACTIN |
| CARD11 |
| CARD9 |
| CASP8 |
| CAV1 |
| CBFB |
| CCR7 |
| CD14 |
| CD160 |
| CD19 |
| CD209 |
| CD22 |
| CD226 |
| CD247 |
| CD276 |
| CD28 |
| CD300A |
| CD300LF |
| CD36 |
| CD38 |
| CD3D |
| CD3E |
| CD3G |
| CD4 |
| CD46 |
| CD47 |
| CD55 |
| CD59 |
| CD79A |
| CD79B |
| CD81 |
| CDC42 |
| CEACAM1 |
| CFB |
| CFD |
| CFH |
| CFHR1 |
| CFHR2 |
| CFHR4 |
| CFHR5 |
| CFI |
| CFP |
| CGAS |
| CHUK |
| CLEC10A |
| CLEC4A |
| CLEC4C |
| CLEC4D |
| CLEC4E |
| CLEC6A |
| CLEC7A |
| CLU |
| CMKLR1 |
| CMTM3 |
| CNPY3 |
| COL20A1 |
| COLEC10 |
| COLEC11 |
| COLEC12 |
| CPB2 |
| CPN1 |
| CPN2 |
| CR1 |
| CR2 |
| CREBBP |
| CRK |
| CRKL |
| CRP |
| CSK |
| CTLA4 |
| CTSB |
| CTSK |
| CTSL |
| CTSS |
| CUL1 |
| CYBA |
| CYFIP1 |
| CYFIP2 |
| CYLD |
| DAB2IP |
| DDX58 |
| DDX60 |
| DENND1B |
| DHX58 |
| DMBT1 |
| DOCK1 |
| DUSP22 |
| DUSP3 |
| EIF2B1 |
| EIF2B2 |
| EIF2B3 |
| EIF2B4 |
| EIF2B5 |
| ELF1 |
| ELF2 |
| ELMO1 |
| ELMO2 |
| EP300 |
| ERBIN |
| ERMAP |
| ESR1 |
| EZR |
| F2 |
| F2RL1 |
| FADD |
| FBXW11 |
| FCER1G |
| FCGR1A |
| FCGR2A |
| FCGR2B |
| FCGR3A |
| FCN1 |
| FCN2 |
| FCN3 |
| FCRL3 |
| FFAR2 |
| FGA |
| FGB |
| FGG |
| FGR |
| FLOT1 |
| FOXP1 |
| FOXP3 |
| FPR1 |
| FPR2 |
| FPR3 |
| FYB1 |
| FYB2 |
| FYN |
| GATA3 |
| GBP1 |
| GCSAM |
| GCSAML |
| GFI1 |
| GPATCH3 |
| GPLD1 |
| GPR32 |
| GPR32P1 |
| GPR33 |
| GPS2 |
| GRAMD4 |
| GRAP2 |
| GRB2 |
| HAVCR2 |
| HCK |
| HEXIM1 |
| HHLA2 |
| HLA-DPA1 |
| HLA-DPB1 |
| HLA-DQA1 |
| HLA-DQA2 |
| HLA-DQB1 |
| HLA-DQB2 |
| HLA-DRA |
| HLA-DRB1 |
| HLA-DRB3 |
| HLA-DRB4 |
| HLA-DRB5 |
| HMGB1 |
| HMGB4 |
| HMSD |
| HRAS |
| HSP90AA1 |
| HSP90AB1 |
| HSP90B1 |
| HSPA1A |
| HSPA1B |
| HSPD1 |
| ICAM2 |
| ICAM3 |
| ICOSLG |
| IFI16 |
| IFIH1 |
| IGHA1 |
| IGHA2 |
| IGHD |
| IGHE |
| IGHG1 |
| IGHG2 |
| IGHG3 |
| IGHG4 |
| IGHM |
| IGHV1-18 |
| IGHV1-24 |
| IGHV1-3 |
| IGHV1-45 |
| IGHV1-58 |
| IGHV1-69 |
| IGHV1-69-2 |
| IGHV1-69D |
| IGHV1OR15-1 |
| IGHV2-26 |
| IGHV2-5 |
| IGHV2-70 |
| IGHV2-70D |
| IGHV3-11 |
| IGHV3-13 |
| IGHV3-15 |
| IGHV3-20 |
| IGHV3-21 |
| IGHV3-23 |
| IGHV3-30 |
| IGHV3-33 |
| IGHV3-43 |
| IGHV3-48 |
| IGHV3-49 |
| IGHV3-53 |
| IGHV3-64 |
| IGHV3-64D |
| IGHV3-66 |
| IGHV3-7 |
| IGHV3-72 |
| IGHV3-73 |
| IGHV3-74 |
| IGHV4-28 |
| IGHV4-31 |
| IGHV4-34 |
| IGHV4-39 |
| IGHV4-4 |
| IGHV4-59 |
| IGHV4-61 |
| IGHV5-10-1 |
| IGHV5-51 |
| IGHV6-1 |
| IGHV7-4-1 |
| IGKC |
| IGKV1-12 |
| IGKV1-16 |
| IGKV1-17 |
| IGKV1-39 |
| IGKV1-5 |
| IGKV1D-12 |
| IGKV1D-33 |
| IGKV1D-39 |
| IGKV2-28 |
| IGKV2-29 |
| IGKV2-30 |
| IGKV2-40 |
| IGKV2D-28 |
| IGKV2D-30 |
| IGKV3-15 |
| IGKV3-20 |
| IGKV3D-11 |
| IGKV3D-20 |
| IGKV4-1 |
| IGKV5-2 |
| IGLC1 |
| IGLC2 |
| IGLC3 |
| IGLC6 |
| IGLC7 |
| IGLL1 |
| IGLL5 |
| IGLV1-40 |
| IGLV1-44 |
| IGLV1-47 |
| IGLV1-51 |
| IGLV2-11 |
| IGLV2-14 |
| IGLV2-23 |
| IGLV2-8 |
| IGLV3-1 |
| IGLV3-19 |
| IGLV3-21 |
| IGLV3-25 |
| IGLV3-27 |
| IGLV6-57 |
| IGLV7-43 |
| IKBKB |
| IKBKE |
| IKBKG |
| INAVA |
| INPP5D |
| IRAK1 |
| IRAK2 |
| IRAK3 |
| IRAK4 |
| IRF1 |
| IRF3 |
| IRF4 |
| IRF7 |
| IRGM |
| ITCH |
| ITGAM |
| ITGB2 |
| ITK |
| KCNN4 |
| KLHL6 |
| KLRC4-KLRK1 |
| KLRK1 |
| KRAS |
| KRT1 |
| LAT |
| LAT2 |
| LBP |
| LCK |
| LCP2 |
| LGALS3 |
| LGMN |
| LGR4 |
| LILRA2 |
| LILRA4 |
| LILRB4 |
| LIME1 |
| LIMK1 |
| LPXN |
| LRRC14 |
| LSM14A |
| LTF |
| LY96 |
| LYN |
| MALT1 |
| MAP2K6 |
| MAP3K1 |
| MAP3K7 |
| MAPK1 |
| MAPK3 |
| MAPKAPK2 |
| MAPKAPK3 |
| MARCO |
| MASP1 |
| MASP2 |
| MATR3 |
| MAVS |
| MBL2 |
| MEF2C |
| MFHAS1 |
| MICB |
| MIR146A |
| MIR146B |
| MIR17 |
| MIR18A |
| MIR19A |
| MIR34A |
| MIR520B |
| MIR520E |
| MNDA |
| MOG |
| MUC1 |
| MUC12 |
| MUC13 |
| MUC15 |
| MUC16 |
| MUC17 |
| MUC19 |
| MUC2 |
| MUC20 |
| MUC21 |
| MUC3A |
| MUC4 |
| MUC5AC |
| MUC5B |
| MUC6 |
| MUC7 |
| MUCL1 |
| MYD88 |
| MYH2 |
| MYO10 |
| MYO1C |
| MYO1G |
| NCK1 |
| NCKAP1 |
| NCKAP1L |
| NCKIPSD |
| NCR3 |
| NECTIN2 |
| NFAM1 |
| NFATC2 |
| NFKB1 |
| NFKBIA |
| NFKBID |
| NFKBIL1 |
| NFKBIZ |
| NLRC4 |
| NLRP2B |
| NLRP6 |
| NLRX1 |
| NOD1 |
| NOD2 |
| NONO |
| NOP53 |
| NPLOC4 |
| NR1D1 |
| NR1H3 |
| NR1H4 |
| NR4A3 |
| NRAS |
| OTUD4 |
| OTULIN |
| PAG1 |
| PAK1 |
| PAK2 |
| PAK3 |
| PAWR |
| PAX5 |
| PDE4B |
| PDE4D |
| PDPK1 |
| PELI1 |
| PGLYRP1 |
| PGLYRP2 |
| PGLYRP3 |
| PGLYRP4 |
| PHB |
| PHPT1 |
| PIK3AP1 |
| PIK3C3 |
| PIK3CA |
| PIK3CB |
| PIK3CD |
| PIK3R1 |
| PIK3R2 |
| PIK3R4 |
| PJA2 |
| PLA2G6 |
| PLCG1 |
| PLCG2 |
| PLCL2 |
| PLD2 |
| PLEKHA1 |
| PLPP4 |
| PLSCR1 |
| PQBP1 |
| PRAM1 |
| PRKACA |
| PRKACB |
| PRKACG |
| PRKCB |
| PRKCD |
| PRKCE |
| PRKCH |
| PRKCQ |
| PRKD2 |
| PRKDC |
| PRNP |
| PROS1 |
| PSEN1 |
| PSMA1 |
| PSMA2 |
| PSMA3 |
| PSMA4 |
| PSMA5 |
| PSMA6 |
| PSMA7 |
| PSMA8 |
| PSMB1 |
| PSMB10 |
| PSMB11 |
| PSMB2 |
| PSMB3 |
| PSMB4 |
| PSMB5 |
| PSMB6 |
| PSMB7 |
| PSMB8 |
| PSMB9 |
| PSMC1 |
| PSMC2 |
| PSMC3 |
| PSMC4 |
| PSMC5 |
| PSMC6 |
| PSMD1 |
| PSMD10 |
| PSMD11 |
| PSMD12 |
| PSMD13 |
| PSMD14 |
| PSMD2 |
| PSMD3 |
| PSMD4 |
| PSMD5 |
| PSMD6 |
| PSMD7 |
| PSMD8 |
| PSMD9 |
| PSME1 |
| PSME2 |
| PSME3 |
| PSME4 |
| PSMF1 |
| PSPC1 |
| PTK2 |
| PTPN2 |
| PTPN22 |
| PTPN6 |
| PTPRC |
| PTPRJ |
| PTPRS |
| PUM1 |
| PUM2 |
| PVRIG |
| PYCARD |
| PYHIN1 |
| RAB29 |
| RAB7B |
| RAC1 |
| RAF1 |
| RAP1A |
| RAPGEF1 |
| RBCK1 |
| RBM14 |
| RC3H1 |
| RC3H2 |
| REG3G |
| RELA |
| RELB |
| RFTN1 |
| RGCC |
| RIOK3 |
| RIPK1 |
| RIPK2 |
| RNF125 |
| RNF31 |
| RPS27A |
| RPS3 |
| RPS6KA3 |
| RPS6KA5 |
| RSAD2 |
| RTN4 |
| RUNX1 |
| S100A1 |
| S100A14 |
| S100A8 |
| S100A9 |
| SARM1 |
| SCARA3 |
| SEC14L1 |
| SERPING1 |
| SFPQ |
| SFTPA1 |
| SFTPA2 |
| SFTPD |
| SH2B2 |
| SIN3A |
| SKAP1 |
| SKP1 |
| SLA2 |
| SLC39A10 |
| SMPDL3B |
| SPG21 |
| SPPL3 |
| SRC |
| STAP1 |
| STK11 |
| STOML2 |
| SUSD4 |
| SYK |
| TAB1 |
| TAB2 |
| TAB3 |
| TANK |
| TBK1 |
| TEC |
| TESPA1 |
| THEMIS |
| THEMIS2 |
| THY1 |
| TICAM1 |
| TICAM2 |
| TIFA |
| TIRAP |
| TKFC |
| TLR1 |
| TLR10 |
| TLR2 |
| TLR3 |
| TLR4 |
| TLR5 |
| TLR6 |
| TLR7 |
| TLR8 |
| TLR9 |
| TMEM173 |
| TNFAIP3 |
| TNFRSF21 |
| TNIP1 |
| TNIP2 |
| TNIP3 |
| TRAC |
| TRAF3 |
| TRAF6 |
| TRAT1 |
| TRAV19 |
| TRAV29DV5 |
| TRAV8-4 |
| TRBC1 |
| TRBC2 |
| TRBV12-3 |
| TRBV7-9 |
| TRDC |
| TREML4 |
| TREX1 |
| TRIL |
| TRIM15 |
| TRIM5 |
| TSPAN6 |
| TXK |
| TYRO3 |
| UBA52 |
| UBASH3A |
| UBB |
| UBC |
| UBE2D1 |
| UBE2D2 |
| UBE2D3 |
| UBE2N |
| UBE2V1 |
| UBQLN1 |
| UFD1 |
| UNC93B1 |
| USP17L2 |
| VAV1 |
| VAV2 |
| VAV3 |
| VSIG4 |
| VTCN1 |
| VTN |
| WAS |
| WASF2 |
| WASL |
| WDFY1 |
| WIPF1 |
| WIPF2 |
| WIPF3 |
| WNK1 |
| XIAP |
| XRCC5 |
| XRCC6 |
| YES1 |
| ZAP70 |
| ZC3H12A |
| ZCCHC3 |
| ADAM17 |
| ADCY7 |
| ADGRE1 |
| AGER |
| AICDA |
| AIRE |
| ALCAM |
| ALOX15 |
| ANXA1 |
| APLF |
| ARG1 |
| ARG2 |
| ATAD5 |
| B2M |
| BATF |
| BCL3 |
| BCL6 |
| BTLA |
| C17orf99 |
| CAMK4 |
| CCL19 |
| CCR2 |
| CCR6 |
| CD1A |
| CD1B |
| CD1C |
| CD1D |
| CD1E |
| CD244 |
| CD27 |
| CD274 |
| CD40 |
| CD40LG |
| CD48 |
| CD6 |
| CD7 |
| CD74 |
| CD80 |
| CD84 |
| CD86 |
| CD8A |
| CD8B |
| CD8B2 |
| CLC |
| CLCF1 |
| CLEC4G |
| CLEC4M |
| CRACR2A |
| CTSC |
| CTSH |
| CXCL13 |
| DBNL |
| DCLRE1C |
| DLG1 |
| DUSP10 |
| EBI3 |
| EIF2AK4 |
| EMP2 |
| EOMES |
| ERAP1 |
| ERAP2 |
| ERCC1 |
| EXO1 |
| EXOSC3 |
| EXOSC6 |
| FAM49B |
| FCAMR |
| FCER2 |
| FCGR1B |
| FCRL4 |
| FOXJ1 |
| FZD5 |
| GAPT |
| GCNT3 |
| GNL1 |
| GPR183 |
| GZMM |
| HFE |
| HLA-A |
| HLA-B |
| HLA-C |
| HLA-E |
| HLA-F |
| HLA-G |
| HLA-H |
| HLX |
| HMHB1 |
| HPRT1 |
| HPX |
| ICAM1 |
| IFNA1 |
| IFNA10 |
| IFNA13 |
| IFNA14 |
| IFNA16 |
| IFNA17 |
| IFNA2 |
| IFNA21 |
| IFNA4 |
| IFNA5 |
| IFNA6 |
| IFNA7 |
| IFNA8 |
| IFNB1 |
| IFNE |
| IFNG |
| IFNK |
| IFNW1 |
| IGHD1-1 |
| IGHJ1 |
| IGKJ1 |
| IGKV1-13 |
| IGKV1-27 |
| IGKV1-6 |
| IGKV1-8 |
| IGKV1-9 |
| IGKV1D-13 |
| IGKV1D-17 |
| IGKV1D-43 |
| IGKV1D-8 |
| IGKV2-24 |
| IGKV2D-26 |
| IGKV2D-29 |
| IGKV3D-15 |
| IGKV3D-7 |
| IGKV6-21 |
| IGKV6D-21 |
| IGLJ1 |
| IGLV1-36 |
| IGLV10-54 |
| IGLV2-18 |
| IGLV3-10 |
| IGLV3-12 |
| IGLV3-16 |
| IGLV3-22 |
| IGLV3-9 |
| IGLV4-3 |
| IGLV4-60 |
| IGLV4-69 |
| IGLV5-37 |
| IGLV5-45 |
| IGLV5-52 |
| IGLV7-46 |
| IGLV8-61 |
| IGLV9-49 |
| IL10 |
| IL12A |
| IL12B |
| IL12RB1 |
| IL13RA2 |
| IL18 |
| IL18BP |
| IL18R1 |
| IL18RAP |
| IL1B |
| IL1R1 |
| IL1RL1 |
| IL2 |
| IL20RB |
| IL23A |
| IL23R |
| IL27 |
| IL27RA |
| IL31RA |
| IL33 |
| IL4 |
| IL4R |
| IL6 |
| IL6R |
| IL6ST |
| IL7R |
| JAG1 |
| JAK2 |
| JAK3 |
| JAM3 |
| JCHAIN |
| KDELR1 |
| LAIR1 |
| LAMP3 |
| LAX1 |
| LEF1 |
| LIG4 |
| LILRA1 |
| LILRA3 |
| LILRA6 |
| LILRB1 |
| LILRB2 |
| LILRB3 |
| LILRB5 |
| LOXL3 |
| LTA |
| LY9 |
| MAD2L2 |
| 8-Mar |
| MCOLN1 |
| MCOLN2 |
| MICA |
| MIR21 |
| MLH1 |
| MSH2 |
| MSH6 |
| MTOR |
| NBN |
| NDFIP1 |
| NEDD4 |
| NFKB2 |
| NLRP10 |
| NLRP3 |
| NSD2 |
| ORAI1 |
| OTUB1 |
| OTUD7B |
| P2RX7 |
| PARP3 |
| PAXIP1 |
| PIK3CG |
| PKN1 |
| POU2F2 |
| PPP3CB |
| PRDM1 |
| PRF1 |
| PRKCZ |
| PRR7 |
| PTK2B |
| PVR |
| RAB27A |
| RAET1E |
| RAET1G |
| RAET1L |
| RAG1 |
| RIF1 |
| RIPK3 |
| RNF168 |
| RNF19B |
| RNF8 |
| RORA |
| RORC |
| SAMSN1 |
| SASH3 |
| SCART1 |
| SEMA4A |
| SH2D1A |
| SH2D1B |
| SHLD1 |
| SHLD2 |
| SHLD3 |
| SIGLEC10 |
| SIRT1 |
| SIT1 |
| SLAMF1 |
| SLAMF6 |
| SLAMF7 |
| SLC11A1 |
| SMAD7 |
| SOCS5 |
| SPN |
| STAT3 |
| STAT6 |
| STX7 |
| SUPT6H |
| SWAP70 |
| TAP1 |
| TAP2 |
| TARM1 |
| TBX21 |
| TFE3 |
| TFEB |
| TFRC |
| TGFB1 |
| THOC1 |
| TNF |
| TNFRSF11A |
| TNFRSF13B |
| TNFRSF13C |
| TNFRSF14 |
| TNFRSF17 |
| TNFRSF1B |
| TNFSF13 |
| TNFSF13B |
| TNFSF18 |
| TNFSF4 |
| TP53BP1 |
| TRAF2 |
| TRAJ3 |
| TRAV1-1 |
| TRAV1-2 |
| TRAV10 |
| TRAV12-1 |
| TRAV12-2 |
| TRAV12-3 |
| TRAV13-1 |
| TRAV13-2 |
| TRAV14DV4 |
| TRAV16 |
| TRAV17 |
| TRAV18 |
| TRAV2 |
| TRAV20 |
| TRAV21 |
| TRAV22 |
| TRAV23DV6 |
| TRAV24 |
| TRAV25 |
| TRAV26-1 |
| TRAV26-2 |
| TRAV27 |
| TRAV3 |
| TRAV30 |
| TRAV34 |
| TRAV35 |
| TRAV36DV7 |
| TRAV38-1 |
| TRAV38-2DV8 |
| TRAV39 |
| TRAV4 |
| TRAV40 |
| TRAV41 |
| TRAV5 |
| TRAV6 |
| TRAV7 |
| TRAV8-1 |
| TRAV8-2 |
| TRAV8-3 |
| TRAV8-6 |
| TRAV9-1 |
| TRAV9-2 |
| TRBD1 |
| TRBJ1-1 |
| TRBV10-1 |
| TRBV10-2 |
| TRBV10-3 |
| TRBV11-2 |
| TRBV11-3 |
| TRBV12-4 |
| TRBV12-5 |
| TRBV13 |
| TRBV14 |
| TRBV16 |
| TRBV18 |
| TRBV19 |
| TRBV2 |
| TRBV20-1 |
| TRBV24-1 |
| TRBV25-1 |
| TRBV27 |
| TRBV28 |
| TRBV29-1 |
| TRBV3-1 |
| TRBV30 |
| TRBV4-1 |
| TRBV4-2 |
| TRBV5-1 |
| TRBV5-4 |
| TRBV5-5 |
| TRBV5-6 |
| TRBV6-1 |
| TRBV6-4 |
| TRBV6-5 |
| TRBV6-6 |
| TRBV6-8 |
| TRBV7-2 |
| TRBV7-4 |
| TRBV7-6 |
| TRBV7-7 |
| TRBV9 |
| TRDD1 |
| TRDJ1 |
| TRGC2 |
| TRGJ2 |
| TRIM27 |
| TRPM4 |
| TSC1 |
| TSTA3 |
| ULBP1 |
| ULBP2 |
| ULBP3 |
| UNC13D |
| UNG |
| XCL1 |
| ZBTB1 |
| ZBTB7B |
| ZNF683 |
| ZP3 |
| CD180 |
| CDH17 |
| DLL1 |
| DOCK10 |
| DOCK11 |
| ITFG2 |
| ITM2A |
| LFNG |
| LGALS1 |
| MFNG |
| NKX2-3 |
| NOTCH2 |
| XBP1 |
| A1BG |
| ABCA13 |
| ABR |
| AC004151.1 |
| AC034102.1 |
| ACAA1 |
| ACLY |
| ACPP |
| ACTR10 |
| ACTR1B |
| ADA2 |
| ADAM10 |
| ADAM8 |
| ADGRE2 |
| ADGRE3 |
| ADGRE5 |
| ADGRG3 |
| ADORA2B |
| AGA |
| AGL |
| AGPAT2 |
| AHSG |
| ALAD |
| ALDH3B1 |
| ALDOA |
| ALDOC |
| ALOX5 |
| AMPD3 |
| ANO6 |
| ANPEP |
| ANXA2 |
| ANXA3 |
| AOC1 |
| AP1G1 |
| AP1M1 |
| AP2A2 |
| APAF1 |
| APBB1IP |
| APEH |
| APP |
| APRT |
| ARHGAP9 |
| ARL8A |
| ARMC8 |
| ARSA |
| ARSB |
| ASAH1 |
| ATAD3B |
| ATG7 |
| ATP11A |
| ATP11B |
| ATP6AP2 |
| ATP6V0A1 |
| ATP6V0C |
| ATP6V1D |
| ATP7A |
| ATP8A1 |
| ATP8B4 |
| AZU1 |
| B4GALT1 |
| BCR |
| BIN2 |
| BPI |
| BRI3 |
| BST1 |
| BST2 |
| C12orf4 |
| C1orf35 |
| C6orf120 |
| CAB39 |
| CALML5 |
| CAMP |
| CAND1 |
| CANT1 |
| CAP1 |
| CAPN1 |
| CAT |
| CBL |
| CCL3 |
| CCT2 |
| CCT8 |
| CD177 |
| CD33 |
| CD44 |
| CD53 |
| CD58 |
| CD63 |
| CD68 |
| CD93 |
| CDA |
| CDK13 |
| CEACAM3 |
| CEACAM6 |
| CEACAM8 |
| CEP290 |
| CHGA |
| CHI3L1 |
| CHIT1 |
| CHRNB4 |
| CKAP4 |
| CLEC12A |
| CLEC5A |
| CMTM6 |
| CNN2 |
| COMMD3 |
| COMMD9 |
| COPB1 |
| CORO1A |
| COTL1 |
| CPLX2 |
| CPNE1 |
| CPNE3 |
| CPPED1 |
| CREG1 |
| CRHR1 |
| CRISP3 |
| CRISPLD2 |
| CSNK2B |
| CST3 |
| CSTB |
| CTSA |
| CTSD |
| CTSG |
| CTSZ |
| CXCL1 |
| CXCR1 |
| CXCR2 |
| CYB5R3 |
| CYBB |
| CYSTM1 |
| DDOST |
| DDX3X |
| DEFA1 |
| DEFA1B |
| DEFA4 |
| DEGS1 |
| DERA |
| DGAT1 |
| DIAPH1 |
| DNAJC13 |
| DNAJC3 |
| DNAJC5 |
| DNASE1 |
| DNASE1L1 |
| DNASE1L3 |
| DOCK2 |
| DOK3 |
| DPP7 |
| DSC1 |
| DSG1 |
| DSN1 |
| DSP |
| DYNC1H1 |
| DYNC1LI1 |
| DYNLL1 |
| DYNLT1 |
| DYSF |
| EEF1A1 |
| EEF2 |
| ELANE |
| ENPP3 |
| ENPP4 |
| EPX |
| ERP44 |
| FABP5 |
| FAF2 |
| FCAR |
| FCGR3B |
| FER |
| FES |
| FGL2 |
| FLG2 |
| FOLR3 |
| FOXF1 |
| FRK |
| FRMPD3 |
| FTH1 |
| FTL |
| FUCA1 |
| FUCA2 |
| GAA |
| GAB2 |
| GALNS |
| GATA2 |
| GBF1 |
| GCA |
| GDI2 |
| GGH |
| GHDC |
| GLA |
| GLB1 |
| GLIPR1 |
| GM2A |
| GMFG |
| GNS |
| GOLGA7 |
| GPI |
| GPR84 |
| GRN |
| GSDMD |
| GSN |
| GSTP1 |
| GUSB |
| GYG1 |
| HBB |
| HEBP2 |
| HEXB |
| HGSNAT |
| HK3 |
| HLA-DMB |
| HMOX1 |
| HMOX2 |
| HP |
| HPSE |
| HRNR |
| HSPA6 |
| HSPA8 |
| HUWE1 |
| HVCN1 |
| IDH1 |
| IFNL1 |
| IGF2R |
| IL13 |
| ILF2 |
| IMPDH1 |
| IMPDH2 |
| IQGAP1 |
| IQGAP2 |
| IST1 |
| ITGAL |
| ITGAV |
| ITGAX |
| JUP |
| KARS |
| KCMF1 |
| KCNAB2 |
| KIT |
| KLRF2 |
| KPNB1 |
| LAMP1 |
| LAMP2 |
| LAMTOR1 |
| LAMTOR2 |
| LAMTOR3 |
| LCN2 |
| LCP1 |
| LGALS9 |
| LPCAT1 |
| LRG1 |
| LRMP |
| LRP1 |
| LRRC7 |
| LTA4H |
| LYZ |
| MAGT1 |
| MAN2B1 |
| MANBA |
| MAPK14 |
| MCEMP1 |
| METTL7A |
| MGAM |
| MGST1 |
| MIF |
| MILR1 |
| MLEC |
| MME |
| MMP25 |
| MMP8 |
| MMP9 |
| MOSPD2 |
| MPO |
| MRGPRX2 |
| MS4A3 |
| MVP |
| MYB |
| NAPRT |
| NBEAL2 |
| NCSTN |
| NDUFC2 |
| NEU1 |
| NFASC |
| NHLRC3 |
| NIT2 |
| NME2 |
| NPC2 |
| NPPA |
| OLFM4 |
| OLR1 |
| ORM1 |
| ORM2 |
| ORMDL3 |
| OSCAR |
| OSTF1 |
| P2RX1 |
| PA2G4 |
| PADI2 |
| PAFAH1B2 |
| PDAP1 |
| PDXK |
| PECAM1 |
| PFKL |
| PGAM1 |
| PGM1 |
| PGM2 |
| PGRMC1 |
| PI4K2A |
| PIGR |
| PKM |
| PKP1 |
| PLA2G3 |
| PLAC8 |
| PLAU |
| PLAUR |
| PLD1 |
| PLEKHO2 |
| PNP |
| PPBP |
| PPIA |
| PPIE |
| PRCP |
| PRDX4 |
| PRDX6 |
| PRG2 |
| PRG3 |
| PRSS2 |
| PRSS3 |
| PRTN3 |
| PSAP |
| PTAFR |
| PTGER4 |
| PTGES2 |
| PTPRB |
| PTPRN2 |
| PTX3 |
| PYGB |
| PYGL |
| QPCT |
| QSOX1 |
| RAB10 |
| RAB14 |
| RAB18 |
| RAB24 |
| RAB31 |
| RAB37 |
| RAB3A |
| RAB3D |
| RAB44 |
| RAB4B |
| RAB5C |
| RAB6A |
| RAB7A |
| RAB9B |
| RAC2 |
| RAP1B |
| RAP2B |
| RAP2C |
| RARA |
| RASGRP1 |
| RETN |
| RHOA |
| RHOF |
| RHOG |
| RNASE2 |
| RNASE3 |
| RNASET2 |
| ROCK1 |
| S100A11 |
| S100A12 |
| S100A13 |
| S100A7 |
| S100P |
| SBNO2 |
| SCAMP1 |
| SDCBP |
| SELL |
| SERPINA1 |
| SERPINA3 |
| SERPINB1 |
| SERPINB10 |
| SERPINB12 |
| SERPINB3 |
| SERPINB6 |
| SIGLEC14 |
| SIGLEC5 |
| SIGLEC9 |
| SIRPA |
| SIRPB1 |
| SLC15A4 |
| SLC27A2 |
| SLC2A3 |
| SLC2A5 |
| SLC44A2 |
| SLCO4C1 |
| SLPI |
| SNAP23 |
| SNAP25 |
| SNAP29 |
| SNX4 |
| SPTAN1 |
| SRP14 |
| STBD1 |
| STK10 |
| STK11IP |
| STOM |
| STX4 |
| STXBP1 |
| STXBP2 |
| STXBP3 |
| SUCNR1 |
| SURF4 |
| SVIP |
| SYNGR1 |
| TBC1D10C |
| TCIM |
| TCIRG1 |
| TCN1 |
| TIMP2 |
| TMBIM1 |
| TMC6 |
| TMEM179B |
| TMEM30A |
| TMEM63A |
| TNFAIP6 |
| TOLLIP |
| TOM1 |
| TRAPPC1 |
| TRPM2 |
| TSPAN14 |
| TTR |
| TUBB |
| TUBB4B |
| TXNDC5 |
| TYROBP |
| UBR4 |
| VAMP2 |
| VAMP7 |
| VAMP8 |
| VAPA |
| VAT1 |
| VCL |
| VCP |
| VNN1 |
| VPS35L |
| YPEL5 |
| ZFPM1 |
| ANGPT1 |
| APOA1 |
| APOA2 |
| ATG5 |
| CD96 |
| CUEDC2 |
| DDX1 |
| DDX21 |
| DHX36 |
| FFAR3 |
| GAS6 |
| GPRC5B |
| HK1 |
| KIR2DL4 |
| MIR155 |
| MR1 |
| SEMA7A |
| SPON2 |
| TGFB2 |
| TGFB3 |
| TRIM6 |
| WNT5A |
| AXL |
| CCL21 |
| CXCL12 |
| RAPGEF2 |
| AC005840.1 |
| AZI2 |
| BATF2 |
| BATF3 |
| CEBPB |
| CSF2 |
| DCSTAMP |
| DHRS2 |
| FLT3 |
| GATA1 |
| MIR223 |
| RBPJ |
| SPI1 |
| TGFBR2 |
| TMEM176A |
| TMEM176B |
| TREM2 |
| UBD |
| ZBTB46 |
| AC118754.1 |
| BLNK |
| BPIFA1 |
| BPIFA2 |
| BPIFB2 |
| CCL13 |
| CCL2 |
| CD83 |
| CST9 |
| CXCL10 |
| CXCL11 |
| CXCL2 |
| CXCL3 |
| CXCL5 |
| CXCL6 |
| CXCL8 |
| CXCL9 |
| DCD |
| DEFA3 |
| DEFA5 |
| DEFA6 |
| DEFB1 |
| DEFB103A |
| DEFB103B |
| DEFB118 |
| DEFB126 |
| DEFB127 |
| DEFB4A |
| EPPIN |
| FAM3A |
| GALP |
| GAPDH |
| GNLY |
| H2BFS |
| HIST1H2BC |
| HIST1H2BE |
| HIST1H2BF |
| HIST1H2BG |
| HIST1H2BI |
| HIST1H2BJ |
| HIST1H2BK |
| HIST2H2BE |
| HMGN2 |
| HRG |
| HTN1 |
| HTN3 |
| IL36RN |
| IL7 |
| ITLN1 |
| KLK3 |
| KLK5 |
| KLK7 |
| KRT6A |
| LEAP2 |
| MNX1 |
| MS4A1 |
| NOTCH1 |
| PDCD1 |
| PDZD11 |
| PF4 |
| PF4V1 |
| PGC |
| PI3 |
| PLA2G1B |
| PLA2G2A |
| POU2AF1 |
| PPP2R3C |
| RARRES2 |
| REG1A |
| REG1B |
| REG3A |
| RNASE6 |
| RNASE7 |
| ROMO1 |
| RPL30 |
| RPL39 |
| RPS19 |
| SEMG1 |
| SEMG2 |
| SPAG11A |
| SPAG11B |
| SPINK5 |
| ST6GAL1 |
| TRAF3IP2 |
| TREM1 |
| YTHDF2 |
| ZP4 |
| ABCC9 |
| ABCE1 |
| ABCF3 |
| AC004551.1 |
| ACE |
| ADAR |
| ADARB1 |
| AGBL4 |
| AGBL5 |
| AIMP1 |
| APOBEC1 |
| APOBEC3A |
| APOBEC3B |
| APOBEC3C |
| APOBEC3D |
| APOBEC3F |
| APOBEC3G |
| APOBEC3H |
| BECN1 |
| BNIP3 |
| BNIP3L |
| C19orf66 |
| CADM1 |
| CD207 |
| CEBPG |
| CLEC12B |
| CLEC2A |
| CNOT7 |
| CRCP |
| CRTAM |
| CYBC1 |
| DBH |
| DDIT4 |
| DDX17 |
| DDX41 |
| DHX9 |
| DTX3L |
| EIF2AK2 |
| ELMOD2 |
| ERCC6 |
| EXOC1 |
| EXOSC4 |
| EXOSC5 |
| FAM111A |
| FLNA |
| FUT7 |
| GBP3 |
| GPAM |
| GZMB |
| HERC5 |
| HTRA1 |
| HYAL2 |
| IFI27 |
| IFI44L |
| IFI6 |
| IFIT1 |
| IFIT1B |
| IFIT2 |
| IFIT3 |
| IFIT5 |
| IFITM1 |
| IFITM2 |
| IFITM3 |
| IFNAR2 |
| IFNL2 |
| IFNL3 |
| IFNL4 |
| IFNLR1 |
| IL10RB |
| IL15 |
| IL21 |
| IL2RA |
| IL5 |
| ILF3 |
| INS |
| IRF2 |
| IRF5 |
| IRF9 |
| ISG15 |
| ISG20 |
| JAGN1 |
| KCNJ8 |
| KIR3DL1 |
| KLRC2 |
| KLRD1 |
| KMT2E |
| LAG3 |
| LEP |
| LYST |
| MAP3K14 |
| MMP12 |
| MUL1 |
| MX1 |
| MX2 |
| MYO18A |
| MZB1 |
| NCBP3 |
| NCR1 |
| NLRC5 |
| NLRP9 |
| NT5C3A |
| OAS2 |
| OAS3 |
| OASL |
| OPRK1 |
| PARP9 |
| PCBP2 |
| PDE12 |
| PIK3R6 |
| PMAIP1 |
| PML |
| POLR3A |
| POLR3B |
| POLR3C |
| POLR3D |
| POLR3E |
| POLR3F |
| POLR3G |
| POLR3H |
| POLR3K |
| PPM1B |
| PRDX1 |
| PRDX2 |
| RBP4 |
| RNASEL |
| RNF216 |
| RNF26 |
| RTP4 |
| SAMHD1 |
| SELENOK |
| SERINC3 |
| SERINC5 |
| SERPINB4 |
| SERPINB9 |
| SETD2 |
| SKP2 |
| SLAMF8 |
| SLFN11 |
| SLFN13 |
| STAT1 |
| STAT2 |
| STAT5B |
| TARBP2 |
| TMBIM6 |
| TNFRSF4 |
| TRAF3IP1 |
| TRIM11 |
| TRIM22 |
| TRIM25 |
| TRIM34 |
| TRIM38 |
| TRIM44 |
| TRIM56 |
| TSPAN32 |
| TUSC2 |
| WDR1 |
| ZC3HAV1 |
| ZMPSTE24 |
| ZMYND11 |
| ZNF175 |
| KIR2DL1 |
| CALM1 |
| CD200R1 |
| CD24 |
| FCER1A |
| FOS |
| JUN |
| MAP2K4 |
| MAP2K7 |
| MAPK10 |
| MAPK8 |
| MAPK9 |
| MS4A2 |
| NFATC1 |
| NFATC3 |
| PPP3CA |
| PPP3R1 |
| SHC1 |
| SOS1 |
| WASLs |
| ADAM15 |
| ADAMTS13 |
| ADGRB1 |
| AIF1 |
| AKAP8 |
| AKIRIN2 |
| ANKHD1 |
| APOA4 |
| APOE |
| APOL1 |
| AQP4 |
| ARHGEF2 |
| ARID5A |
| ASS1 |
| BPIFB3 |
| CALCOCO2 |
| CAMK2A |
| CAMK2B |
| CAMK2D |
| CAMK2G |
| CAPZA1 |
| CAPZA2 |
| CASP1 |
| CASP4 |
| CCL1 |
| CCL11 |
| CCL14 |
| CCL15 |
| CCL16 |
| CCL17 |
| CCL18 |
| CCL20 |
| CCL22 |
| CCL23 |
| CCL24 |
| CCL25 |
| CCL26 |
| CCL3L1 |
| CCL3L3 |
| CCL4 |
| CCL5 |
| CCL7 |
| CCL8 |
| CD300E |
| CD300LB |
| CDC37 |
| CDC42EP2 |
| CDC42EP4 |
| CHID1 |
| CIITA |
| CITED1 |
| CLDN1 |
| COCH |
| CSF1 |
| CSF1R |
| CX3CL1 |
| CX3CR1 |
| CXCL16 |
| CYP27B1 |
| DAPK1 |
| DAPK3 |
| DCST1 |
| DEFB104A |
| DEFB104B |
| DEFB105A |
| DEFB105B |
| DEFB106A |
| DEFB106B |
| DEFB107A |
| DEFB107B |
| DEFB108A |
| DEFB108B |
| DEFB110 |
| DEFB112 |
| DEFB113 |
| DEFB114 |
| DEFB115 |
| DEFB116 |
| DEFB119 |
| DEFB121 |
| DEFB123 |
| DEFB124 |
| DEFB125 |
| DEFB128 |
| DEFB129 |
| DEFB131A |
| DEFB131B |
| DEFB132 |
| DEFB133 |
| DEFB134 |
| DEFB135 |
| DRD2 |
| ECSIT |
| EDN1 |
| EGR1 |
| ELF4 |
| EPRS |
| EREG |
| EVL |
| F12 |
| FBXO9 |
| FLNB |
| GBP2 |
| GBP4 |
| GBP5 |
| GBP6 |
| GCH1 |
| GPER1 |
| HMGB2 |
| HMGB3 |
| IFI30 |
| IFI35 |
| IFNAR1 |
| IFNGR1 |
| IFNGR2 |
| IL1RAP |
| IL1RL2 |
| IL34 |
| IL36A |
| IL36B |
| IL36G |
| IP6K2 |
| IPO7 |
| IRF6 |
| IRF8 |
| JAK1 |
| KIF16B |
| KIF5B |
| KIR2DS1 |
| KIR2DS2 |
| KIR2DS4 |
| KIR2DS5 |
| KIR3DS1 |
| KLRG1 |
| KRT16 |
| KYNU |
| LILRA5 |
| LRP8 |
| LY86 |
| MAP3K5 |
| MAP4K2 |
| MED1 |
| MEFV |
| MID1 |
| MID2 |
| MRC1 |
| MSRB1 |
| MST1R |
| MT2A |
| NAIP |
| NCAM1 |
| NCF1 |
| NCF2 |
| NCR2 |
| NLRC3 |
| NLRP1 |
| NLRP2 |
| NMI |
| NOS2 |
| NR1H2 |
| NUB1 |
| OPTN |
| OTOP1 |
| PADI4 |
| PARP14 |
| PIAS1 |
| PLA2G2F |
| PPARG |
| PPP1R14B |
| PPP6C |
| PRKD1 |
| PSTPIP1 |
| PTPN1 |
| PTPN11 |
| PYDC1 |
| PYDC2 |
| RAB12 |
| RAB20 |
| RAB43 |
| RNASE8 |
| RNF135 |
| RPL13A |
| RPS6KB1 |
| S100B |
| SAA1 |
| SCRIB |
| SDHAF4 |
| SEC61A1 |
| SHMT2 |
| SIGLEC15 |
| SIGLEC16 |
| SIRT2 |
| SLC26A6 |
| SLC30A8 |
| SNCA |
| SOCS1 |
| SOCS3 |
| SP100 |
| SRPK1 |
| SRPK2 |
| SSC5D |
| STAR |
| STX8 |
| STXBP4 |
| SUMO1 |
| SYNCRIP |
| TBKBP1 |
| TDGF1 |
| TNFAIP8L2 |
| TRAFD1 |
| TRDV1 |
| TRDV2 |
| TRDV3 |
| TREML1 |
| TRGV2 |
| TRGV3 |
| TRGV4 |
| TRGV5 |
| TRGV8 |
| TRGV9 |
| TRIM10 |
| TRIM13 |
| TRIM14 |
| TRIM21 |
| TRIM23 |
| TRIM26 |
| TRIM28 |
| TRIM29 |
| TRIM31 |
| TRIM32 |
| TRIM35 |
| TRIM4 |
| TRIM59 |
| TRIM62 |
| TRIM68 |
| TRIM8 |
| TYK2 |
| UBE2K |
| USP14 |
| USP18 |
| VAMP3 |
| VCAM1 |
| VIM |
| VPS26B |
| WRNIP1 |
| XAF1 |
| XCL2 |
| ZBP1 |
| ZYX |
| AKIRIN1 |
| ARHGEF5 |
| C10orf99 |
| CALCA |
| CALR |
| CAMK1D |
| CCN3 |
| CCR1 |
| CCR5 |
| CD300H |
| CH25H |
| CKLF |
| CNR2 |
| CREB3 |
| CSF3R |
| CXADR |
| CXCL14 |
| CXCL17 |
| CXCR3 |
| CXCR4 |
| CXCR5 |
| CYP19A1 |
| CYP7B1 |
| DAPK2 |
| DDT |
| DUSP1 |
| EDN2 |
| EDN3 |
| EDNRB |
| F7 |
| FLT1 |
| GPR18 |
| GPSM3 |
| GREM1 |
| HRH1 |
| HSD3B7 |
| IL16 |
| IL17RA |
| IL17RC |
| IL1F10 |
| IL1RN |
| IL37 |
| ITGA1 |
| ITGA9 |
| JAML |
| MMP28 |
| MPP1 |
| MST1 |
| MST1L |
| MSTN |
| MTUS1 |
| NBL1 |
| NUP85 |
| OXSR1 |
| PDGFB |
| PGF |
| PIP5K1C |
| PLA2G7 |
| PREX1 |
| PTPRO |
| RIPOR2 |
| S1PR1 |
| SBDS |
| SCG2 |
| SERPINE1 |
| SLC12A2 |
| SLC8B1 |
| SLIT2 |
| STK39 |
| THBS1 |
| THBS4 |
| TMEM102 |
| TNFSF11 |
| TNFSF14 |
| TRPV4 |
| VEGFA |
| VEGFB |
| VEGFC |
| VEGFD |
| ZNF580 |
| BLOC1S3 |
| CD2 |
| FLT3LG |
| HNF1A |
| ID2 |
| IL21R |
| MERTK |
| PIBF1 |
| RHBDD3 |
| SNX27 |
| SP3 |
| TOX |
| CNR1 |
| IDO1 |
| MIR136 |
| PARK7 |
| ACIN1 |
| ACVR1B |
| ACVR2A |
| AKT1 |
| ANKRD54 |
| AP3B1 |
| AP3D1 |
| AQP3 |
| ARNT |
| ATP11C |
| ATP6AP1 |
| ATXN1L |
| BAD |
| BDKRB1 |
| BLM |
| CA2 |
| CCDC88B |
| CD101 |
| CD320 |
| CD5 |
| CD99L2 |
| CEBPA |
| CHRNB2 |
| CLECL1 |
| CREB1 |
| CRLF2 |
| CSF3 |
| CTNNBIP1 |
| DNAJA3 |
| DOCK8 |
| DPP4 |
| EFNB1 |
| EFNB2 |
| EFNB3 |
| EGR3 |
| EPO |
| ETS1 |
| EVI2B |
| FAM210B |
| FGF10 |
| FLOT2 |
| FOXC1 |
| FOXO3 |
| GLI2 |
| GLI3 |
| GNAS |
| GPR68 |
| HAX1 |
| HCAR2 |
| HCLS1 |
| HES1 |
| HIF1A |
| HOXA5 |
| HSPH1 |
| ICOS |
| IGF1 |
| IGF2 |
| IGFBP2 |
| IHH |
| IL17A |
| IL20 |
| INHBA |
| IRS2 |
| ITGA2 |
| ITGA2B |
| ITGA4 |
| ITPKB |
| KITLG |
| KLF10 |
| LIF |
| LRRK2 |
| MADCAM1 |
| MAP3K8 |
| MIA3 |
| MIR128-1 |
| MIR128-2 |
| MIR142 |
| MIR221 |
| MIR222 |
| MIR30B |
| MMP14 |
| MPL |
| N4BP2L2 |
| NCK2 |
| NKAP |
| NUDT21 |
| OCSTAMP |
| P2RX4 |
| P2RY12 |
| PCID2 |
| PDCD1LG2 |
| PDCD2 |
| PDGFD |
| PLVAP |
| POU4F1 |
| POU4F2 |
| PPARGC1B |
| PRKCA |
| PRMT1 |
| RASAL3 |
| RB1 |
| RHEX |
| ROR2 |
| RUNX3 |
| SART1 |
| SCIN |
| SELP |
| SHH |
| SIRPG |
| SLC9B2 |
| SMAP1 |
| SPACA3 |
| SPTA1 |
| TAC1 |
| TAFA3 |
| TAL1 |
| TESC |
| THPO |
| TMEM64 |
| TMIGD2 |
| TNFRSF18 |
| TNFSF9 |
| TRIB1 |
| TRIM58 |
| TSLP |
| TTBK1 |
| VSIR |
| WNT3A |
| ZBTB16 |
| ZFP36L1 |
| ZMIZ1 |
| ZNF16 |
| ZNF335 |
| ADCYAP1 |
| AMBP |
| CD200 |
| CD300C |
| CD300LD |
| CD300LG |
| CD34 |
| CD99 |
| CLEC2B |
| CLEC2D |
| COL17A1 |
| COL1A1 |
| COL1A2 |
| COL2A1 |
| COL3A1 |
| DNASE2 |
| ECM1 |
| FCRLB |
| FKBP1A |
| GLYCAM1 |
| GPR17 |
| GPX1 |
| HCST |
| ICAM4 |
| ICAM5 |
| ITGB1 |
| ITGB7 |
| KIR2DL2 |
| KIR2DL3 |
| KIR3DL2 |
| KLRB1 |
| KLRC1 |
| KLRF1 |
| LAIR2 |
| NCR3LG1 |
| NPDC1 |
| NPY5R |
| PIANP |
| PILRA |
| PILRB |
| SELENOS |
| SIGLEC7 |
| SMAD3 |
| SPPL2A |
| SPPL2B |
| TREML2 |
| ADGRF5 |
| ADIPOQ |
| ADORA1 |
| ADORA2A |
| ADTRP |
| AGO1 |
| AGO3 |
| AGO4 |
| AHR |
| APOD |
| ASH2L |
| ATM |
| BANK1 |
| BGLAP |
| BMP4 |
| BMP5 |
| CARTPT |
| CASP3 |
| CCL28 |
| CDC73 |
| CDK6 |
| CIB1 |
| CLDN18 |
| CLPTM1 |
| CNOT4 |
| CTNNB1 |
| CTR9 |
| CUL4A |
| CYP26B1 |
| DLG5 |
| DPF2 |
| DPY30 |
| DROSHA |
| DTX1 |
| EIF6 |
| EMILIN1 |
| ERBB2 |
| ESRRA |
| FANCA |
| FANCD2 |
| FBN1 |
| FBXO7 |
| FBXW7 |
| FLCN |
| FNIP1 |
| FOXN1 |
| FSHB |
| FSTL3 |
| GABPA |
| GAL |
| GGT1 |
| GGT2 |
| GGT3P |
| GLMN |
| GNRH1 |
| GP1BA |
| GPNMB |
| GPR171 |
| GPR55 |
| H3F3A |
| H3F3B |
| HDAC1 |
| HES5 |
| HIST1H3A |
| HIST1H3B |
| HIST1H3C |
| HIST1H3D |
| HIST1H3E |
| HIST1H3F |
| HIST1H3G |
| HIST1H3H |
| HIST1H3I |
| HIST1H3J |
| HIST1H4A |
| HIST1H4B |
| HIST1H4C |
| HIST1H4D |
| HIST1H4E |
| HIST1H4F |
| HIST1H4H |
| HIST1H4I |
| HIST1H4J |
| HIST1H4K |
| HIST1H4L |
| HIST2H3A |
| HIST2H3C |
| HIST2H3D |
| HIST2H4A |
| HIST2H4B |
| HIST4H4 |
| HLA-DOA |
| HLA-DOB |
| HOXA7 |
| HOXA9 |
| HOXB8 |
| HSPA9 |
| IKZF3 |
| IL17D |
| INHA |
| KAT2A |
| KAT2B |
| KLF13 |
| KMT2A |
| KMT2B |
| KMT2C |
| KMT2D |
| L3MBTL1 |
| LDB1 |
| LDLR |
| LEO1 |
| LMO1 |
| LMO2 |
| LOX |
| LRCH1 |
| LRFN5 |
| LRRC17 |
| LRRC32 |
| LST1 |
| MAD1L1 |
| MAFB |
| MAPK8IP1 |
| 7-Mar |
| MEIS1 |
| MEIS2 |
| METTL3 |
| MIR124-1 |
| MIR124-2 |
| MIR124-3 |
| MIR125B1 |
| MIR125B2 |
| MIR17HG |
| MIR24-1 |
| MIR24-2 |
| MIR27A |
| MITF |
| MIXL1 |
| MOV10 |
| MSN |
| MYC |
| MYL9 |
| MYSM1 |
| NCAPG2 |
| NF1 |
| NFE2 |
| NFE2L2 |
| NME1 |
| NRARP |
| P4HTM |
| PAF1 |
| PDE5A |
| PHLPP1 |
| PIAS3 |
| PLA2G10 |
| PLA2G2D |
| PLA2G5 |
| PLCB1 |
| PRELID1 |
| PRKAR1A |
| PRMT6 |
| PRXL2A |
| PURB |
| PUS7 |
| RARG |
| RASSF2 |
| RBBP5 |
| RBFOX2 |
| RBM15 |
| RNF41 |
| SCGB1A1 |
| SDC4 |
| SENP1 |
| SETD1A |
| SFRP1 |
| SH3RF1 |
| SHPK |
| SIAE |
| SLC46A2 |
| SLC7A11 |
| SLC7A2 |
| SMPD3 |
| SOCS6 |
| SOD1 |
| SOX11 |
| SOX13 |
| SOX9 |
| TAPBPL |
| TCF12 |
| TCF3 |
| TCTA |
| TIGIT |
| TMEM131L |
| TMEM178A |
| TNRC6A |
| TNRC6B |
| TNRC6C |
| TOB2 |
| TP73 |
| TSC22D3 |
| TWSG1 |
| UBASH3B |
| WDR5 |
| WDR61 |
| YAP1 |
| ZC3H8 |
| ZEB1 |
| ZFP36 |
| ZFP36L2 |
| ZNF675 |
