# Supplementary material for: A Novel Immune-Related lncRNA-Based Model for Survival Prediction in Clear Cell Renal Cell Carcinoma
Source: J Immunol Res. 2021 Jun 28;2021:9921466. doi: 10.1155/2021/9921466 (PMC8339875; doi:10.1155/2021/9921466)
Supplement: Supplementary 3 — Table S3: 280 immune-related DElncRNAs. [file 9921466.f3.docx]

| **Table S3: 280 immune-related DElncRNAs** | |
| --- | --- |
| **LncRNA** | **Ensemble ID** |
| AC002091.1 | ENSG00000265975 |
| AC002091.2 | ENSG00000266389 |
| AC002128.1 | ENSG00000268947 |
| AC003070.1 | ENSG00000267344 |
| AC003984.1 | ENSG00000235139 |
| AC004148.2 | ENSG00000263272 |
| AC004253.1 | ENSG00000263990 |
| AC004263.2 | ENSG00000275936 |
| AC004264.2 | ENSG00000268812 |
| AC004494.1 | ENSG00000262312 |
| AC004585.1 | ENSG00000266088 |
| AC004687.1 | ENSG00000265206 |
| AC004847.1 | ENSG00000260997 |
| AC004865.2 | ENSG00000239636 |
| AC004921.1 | ENSG00000273341 |
| AC005104.1 | ENSG00000223374 |
| AC005154.1 | ENSG00000196295 |
| AC005253.1 | ENSG00000268030 |
| AC005281.1 | ENSG00000225606 |
| AC005306.1 | ENSG00000267283 |
| AC005519.2 | ENSG00000258559 |
| AC006033.2 | ENSG00000272908 |
| AC006369.1 | ENSG00000236213 |
| AC006435.2 | ENSG00000263345 |
| AC007038.2 | ENSG00000272807 |
| AC007386.2 | ENSG00000232613 |
| AC007406.2 | ENSG00000255746 |
| AC007406.3 | ENSG00000256540 |
| AC007566.1 | ENSG00000244055 |
| AC007728.2 | ENSG00000261644 |
| AC007728.3 | ENSG00000270120 |
| AC007743.1 | ENSG00000233251 |
| AC008105.3 | ENSG00000267121 |
| AC008735.2 | ENSG00000267523 |
| AC008741.2 | ENSG00000274925 |
| AC008760.2 | ENSG00000276980 |
| AC008764.8 | ENSG00000279529 |
| AC008870.2 | ENSG00000260751 |
| AC009093.1 | ENSG00000259807 |
| AC009120.2 | ENSG00000259972 |
| AC009133.3 | ENSG00000260719 |
| AC010501.1 | ENSG00000271714 |
| AC010776.3 | ENSG00000273664 |
| AC010973.2 | ENSG00000244151 |
| AC010976.1 | ENSG00000231731 |
| AC011462.4 | ENSG00000277744 |
| AC011481.1 | ENSG00000267114 |
| AC011899.2 | ENSG00000233038 |
| AC011899.3 | ENSG00000272839 |
| AC012236.1 | ENSG00000259772 |
| AC012615.6 | ENSG00000267244 |
| AC012645.3 | ENSG00000261416 |
| AC012645.4 | ENSG00000275371 |
| AC013286.1 | ENSG00000230575 |
| AC015911.3 | ENSG00000267074 |
| AC015914.1 | ENSG00000259351 |
| AC016773.1 | ENSG00000270195 |
| AC018755.4 | ENSG00000273837 |
| AC020907.4 | ENSG00000271032 |
| AC020916.1 | ENSG00000267519 |
| AC020931.1 | ENSG00000267387 |
| AC022537.1 | ENSG00000231132 |
| AC023825.2 | ENSG00000260145 |
| AC024361.3 | ENSG00000263321 |
| AC027601.1 | ENSG00000260005 |
| AC027601.2 | ENSG00000262115 |
| AC027796.4 | ENSG00000262903 |
| AC034238.1 | ENSG00000240535 |
| AC040162.3 | ENSG00000263126 |
| AC048341.3 | ENSG00000275180 |
| AC053513.1 | ENSG00000256973 |
| AC068858.1 | ENSG00000254654 |
| AC078778.1 | ENSG00000257596 |
| AC078950.1 | ENSG00000255790 |
| AC087289.2 | ENSG00000267342 |
| AC087318.1 | ENSG00000256714 |
| AC087482.1 | ENSG00000259347 |
| AC087741.1 | ENSG00000262580 |
| AC090152.1 | ENSG00000167912 |
| AC090241.2 | ENSG00000270112 |
| AC090589.3 | ENSG00000270060 |
| AC092118.2 | ENSG00000276166 |
| AC092535.4 | ENSG00000273179 |
| AC093904.2 | ENSG00000242136 |
| AC093904.4 | ENSG00000261104 |
| AC097534.2 | ENSG00000272870 |
| AC100803.2 | ENSG00000261655 |
| AC100803.3 | ENSG00000271959 |
| AC103809.1 | ENSG00000266106 |
| AC104072.1 | ENSG00000260878 |
| AC104564.4 | ENSG00000264290 |
| AC104699.1 | ENSG00000224220 |
| AC104984.4 | ENSG00000266120 |
| AC105046.1 | ENSG00000261026 |
| AC106882.1 | ENSG00000248571 |
| AC108134.3 | ENSG00000262370 |
| AC109460.2 | ENSG00000260853 |
| AC109460.3 | ENSG00000261067 |
| AC109826.1 | ENSG00000226791 |
| AC110285.2 | ENSG00000262877 |
| AC114730.3 | ENSG00000235351 |
| AC114811.2 | ENSG00000260641 |
| AC115619.1 | ENSG00000261012 |
| AC116366.1 | ENSG00000234290 |
| AC116914.2 | ENSG00000262692 |
| AC127024.4 | ENSG00000265791 |
| AC129510.1 | ENSG00000265678 |
| AC132872.2 | ENSG00000264548 |
| AC132872.3 | ENSG00000275888 |
| AC135050.3 | ENSG00000260911 |
| AC136604.2 | ENSG00000244945 |
| AC137932.3 | ENSG00000268218 |
| AC138207.5 | ENSG00000265743 |
| AC145098.1 | ENSG00000248996 |
| AC232271.1 | ENSG00000270012 |
| AC243829.2 | ENSG00000276241 |
| AC243829.4 | ENSG00000277089 |
| AC243960.1 | ENSG00000268027 |
| AC244205.1 | ENSG00000240040 |
| AC245052.4 | ENSG00000237017 |
| AC245884.8 | ENSG00000267838 |
| AC246787.2 | ENSG00000244620 |
| AC254629.1 | ENSG00000274993 |
| AF127936.1 | ENSG00000226751 |
| AGAP2-AS1 | ENSG00000255737 |
| AL020994.2 | ENSG00000226741 |
| AL021707.6 | ENSG00000272669 |
| AL022322.1 | ENSG00000272720 |
| AL022328.2 | ENSG00000273137 |
| AL031058.1 | ENSG00000261189 |
| AL031714.1 | ENSG00000261505 |
| AL034397.3 | ENSG00000274536 |
| AL049795.1 | ENSG00000224066 |
| AL049840.1 | ENSG00000246451 |
| AL080317.1 | ENSG00000230177 |
| AL096799.1 | ENSG00000232271 |
| AL109741.3 | ENSG00000260940 |
| AL109811.1 | ENSG00000226849 |
| AL117209.1 | ENSG00000259515 |
| AL117335.1 | ENSG00000276649 |
| AL132989.1 | ENSG00000258682 |
| AL133325.3 | ENSG00000278041 |
| AL133371.2 | ENSG00000258810 |
| AL135818.1 | ENSG00000258875 |
| AL135960.1 | ENSG00000226252 |
| AL135999.1 | ENSG00000258727 |
| AL135999.3 | ENSG00000274002 |
| AL136084.3 | ENSG00000270412 |
| AL136088.1 | ENSG00000254489 |
| AL136295.2 | ENSG00000259321 |
| AL139120.1 | ENSG00000233340 |
| AL139287.1 | ENSG00000240731 |
| AL157935.2 | ENSG00000257524 |
| AL161935.3 | ENSG00000237797 |
| AL162414.1 | ENSG00000227531 |
| AL353699.1 | ENSG00000255480 |
| AL355075.2 | ENSG00000258515 |
| AL355075.4 | ENSG00000259001 |
| AL355488.2 | ENSG00000273373 |
| AL355803.1 | ENSG00000237371 |
| AL355922.2 | ENSG00000258471 |
| AL356488.2 | ENSG00000270066 |
| AL357060.3 | ENSG00000237499 |
| AL365361.1 | ENSG00000259834 |
| AL391095.2 | ENSG00000275285 |
| AL391845.2 | ENSG00000233542 |
| AL512770.1 | ENSG00000228302 |
| AL589745.1 | ENSG00000230731 |
| AL589863.1 | ENSG00000268659 |
| AL590764.1 | ENSG00000228427 |
| AL591468.1 | ENSG00000226004 |
| AL592164.1 | ENSG00000261435 |
| AL662884.1 | ENSG00000273333 |
| AL683807.1 | ENSG00000223511 |
| AL928654.3 | ENSG00000257270 |
| ANKRD10-IT1 | ENSG00000229152 |
| AP000802.1 | ENSG00000247416 |
| AP001029.2 | ENSG00000267199 |
| AP001107.4 | ENSG00000254461 |
| AP001160.1 | ENSG00000256690 |
| AP001341.1 | ENSG00000222042 |
| AP002807.1 | ENSG00000255031 |
| AP003392.1 | ENSG00000254428 |
| AP003774.4 | ENSG00000236935 |
| AP006284.1 | ENSG00000254815 |
| AP006621.2 | ENSG00000255142 |
| AP4B1-AS1 | ENSG00000226167 |
| ARHGAP27P1-BPTFP1-KPNA2P3 | ENSG00000215769 |
| ASMTL-AS1 | ENSG00000236017 |
| BX255925.1 | ENSG00000260996 |
| C15orf59-AS1 | ENSG00000260469 |
| C1RL-AS1 | ENSG00000205885 |
| C9orf139 | ENSG00000180539 |
| CARMN | ENSG00000249669 |
| CR559946.2 | ENSG00000279182 |
| DPP9-AS1 | ENSG00000205790 |
| EDRF1-AS1 | ENSG00000236991 |
| FAM13A-AS1 | ENSG00000248019 |
| FAM30A | ENSG00000226777 |
| GAS5 | ENSG00000234741 |
| GAS6-AS2 | ENSG00000272695 |
| HCP5 | ENSG00000206337 |
| HLA-DQB1-AS1 | ENSG00000223534 |
| IL20RB-AS1 | ENSG00000249407 |
| ITGB2-AS1 | ENSG00000227039 |
| KCNQ5-IT1 | ENSG00000233844 |
| L3MBTL4-AS1 | ENSG00000264707 |
| LBX2-AS1 | ENSG00000257702 |
| LHFPL3-AS2 | ENSG00000225329 |
| LINC00106 | ENSG00000236871 |
| LINC00158 | ENSG00000185433 |
| LINC00174 | ENSG00000179406 |
| LINC00307 | ENSG00000227342 |
| LINC00342 | ENSG00000232931 |
| LINC00426 | ENSG00000238121 |
| LINC00528 | ENSG00000269220 |
| LINC00582 | ENSG00000229228 |
| LINC00588 | ENSG00000215117 |
| LINC00686 | ENSG00000237687 |
| LINC00861 | ENSG00000245164 |
| LINC00887 | ENSG00000214145 |
| LINC00892 | ENSG00000233093 |
| LINC00893 | ENSG00000241769 |
| LINC00894 | ENSG00000235703 |
| LINC00924 | ENSG00000259134 |
| LINC00989 | ENSG00000250334 |
| LINC01094 | ENSG00000251442 |
| LINC01146 | ENSG00000258867 |
| LINC01150 | ENSG00000229671 |
| LINC01176 | ENSG00000281404 |
| LINC01230 | ENSG00000281769 |
| LINC01260 | ENSG00000132832 |
| LINC01281 | ENSG00000235304 |
| LINC01355 | ENSG00000261326 |
| LINC01415 | ENSG00000267325 |
| LINC01428 | ENSG00000228888 |
| LINC01544 | ENSG00000260440 |
| LINC01561 | ENSG00000177234 |
| LINC01614 | ENSG00000230838 |
| LINC01684 | ENSG00000237484 |
| LINC01738 | ENSG00000227947 |
| LINC01739 | ENSG00000229537 |
| LINC01747 | ENSG00000230400 |
| LINC01786 | ENSG00000230415 |
| LINC01857 | ENSG00000224137 |
| LINC01934 | ENSG00000234663 |
| LINC01975 | ENSG00000263312 |
| LINC01987 | ENSG00000267790 |
| LINC02048 | ENSG00000228271 |
| LINC02084 | ENSG00000272282 |
| LINC02188 | ENSG00000261175 |
| LINC02285 | ENSG00000259004 |
| LINC02325 | ENSG00000246084 |
| LINC02362 | ENSG00000249096 |
| LINC02416 | ENSG00000257924 |
| LINC02446 | ENSG00000256039 |
| MIAT | ENSG00000225783 |
| MIR155HG | ENSG00000234883 |
| N4BP2L2-IT2 | ENSG00000281026 |
| NARF-IT1 | ENSG00000266236 |
| NEAT1 | ENSG00000245532 |
| PCED1B-AS1 | ENSG00000247774 |
| PDXDC2P-NPIPB14P | ENSG00000196696 |
| PLCB1-IT1 | ENSG00000225479 |
| PP12613 | ENSG00000226757 |
| PRKCQ-AS1 | ENSG00000237943 |
| PSMB8-AS1 | ENSG00000204261 |
| PTOV1-AS2 | ENSG00000269352 |
| RAP2C-AS1 | ENSG00000232160 |
| RMRP | ENSG00000269900 |
| RUSC1-AS1 | ENSG00000225855 |
| SIRPG-AS1 | ENSG00000237914 |
| SNAP25-AS1 | ENSG00000227906 |
| STAG3L5P-PVRIG2P-PILRB | ENSG00000272752 |
| TRG-AS1 | ENSG00000281103 |
| TSPOAP1-AS1 | ENSG00000265148 |
| U62317.3 | ENSG00000272821 |
| USP30-AS1 | ENSG00000256262 |
| ZFHX2-AS1 | ENSG00000157306 |
| FO393401.1 | [ENSG00000242507](https://www.genecards.org/cgi-bin/carddisp.pl?gene=ENSG00000242507&keywords=FO393401,1) |
