# Supplementary figures and images for: A Novel Immune-Related lncRNA-Based Model for Survival Prediction in Clear Cell Renal Cell Carcinoma
Source: J Immunol Res. 2021 Jun 28;2021:9921466. doi: 10.1155/2021/9921466 (PMC8339875; doi:10.1155/2021/9921466)

**A**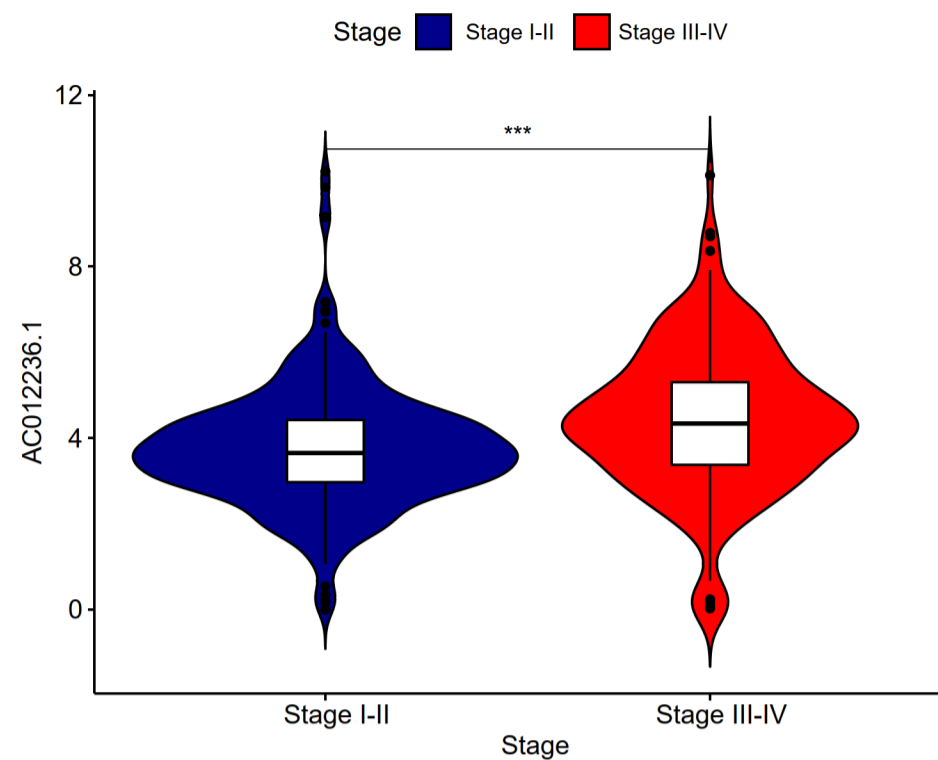**B**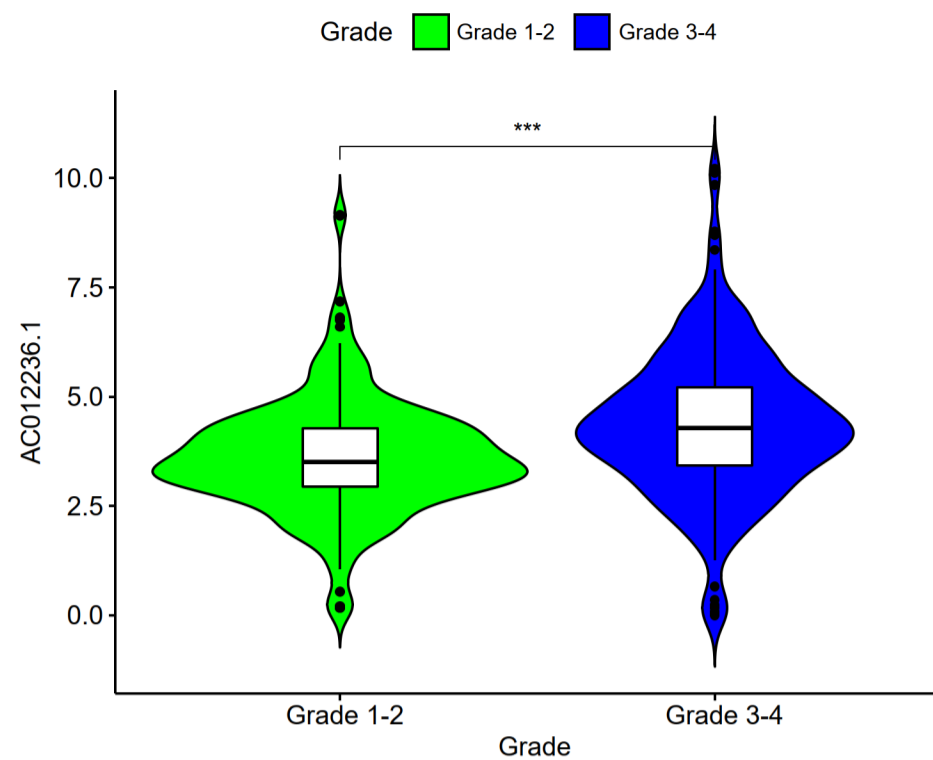**C**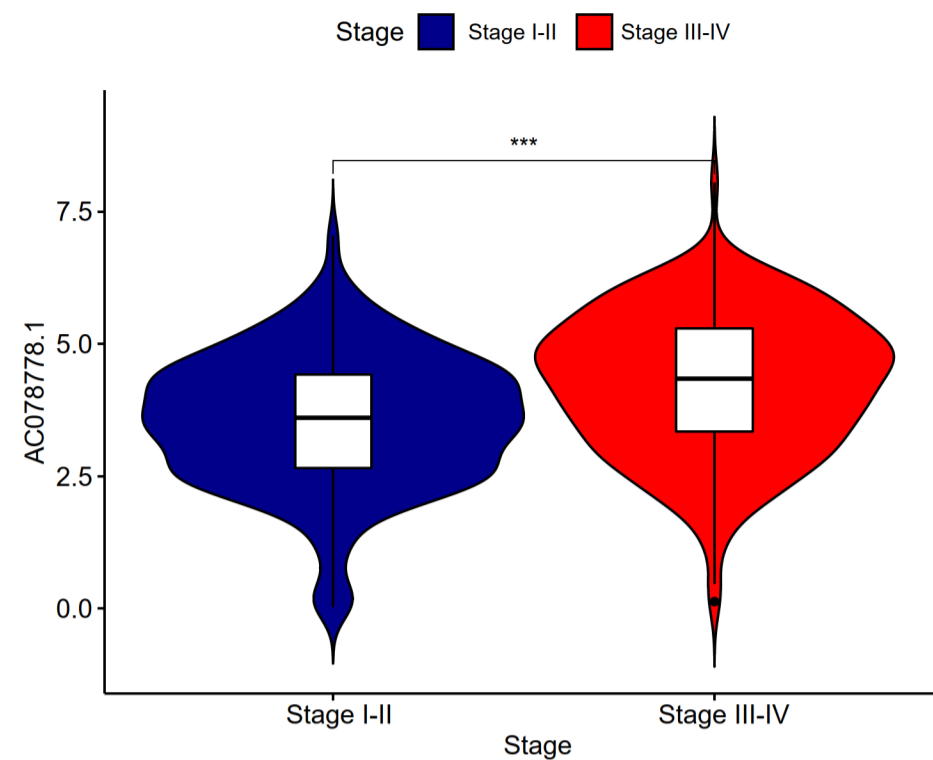**D**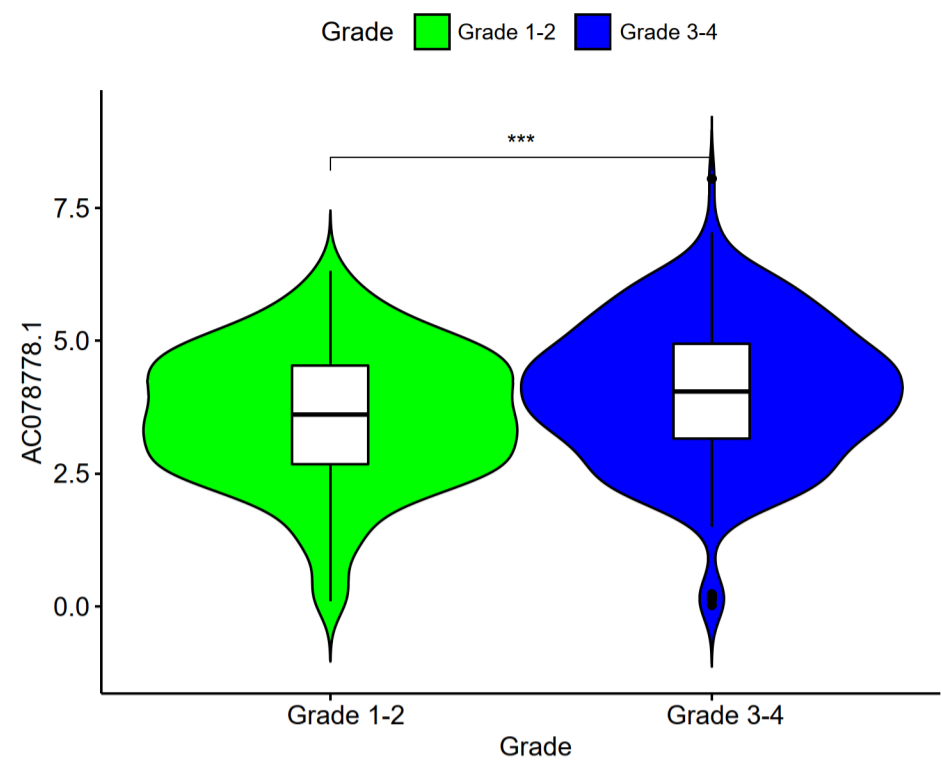**E**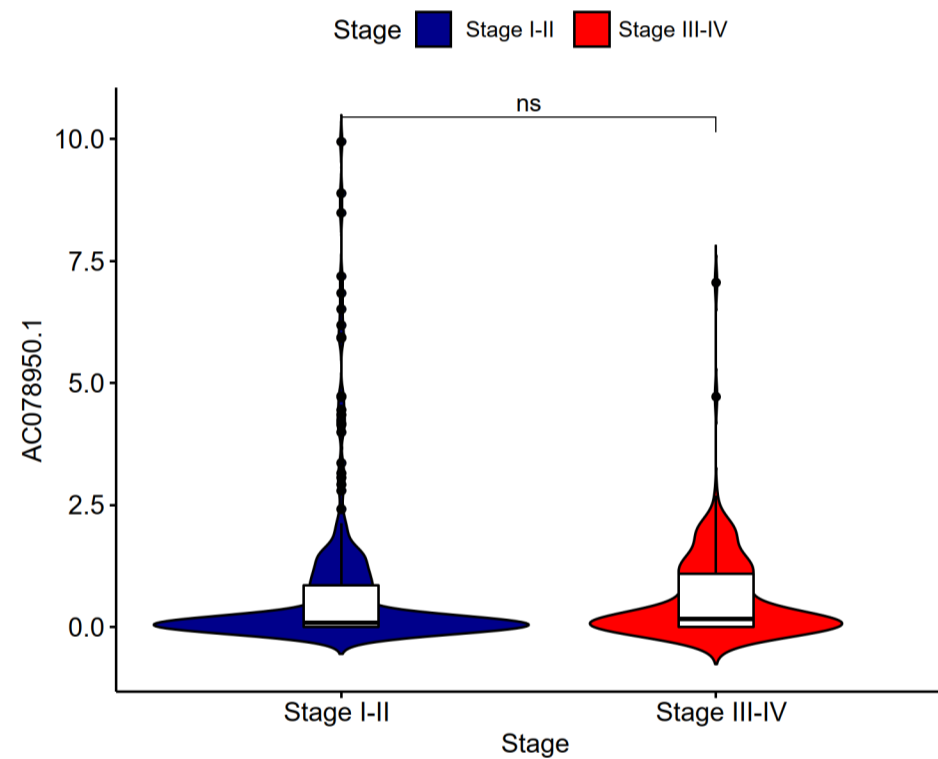**F**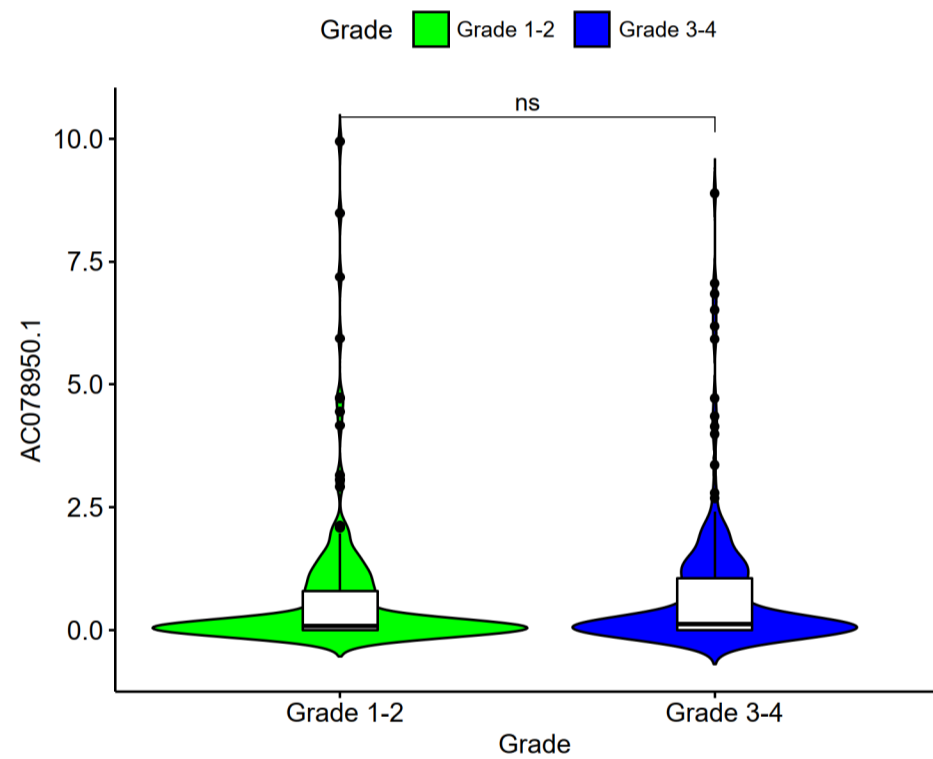**G**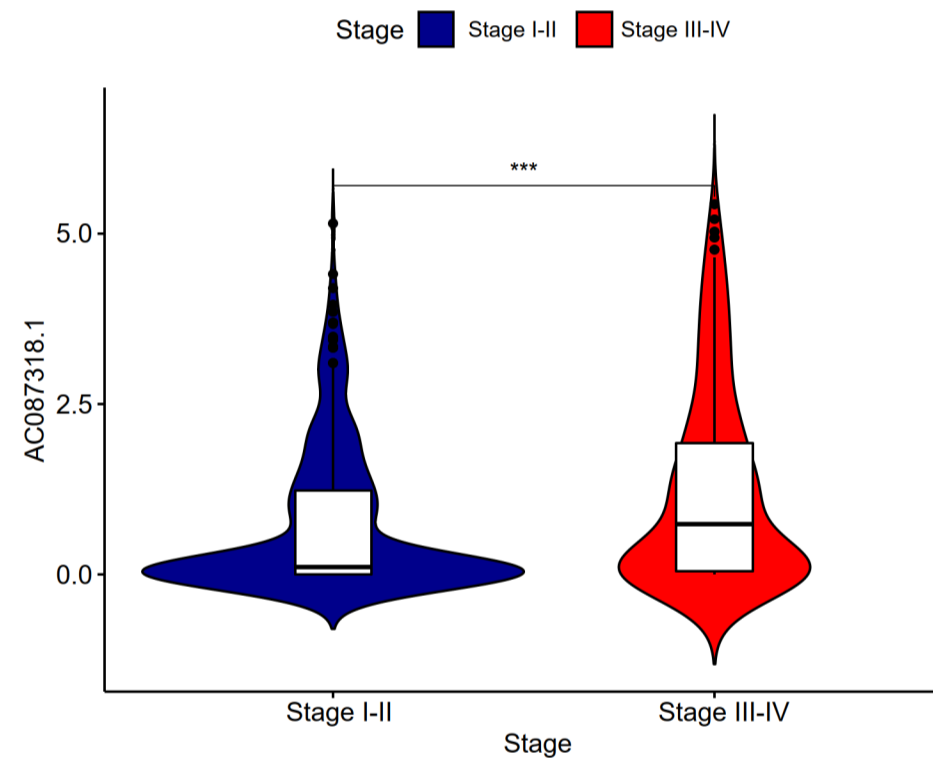**H**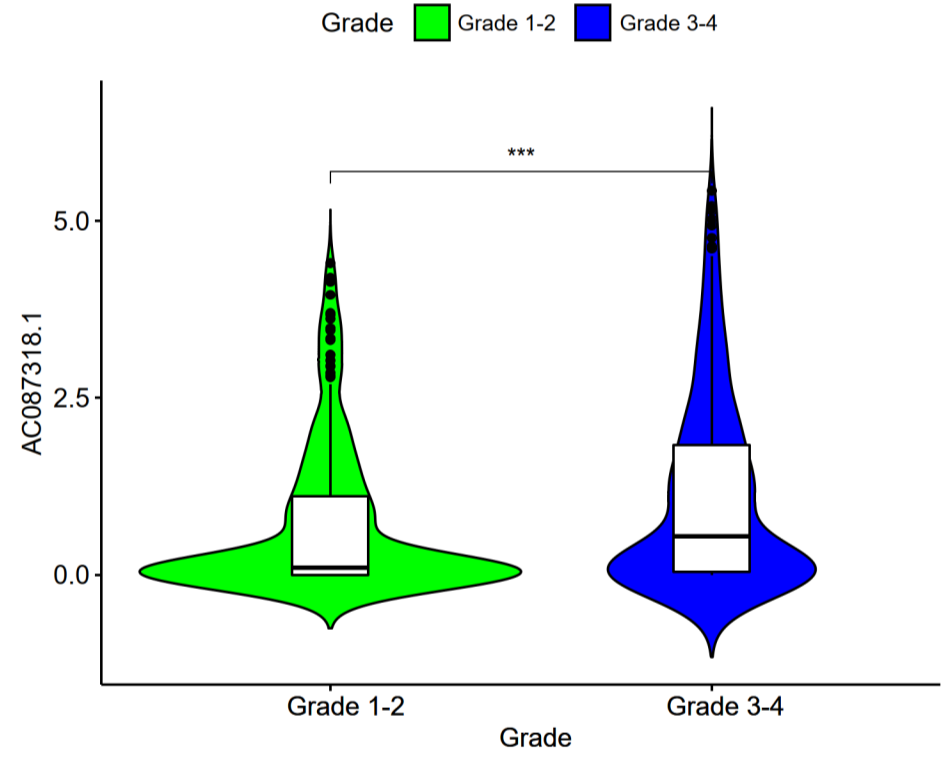**I**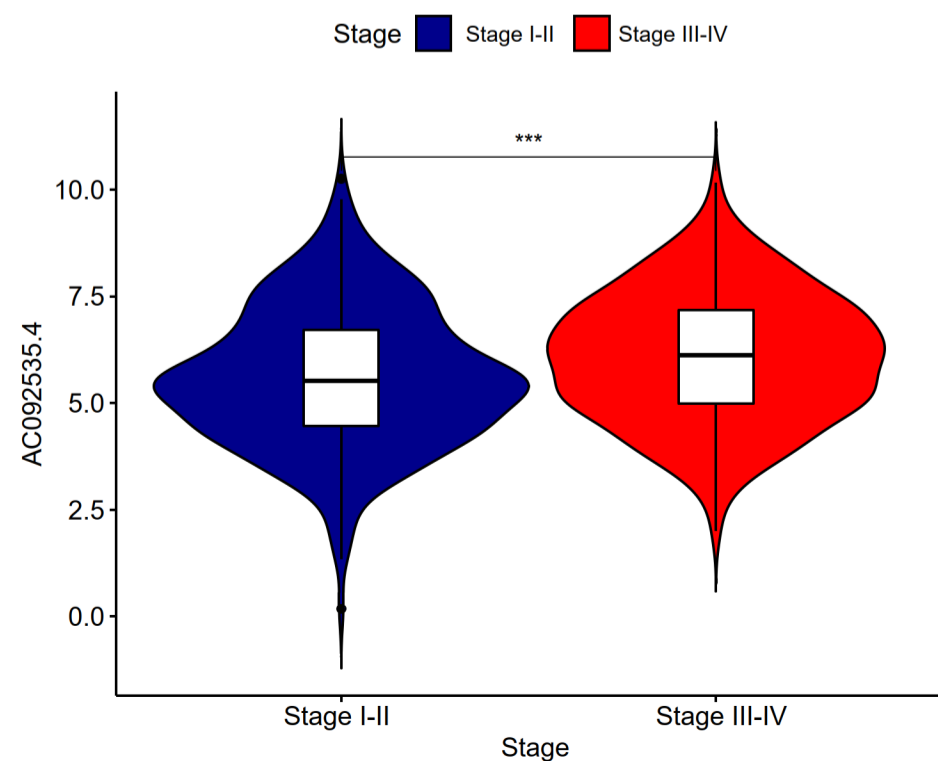**J**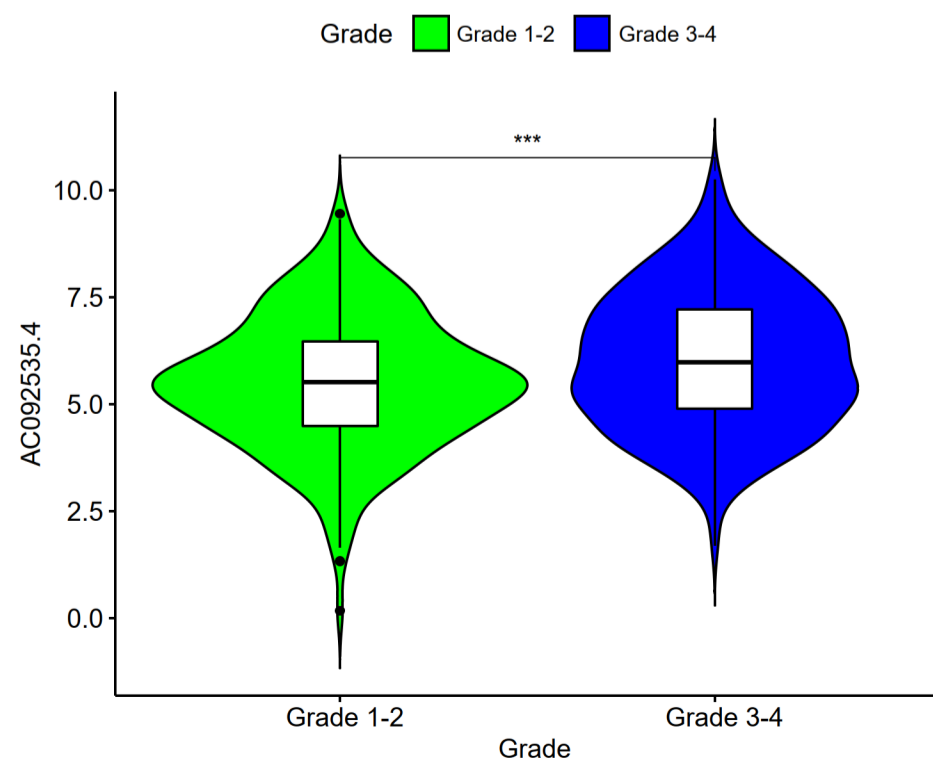**K**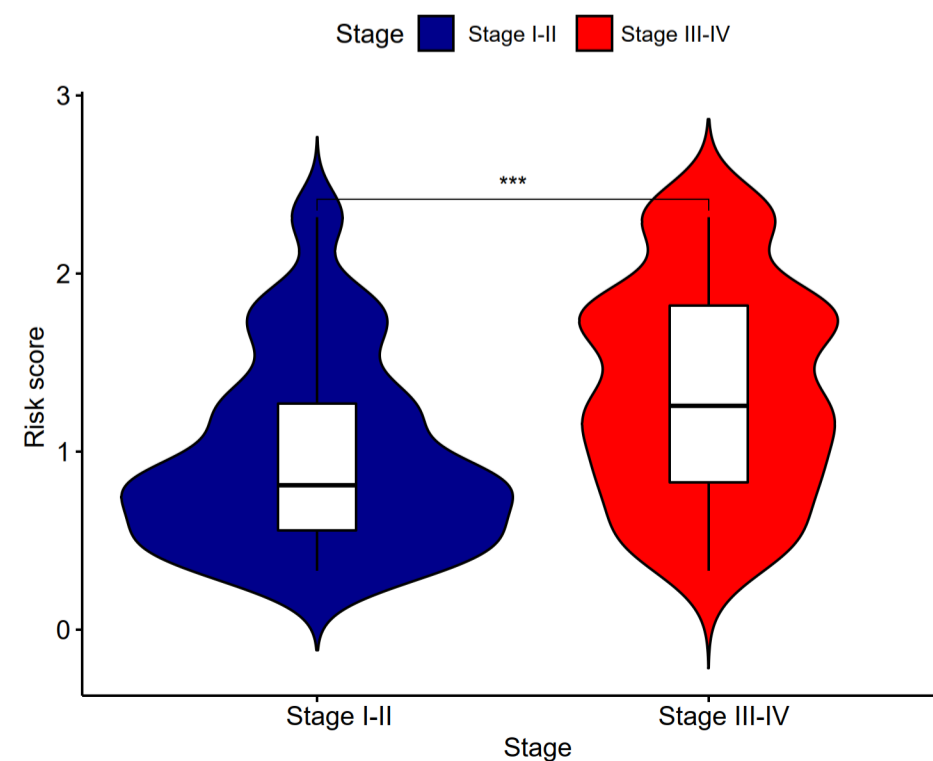**L**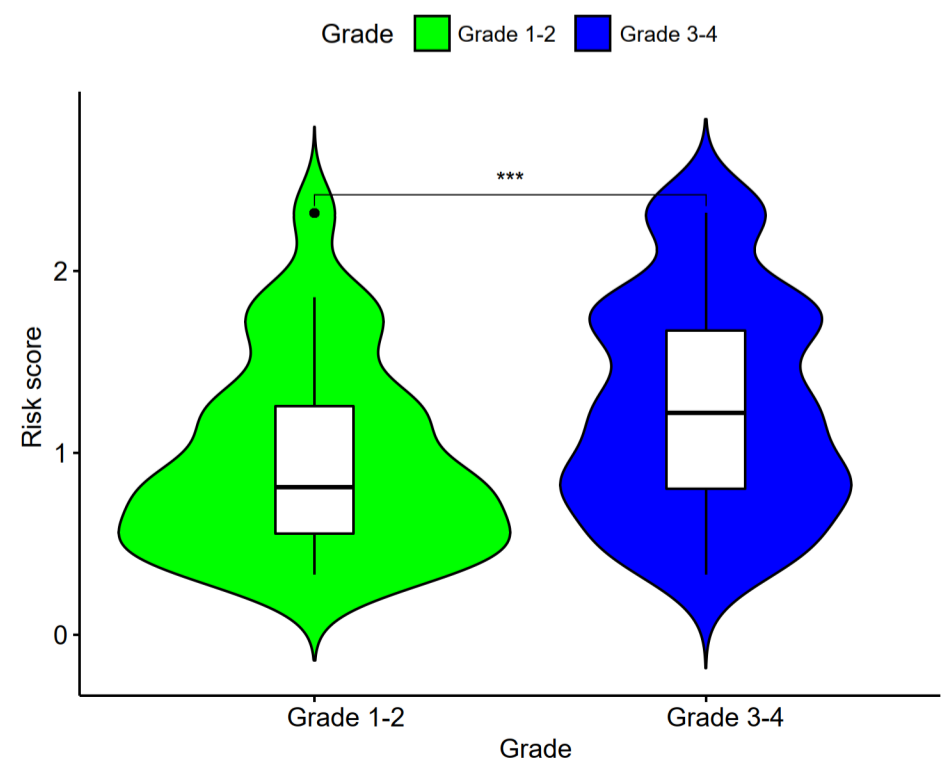

Supplement: Supplementary 4 — Figure S1: the expression pattern of the five PIDElncRNAs and the risk score in different AJCC-stage and Fuhrman grade. (A-J) AC012236.1, AC078778.1, AC078950.1, AC087318.1, and AC092535.4. (K-L) The risk score. [file 9921466.f4.pdf]

A

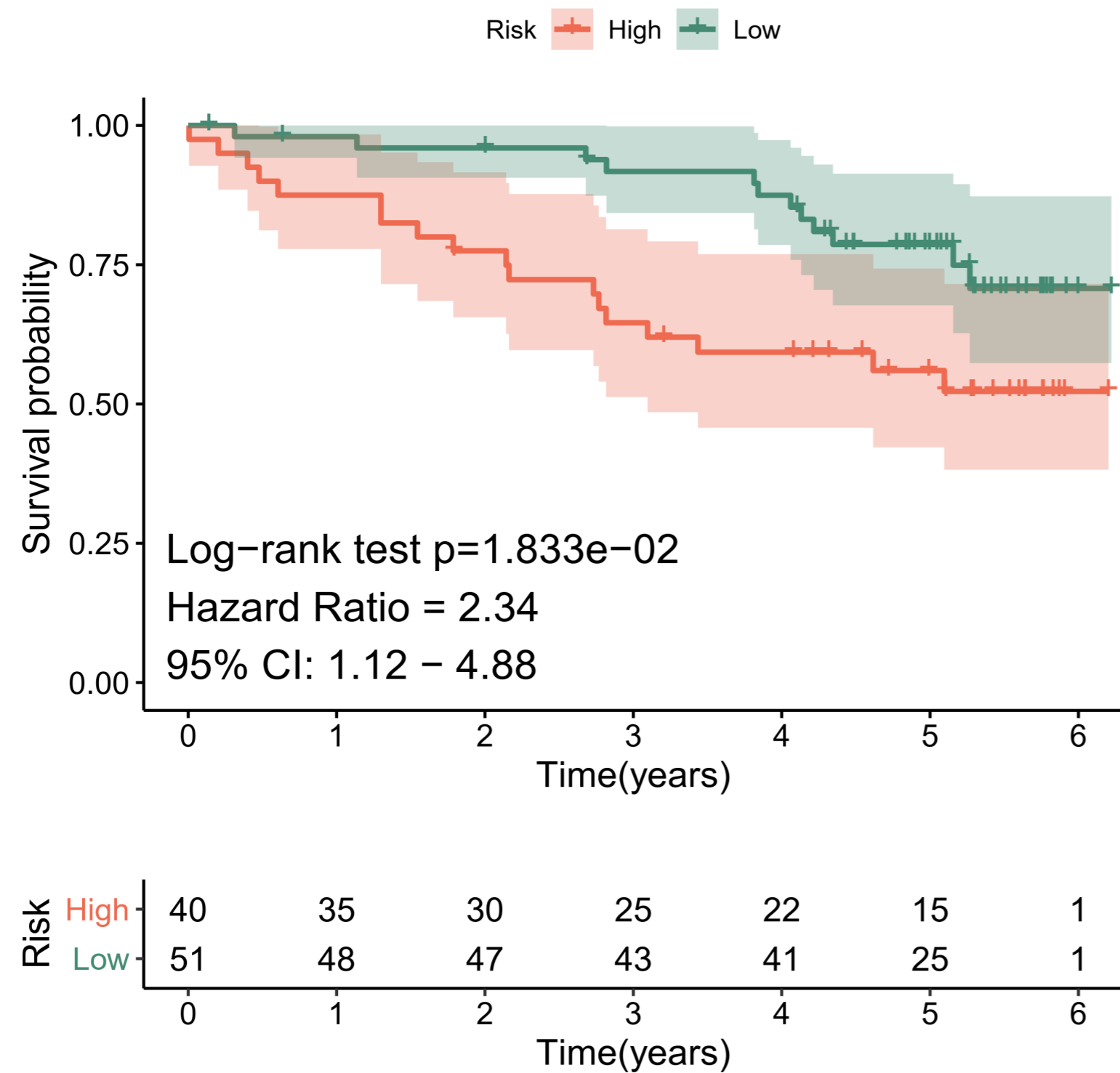

B

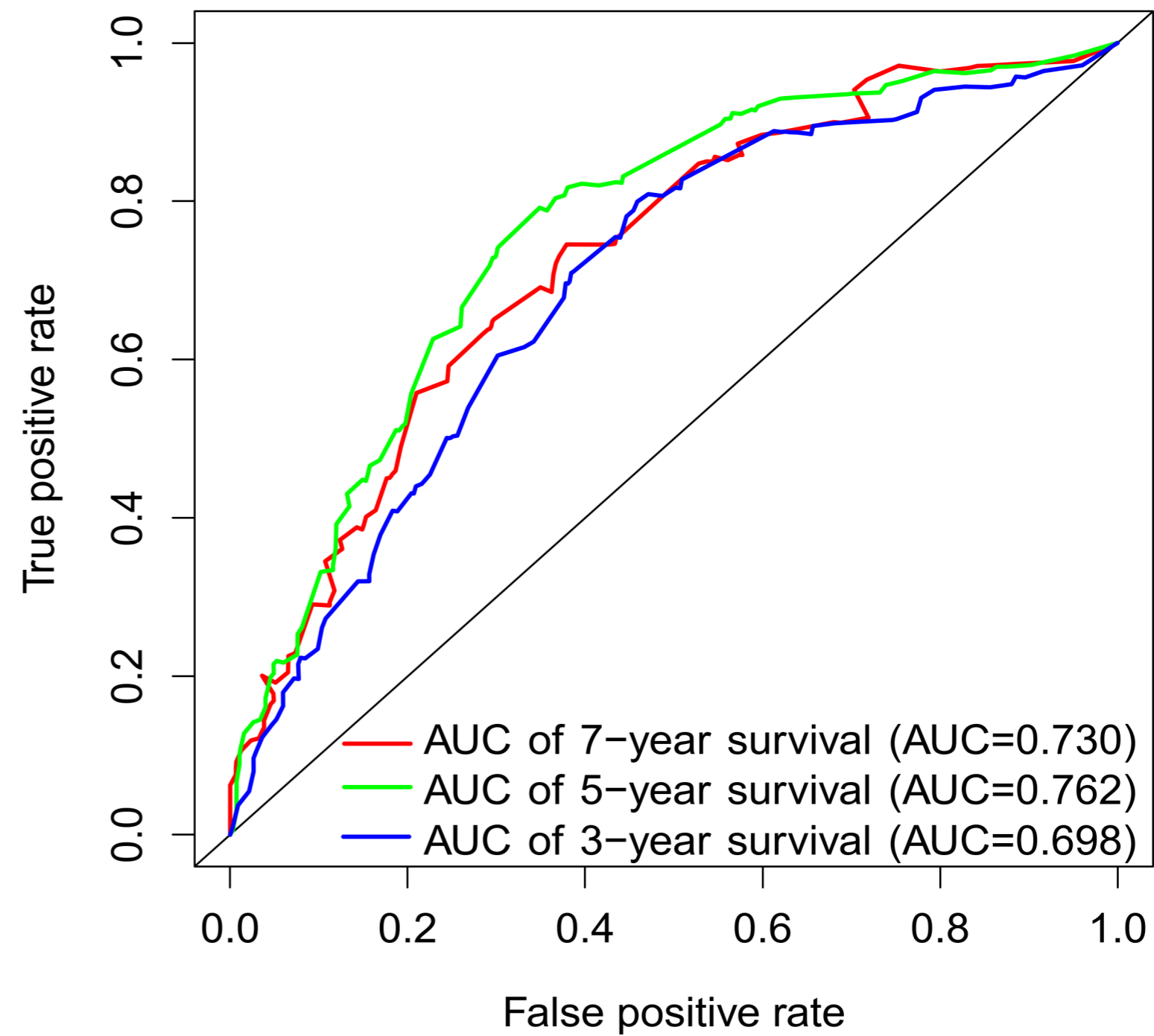

Supplement: Supplementary 5 — Figure S2: prognostic analysis of the five-lncRNA signature in ICGC dataset. (A) Kaplan-Meier survival analysis of the five-lncRNA signature. (B) Time-dependent ROC curves of the five-lncRNA signature. [file 9921466.f5.pdf]

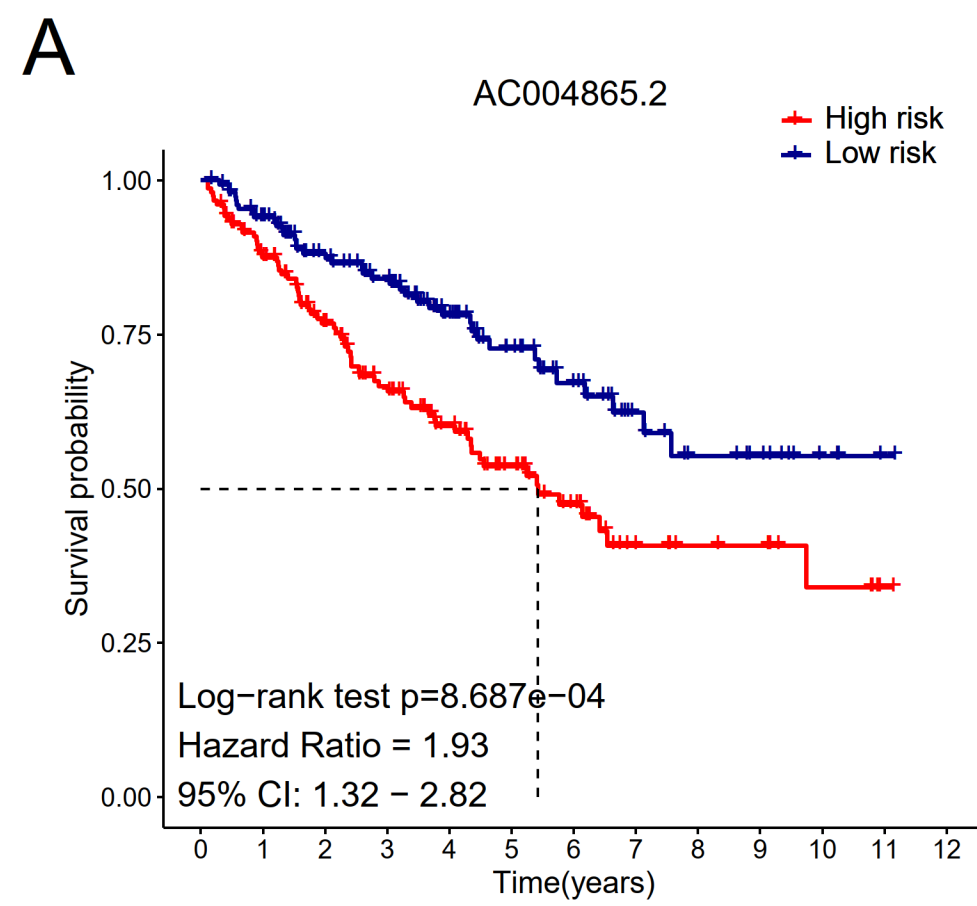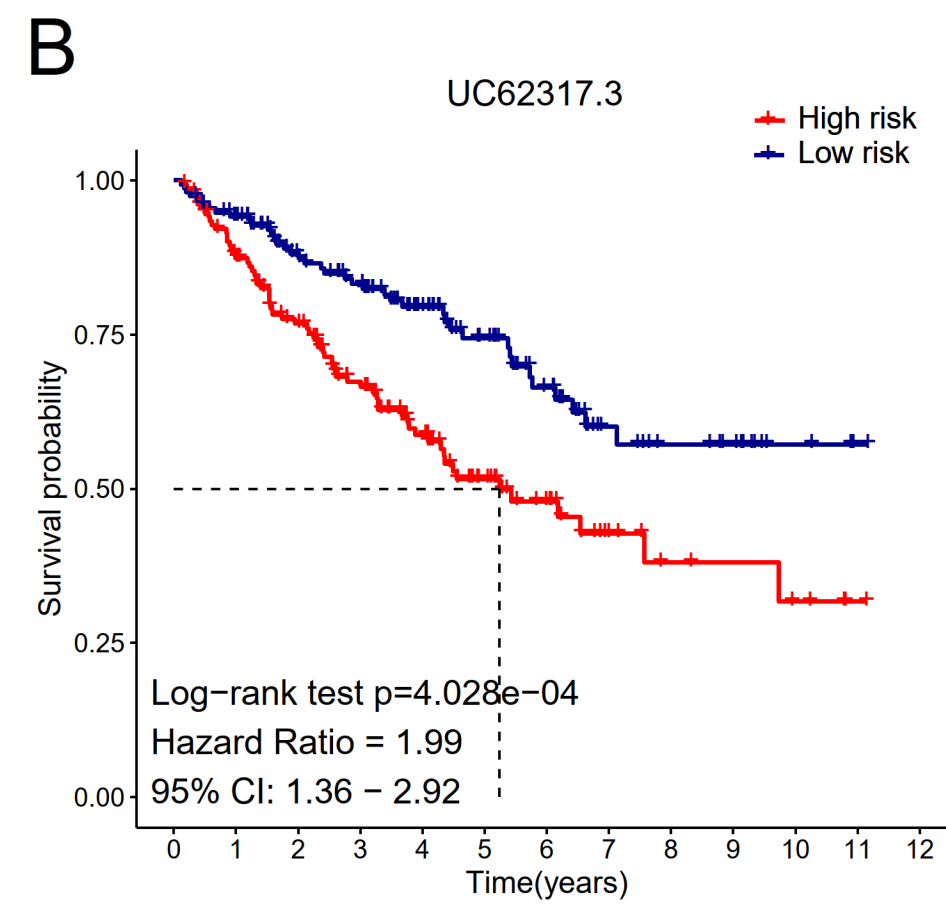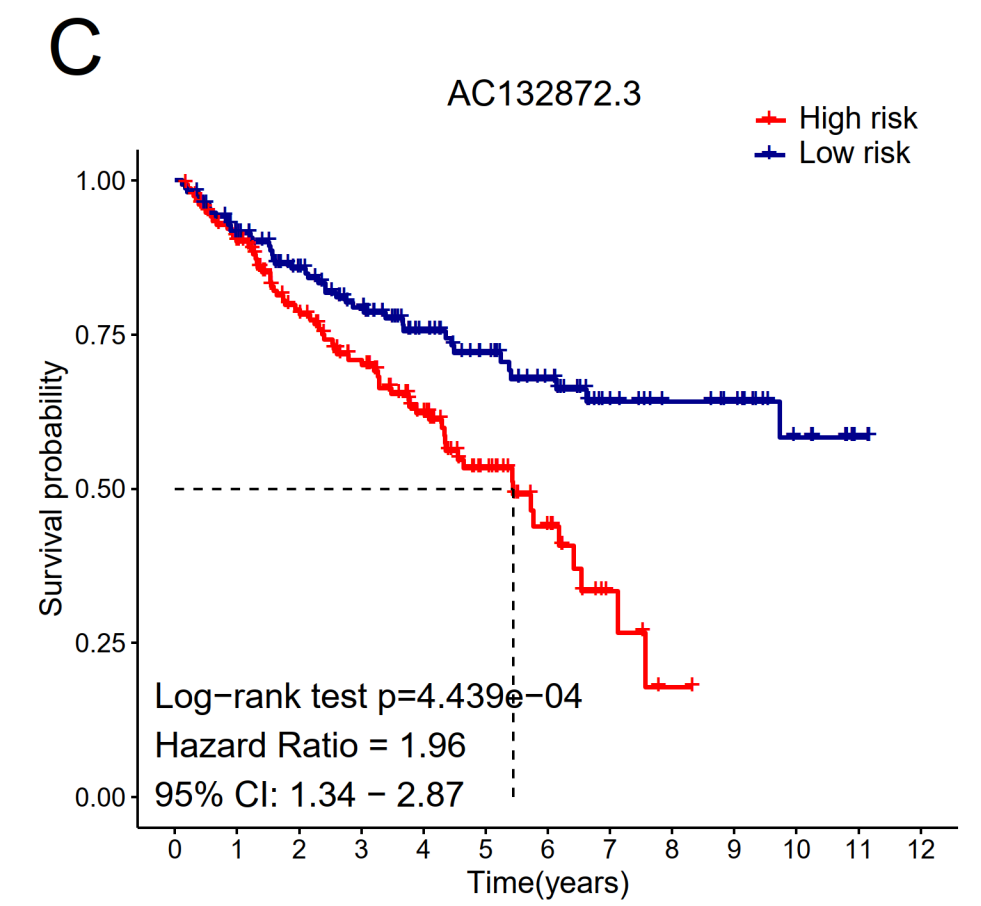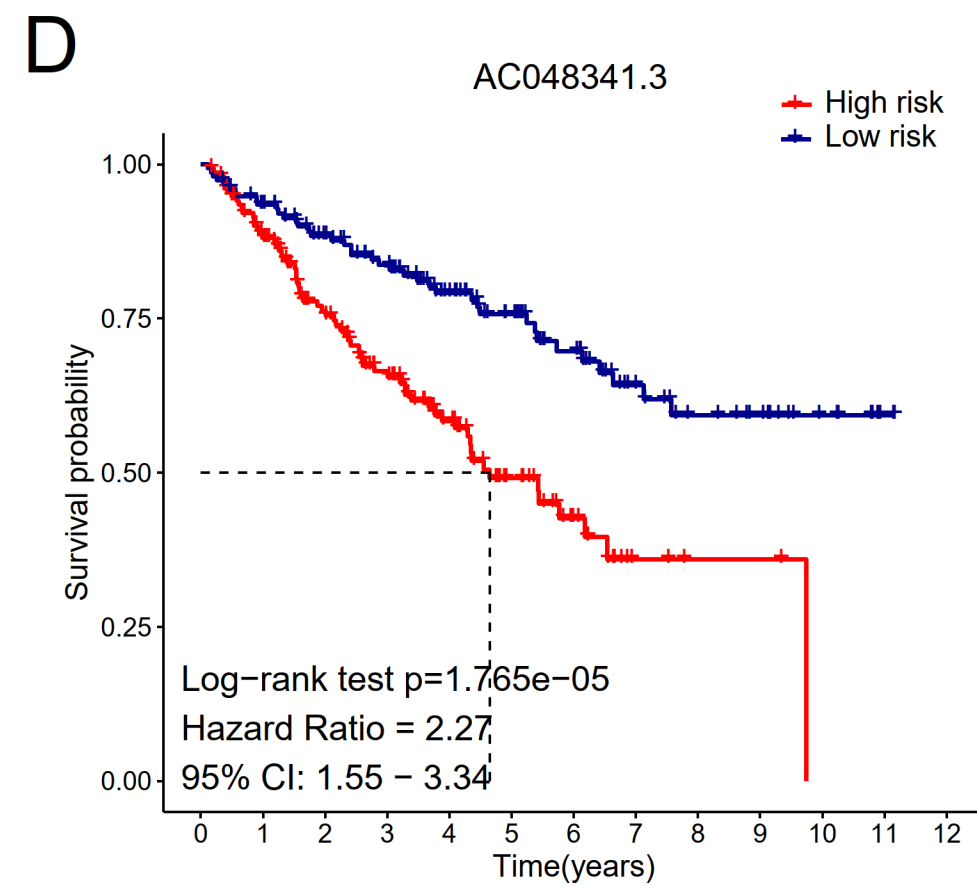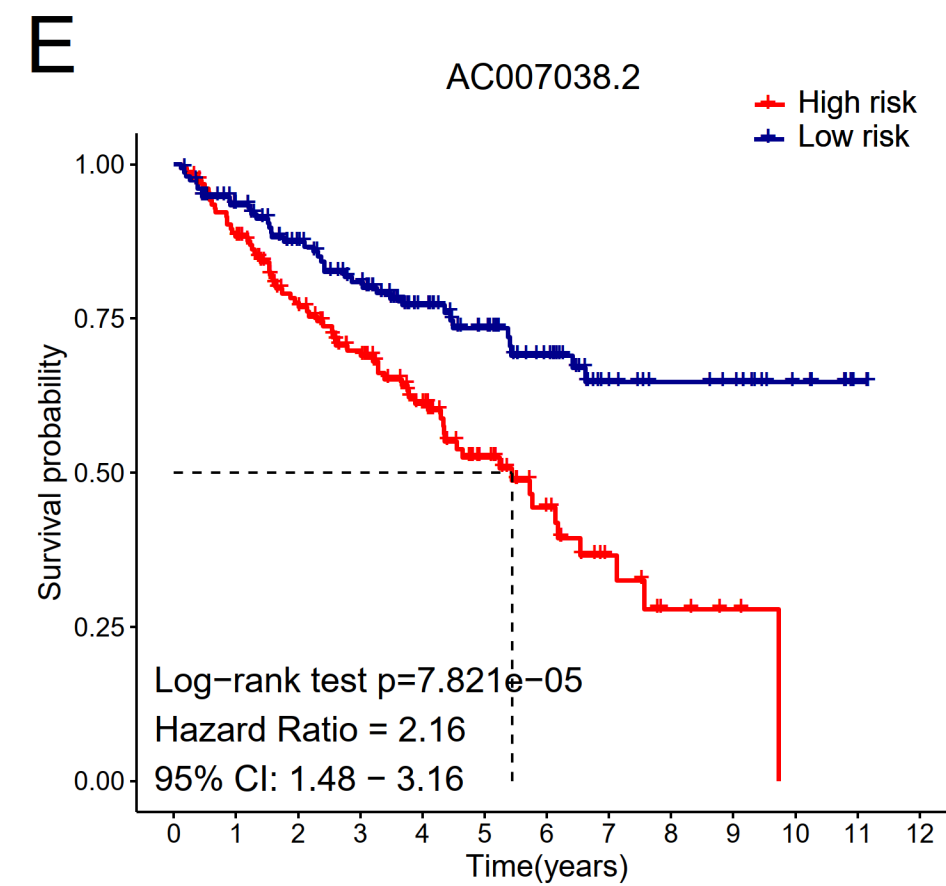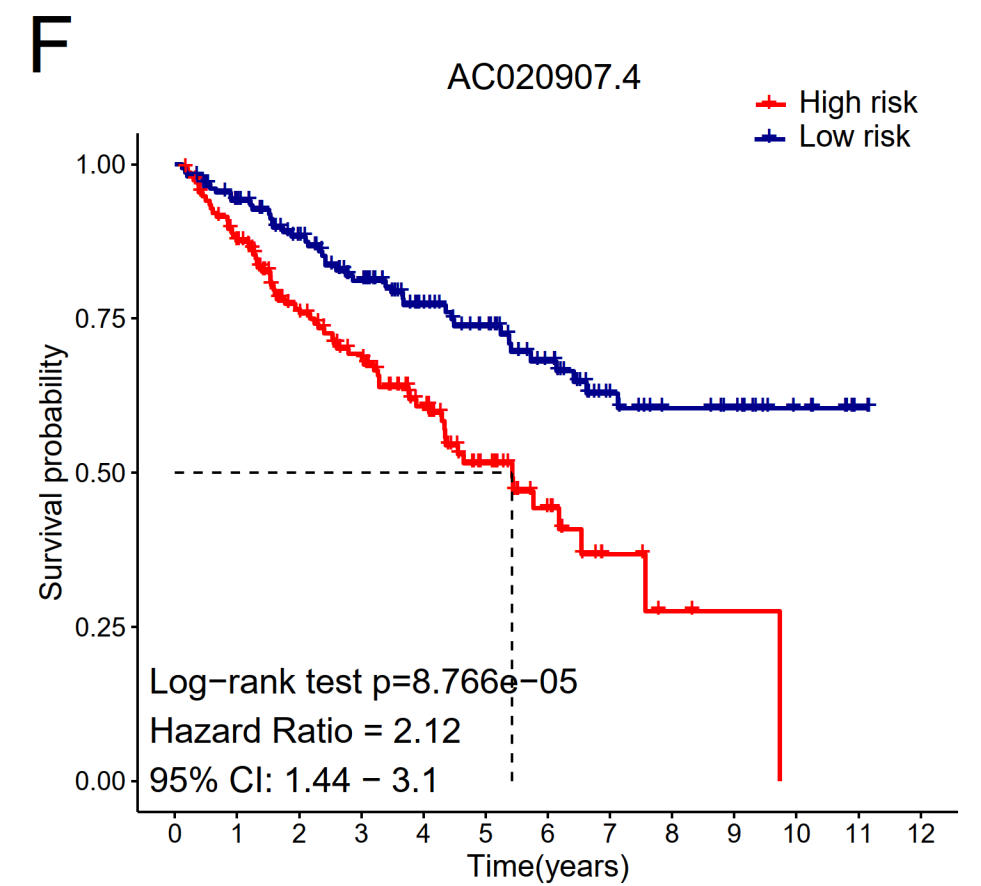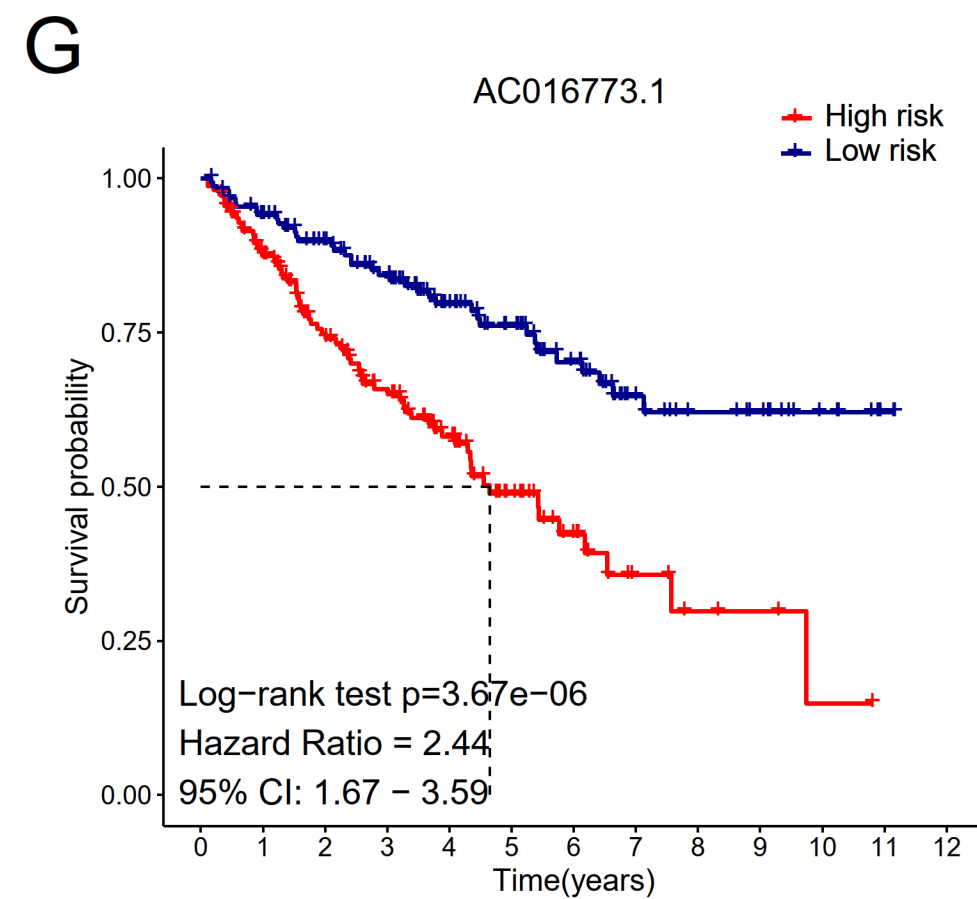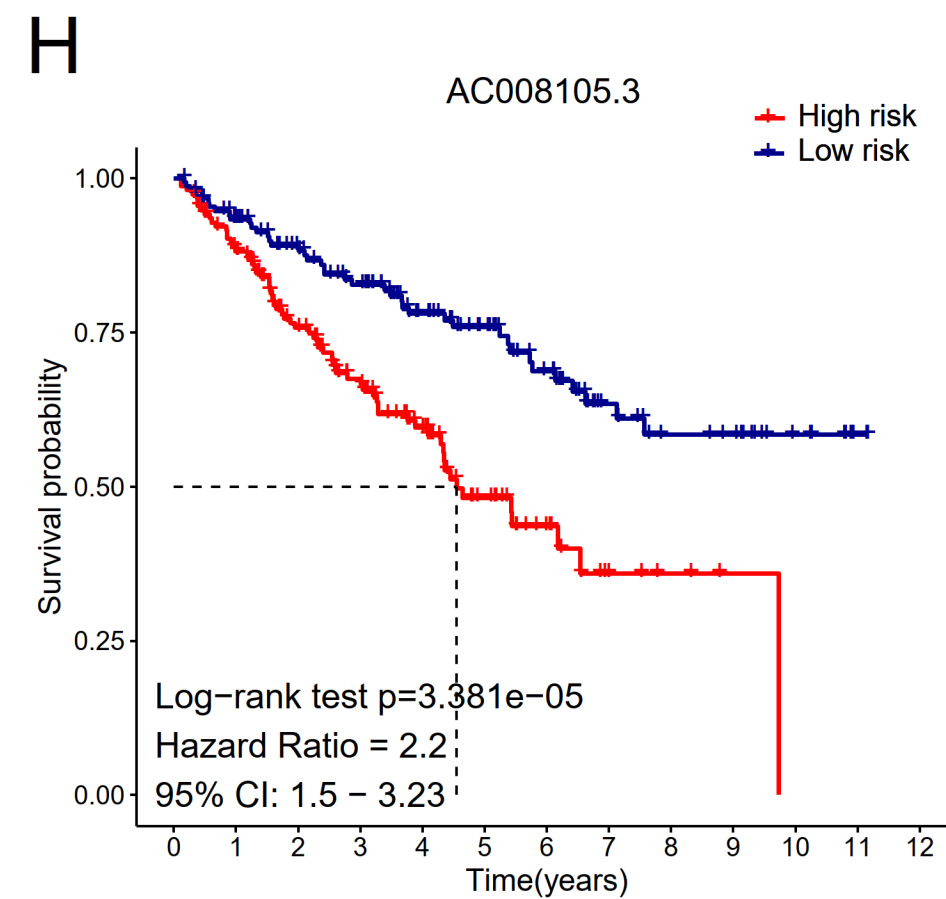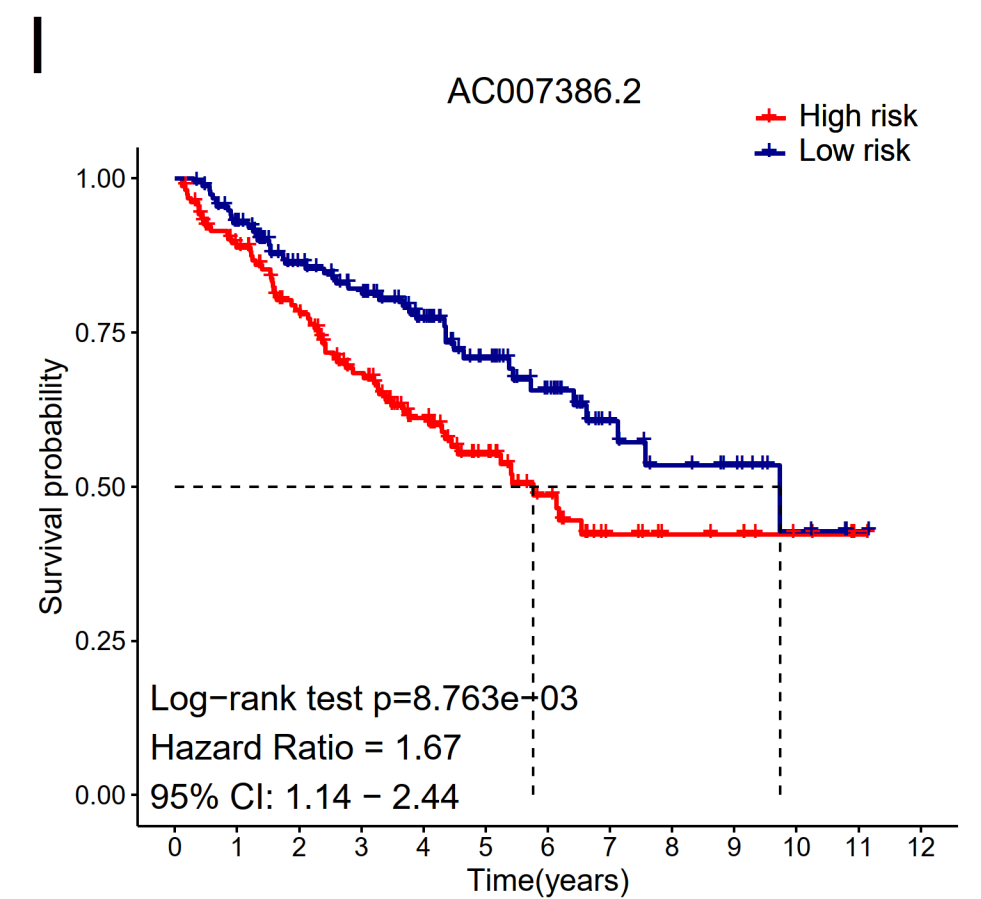

Supplement: Supplementary 6 — Figure S3: Kaplan-Meier survival analysis of other 9 PIDElncRNAs screened after LASSO regression. [file 9921466.f6.pdf]

A

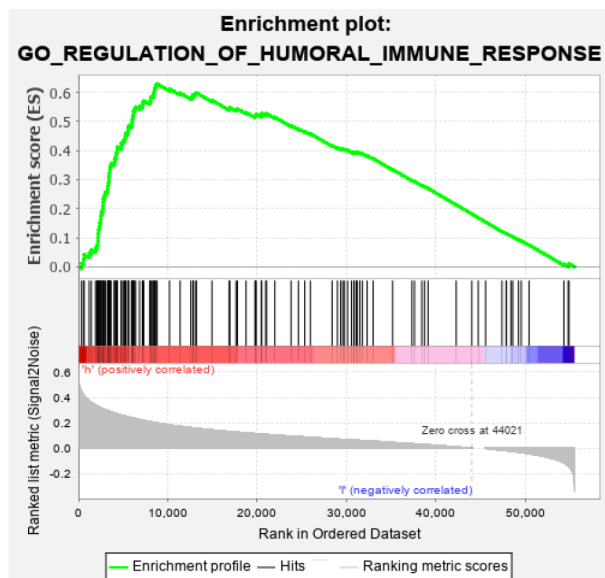

B

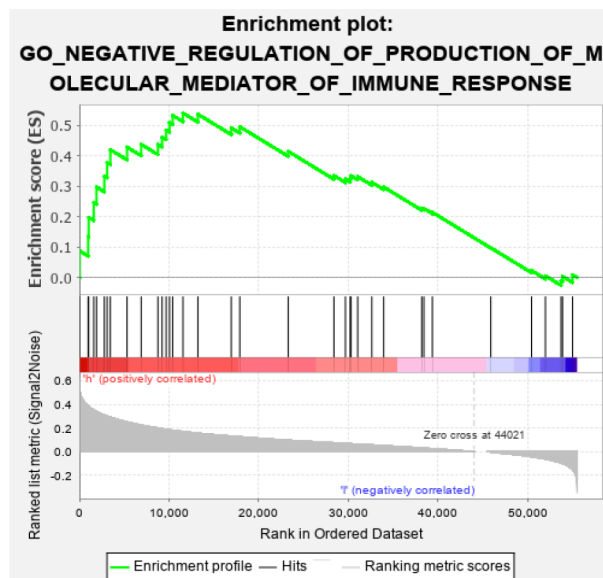

C

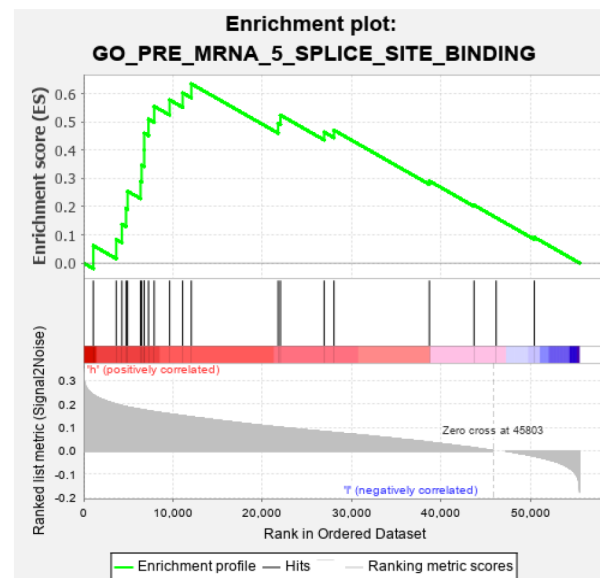

D

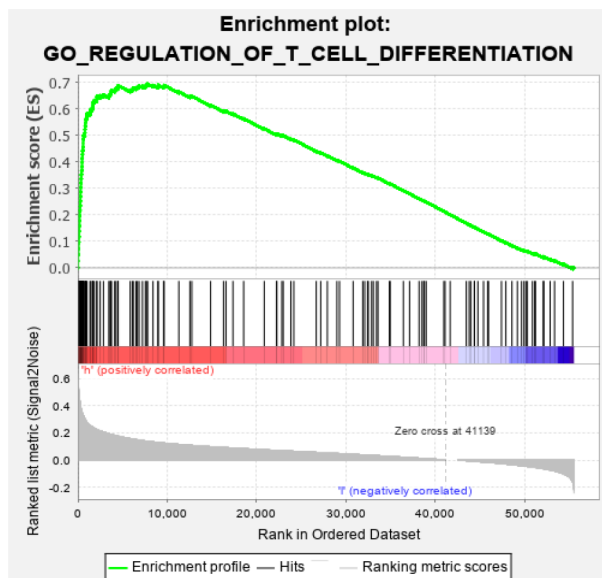

E

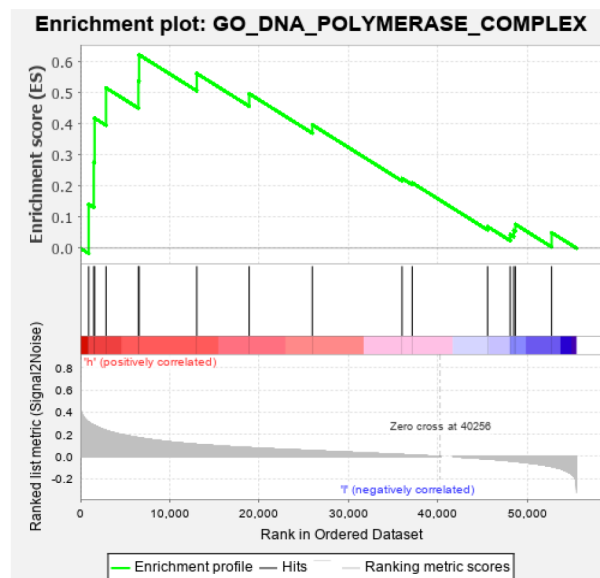

F

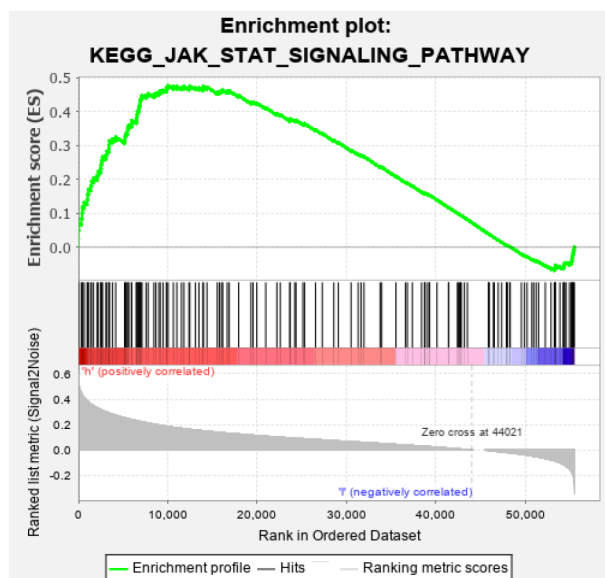

G

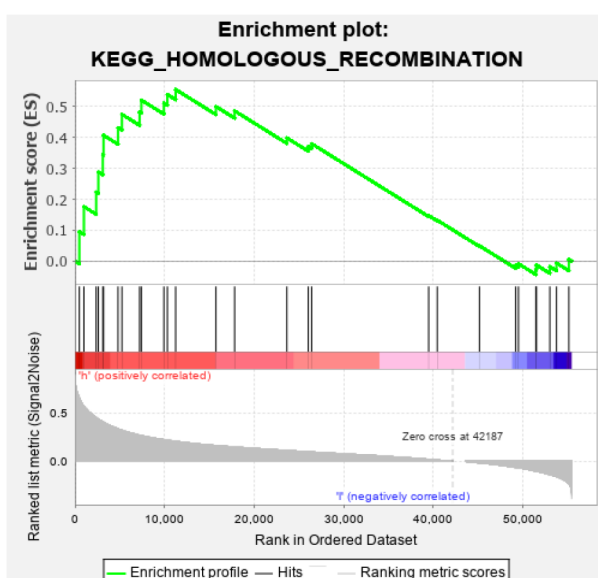

H

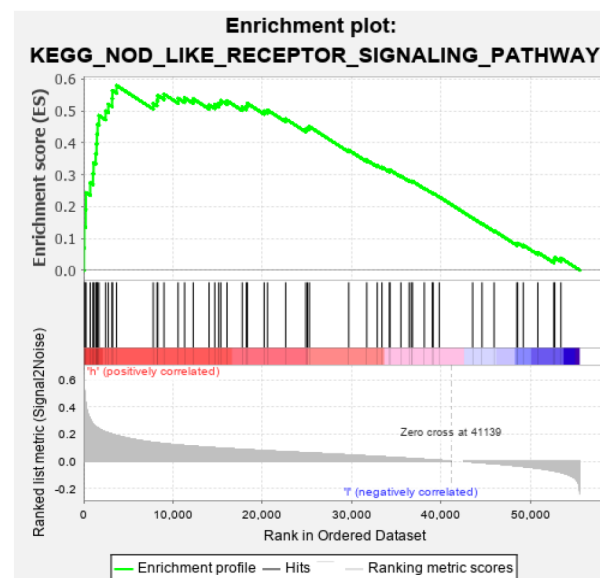

I

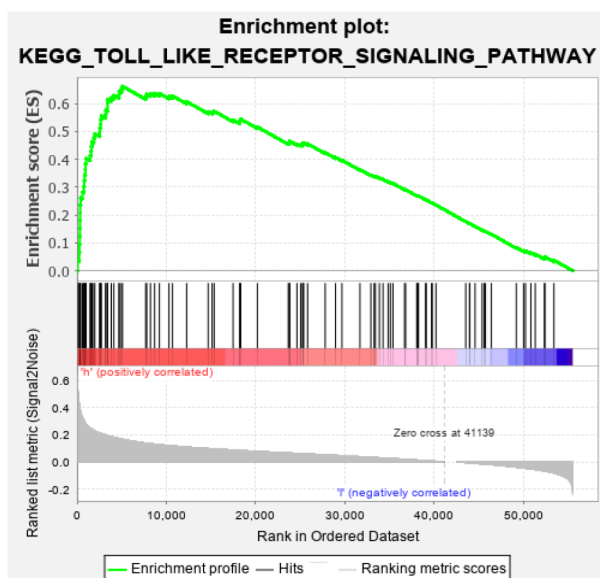

J

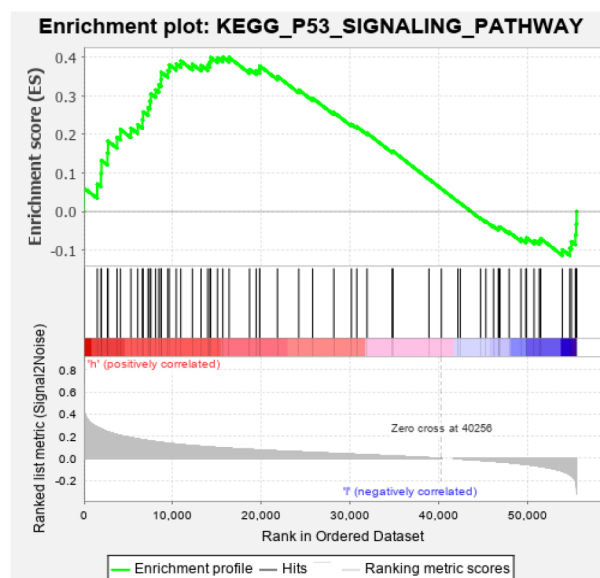

Supplement: Supplementary 7 — Figure S4: GSEA of the five lncRNAs. GO enrichment analysis of AC012236.1 (A), AC078778.1 (B), AC078950.1 (C), AC087318.1 (D), and AC092535.4 (E). KEGG enrichment analysis of AC012236.1 (F), AC078778.1 (G), AC078950.1 (H), AC087318.1 (I), and AC092535.4 (J). [file 9921466.f7.pdf]

A

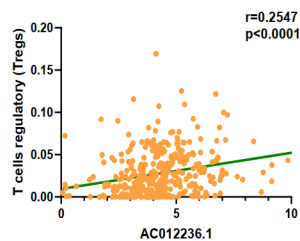

B

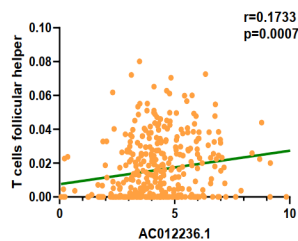

C

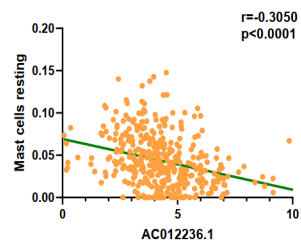

D

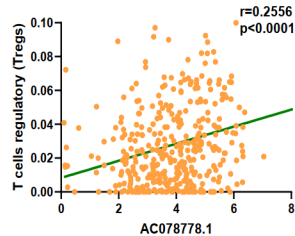

E

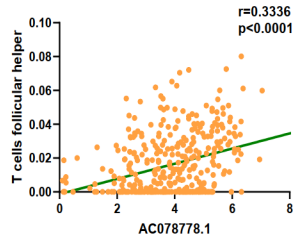

F

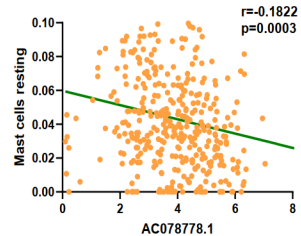

G

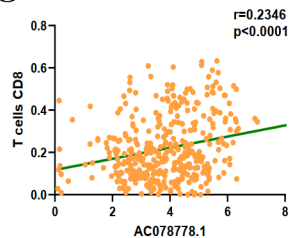

H

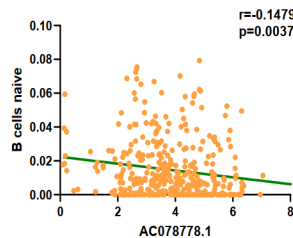

I

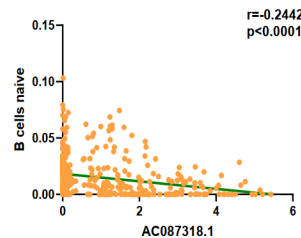

J

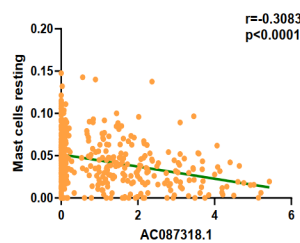

K

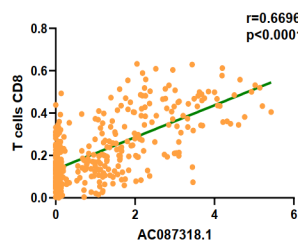

L

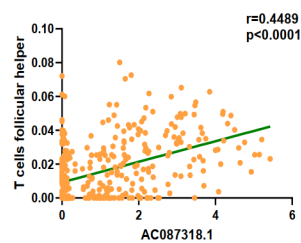

M

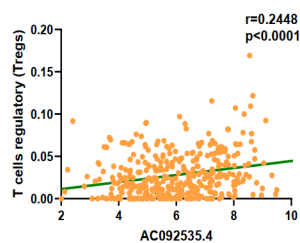

N

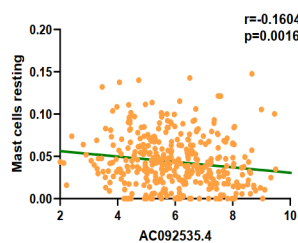

Supplement: Supplementary 8 — Figure S5: Pearson correlation analysis of the expression of five lncRNAs and tumor-infiltrating immune cells. The expression of AC012236.1 was positively correlated with the infiltration of regulatory T cells (A) and follicular helper T cell (B) and negatively correlated with that of resting mast cells (C). The expression of AC078778.1 was positively correlated with the infiltration of regulatory T cells (D), follicular helper T cells (E), and CD8 T cells (G) and negatively correlated with that of resting mast cells (F) and naive B cells (H). The expression of AC087318.1 was negatively correlated with the infiltration of naive B cells (I) and resting mast cells (J) and positively correlated with that of CD8 T cells (K) and follicular helper T cells (L). The expression of AC092535.4 was positively correlated with the infiltration of regulatory T cells (M) and negatively correlated with that of mast cell resting (N). [file 9921466.f8.pdf]
