# Supplementary material for: A Novel Immune-Related lncRNA-Based Model for Survival Prediction in Clear Cell Renal Cell Carcinoma
Source: J Immunol Res. 2021 Jun 28;2021:9921466. doi: 10.1155/2021/9921466 (PMC8339875; doi:10.1155/2021/9921466)
Supplement: Supplementary 9 — Table S4: the coexpression analysis of DETFs and final 5 PIDElncRNAs in TCGA dataset. [file 9921466.f9.docx]

**Table S4: The co-expression analysis of DETFs and final 5 PIDElncRNAs in TCGA dataset**

| **DETFs** | **PIDElncRNAs** | **Cor** | **P-value** | **Regulation** |
| --- | --- | --- | --- | --- |
| BATF | AC012236.1 | 0.501 | 1.47E-35 | positive |
| CEBPA | AC012236.1 | 0.331 | 3.00E-15 | positive |
| CEBPB | AC012236.1 | 0.406 | 8.24E-23 | positive |
| CENPA | AC012236.1 | 0.392 | 3.08E-21 | positive |
| EOMES | AC012236.1 | 0.307 | 2.90E-13 | positive |
| EZH2 | AC012236.1 | 0.382 | 3.35E-20 | positive |
| FOXM1 | AC012236.1 | 0.387 | 1.04E-20 | positive |
| FOXP3 | AC012236.1 | 0.523 | 3.66E-39 | positive |
| HEY1 | AC012236.1 | -0.337 | 8.52E-16 | negative |
| IKZF1 | AC012236.1 | 0.351 | 4.07E-17 | positive |
| IRF4 | AC012236.1 | 0.609 | 6.01E-56 | positive |
| LEF1 | AC012236.1 | 0.371 | 5.07E-19 | positive |
| LMNB1 | AC012236.1 | 0.359 | 7.14E-18 | positive |
| MYBL2 | AC012236.1 | 0.476 | 8.65E-32 | positive |
| NCAPG | AC012236.1 | 0.342 | 3.25E-16 | positive |
| RUNX1 | AC012236.1 | 0.384 | 2.34E-20 | positive |
| STAT4 | AC012236.1 | 0.413 | 1.29E-23 | positive |
| CENPA | AC078778.1 | 0.371 | 5.32E-19 | positive |
| ETS1 | AC078778.1 | -0.339 | 5.41E-16 | negative |
| EZH2 | AC078778.1 | 0.52 | 9.64E-39 | positive |
| FOXM1 | AC078778.1 | 0.301 | 9.01E-13 | positive |
| PBX1 | AC078778.1 | -0.316 | 5.51E-14 | negative |
| POU5F1 | AC078778.1 | 0.415 | 6.60E-24 | positive |
| STAT4 | AC078778.1 | 0.343 | 2.55E-16 | positive |
| ETS1 | AC078950.1 | -0.355 | 1.96E-17 | negative |
| FLI1 | AC078950.1 | -0.309 | 2.06E-13 | negative |
| MYC | AC078950.1 | -0.358 | 9.55E-18 | negative |
| PML | AC078950.1 | -0.388 | 7.57E-21 | negative |
| RARA | AC078950.1 | -0.41 | 3.23E-23 | negative |
| SAP30 | AC078950.1 | -0.472 | 2.58E-31 | negative |
| BATF | AC087318.1 | 0.598 | 1.51E-53 | positive |
| CEBPA | AC087318.1 | 0.354 | 2.59E-17 | positive |
| CIITA | AC087318.1 | 0.578 | 2.38E-49 | positive |
| EOMES | AC087318.1 | 0.774 | 1.07E-108 | positive |
| EZH2 | AC087318.1 | 0.459 | 1.84E-29 | positive |
| FOXP3 | AC087318.1 | 0.424 | 7.14E-25 | positive |
| IKZF1 | AC087318.1 | 0.625 | 8.23E-60 | positive |
| IRF1 | AC087318.1 | 0.608 | 6.83E-56 | positive |
| IRF4 | AC087318.1 | 0.622 | 5.40E-59 | positive |
| LMNB1 | AC087318.1 | 0.417 | 4.27E-24 | positive |
| NCAPG | AC087318.1 | 0.316 | 5.96E-14 | positive |
| PML | AC087318.1 | 0.306 | 3.94E-13 | positive |
| PRDM1 | AC087318.1 | 0.314 | 8.14E-14 | positive |
| STAT4 | AC087318.1 | 0.481 | 1.54E-32 | positive |
| CEBPB | AC092535.4 | 0.324 | 1.26E-14 | positive |
