# Supplementary material for: A Novel Immune-Related lncRNA-Based Model for Survival Prediction in Clear Cell Renal Cell Carcinoma
Source: J Immunol Res. 2021 Jun 28;2021:9921466. doi: 10.1155/2021/9921466 (PMC8339875; doi:10.1155/2021/9921466)
Supplement: Supplementary 10 — Table S5: the coexpression analysis of DETFs and final 5 PIDElncRNAs in ICGC dataset. [file 9921466.f10.docx]

**Table S5: The validation of co-expression relationship between the DETFs and the final 5 PIDElncRNAs in ICGC dataset**

| **DETFs** | **PIDElncRNAs** | **Cor** | **P-value** | **Regulation** |
| --- | --- | --- | --- | --- |
| BATF | AC012236.1 | **0.209** | **0.0272** | positive |
| CEBPA | AC012236.1 | 0.00792 | 0.927 |  |
| CEBPB | AC012236.1 | 0.00212 | 0.984 |  |
| CENPA | AC012236.1 | -0.219 | 0.079 |  |
| EOMES | AC012236.1 | **0.207** | **0.0021** | positive |
| EZH2 | AC012236.1 | **0.246** | **0.0056** | positive |
| FOXM1 | AC012236.1 | **0.297** | **5.21E-7** | positive |
| FOXP3 | AC012236.1 | **0.321** | **0.0002** | positive |
| HEY1 | AC012236.1 | **-0.429** | **4.52E-11** | negative |
| IKZF1 | AC012236.1 | -0.185 | 0.1487 |  |
| IRF4 | AC012236.1 | **0.391** | **0.0001** | positive |
| LEF1 | AC012236.1 | **0.284** | **0.0051** | positive |
| LMNB1 | AC012236.1 | **0.347** | **0.0012** | positive |
| MYBL2 | AC012236.1 | 0.0212 | 0.942 |  |
| NCAPG | AC012236.1 | 0.0761 | 0.543 |  |
| RUNX1 | AC012236.1 | **0.225** | **0.0130** | positive |
| STAT4 | AC012236.1 | **0.325** | **0.0002** | positive |
| CENPA | AC078778.1 | -0.071 | 0.4926 |  |
| ETS1 | AC078778.1 | **-0.275** | **0.0154** | negative |
| EZH2 | AC078778.1 | **0.332** | **0.0013** | positive |
| FOXM1 | AC078778.1 | -0.134 | 0.2059 |  |
| PBX1 | AC078778.1 | **-0.245** | **0.0045** | negative |
| POU5F1 | AC078778.1 | **0.446** | **7.60E-22** | positive |
| STAT4 | AC078778.1 | **0.318** | **0.0022** | positive |
| ETS1 | AC078950.1 | -0.071 | 0.4914 |  |
| FLI1 | AC078950.1 | **-0.269** | **0.0012** | negative |
| MYC | AC078950.1 | -0.0283 | 0.7902 |  |
| PML | AC078950.1 | **-0.169** | **0.0494** | negative |
| RARA | AC078950.1 | **-0.313** | **8.12E-18** | negative |
| SAP30 | AC078950.1 | -0.072 | 0.6759 |  |
| BATF | AC087318.1 | **0.474** | **2.71E-34** | positive |
| CEBPA | AC087318.1 | **0.183** | **0.0031** | positive |
| CIITA | AC087318.1 | **0.276** | **0.0066** | positive |
| EOMES | AC087318.1 | **0.534** | **6.14E-58** | positive |
| EZH2 | AC087318.1 | **0.259** | **0.0131** | positive |
| FOXP3 | AC087318.1 | 0.036 | 0.7356 |  |
| IKZF1 | AC087318.1 | **0.257** | **0.0139** | positive |
| IRF1 | AC087318.1 | **0.297** | **0.0042** | positive |
| IRF4 | AC087318.1 | **0.452** | **7.53E-39** | positive |
| LMNB1 | AC087318.1 | **0.273** | **0.0013** | positive |
| NCAPG | AC087318.1 | **0.299** | **0.0004** | positive |
| PML | AC087318.1 | **0.231** | **0.0069** | positive |
| PRDM1 | AC087318.1 | **0.192** | **0.0251** | positive |
| STAT4 | AC087318.1 | **0.242** | **0.0210** | positive |
| CEBPB | AC092535.4 | **0.191** | **0.0248** | positive |
