# Supplementary material for: A Novel Immune-Related lncRNA-Based Model for Survival Prediction in Clear Cell Renal Cell Carcinoma
Source: J Immunol Res. 2021 Jun 28;2021:9921466. doi: 10.1155/2021/9921466 (PMC8339875; doi:10.1155/2021/9921466)
Supplement: Supplementary 11 — Table S6: the coexpression analysis between the 5 PIDElncRNAs and immune-related mRNA. [file 9921466.f11.docx]

**Table S6: The co-expression analysis between the 5 PIDElncRNAs and immune-related mRNA**

| **LncRNA** | **Immune-related mRNA** | **Cor** | **P-value** | **Regulation** |
| --- | --- | --- | --- | --- |
| AC012236.1 | TNFRSF17 | 0.712979 | 7.86E-85 | positive |
| AC012236.1 | MZB1 | 0.765461 | 6.86E-105 | positive |
| AC012236.1 | POU2AF1 | 0.745442 | 1.17E-96 | positive |
| AC012236.1 | IGLL5 | 0.704267 | 6.20E-82 | positive |
| AC012236.1 | CD79A | 0.706764 | 9.40E-83 | positive |
| AC078778.1 | GNRH1 | 0.716094 | 6.80E-86 | positive |
| AC078778.1 | ATAD3B | 0.72067 | 1.76E-87 | positive |
| AC078950.1 | STAP1 | 0.714423 | 2.54E-85 | positive |
| AC087318.1 | CRTAM | 0.756002 | 6.70E-101 | positive |
| AC087318.1 | SLA2 | 0.712847 | 8.71E-85 | positive |
| AC087318.1 | PDCD1 | 0.739298 | 2.77E-94 | positive |
| AC087318.1 | CD8A | 0.725265 | 4.17E-89 | positive |
| AC087318.1 | GBP5 | 0.711641 | 2.22E-84 | positive |
| AC087318.1 | CD8B | 0.717369 | 2.47E-86 | positive |
| AC087318.1 | IKZF3 | 0.72639 | 1.65E-89 | positive |
| AC087318.1 | IFNG | 0.748973 | 4.72E-98 | positive |
| AC087318.1 | EOMES | 0.774087 | 1.07E-108 | positive |
| AC087318.1 | CD200R1 | 0.732543 | 9.45E-92 | positive |
| AC087318.1 | FCRL3 | 0.709083 | 1.60E-83 | positive |
| AC087318.1 | SIRPG | 0.740324 | 1.12E-94 | positive |
| AC087318.1 | LAG3 | 0.730743 | 4.34E-91 | positive |
| AC087318.1 | ICOS | 0.740774 | 7.56E-95 | positive |
| AC087318.1 | SLAMF6 | 0.704172 | 6.66E-82 | positive |
| AC087318.1 | TIGIT | 0.738272 | 6.80E-94 | positive |
| AC087318.1 | SH2D1A | 0.705072 | 3.38E-82 | positive |
| AC087318.1 | UBASH3A | 0.723859 | 1.32E-88 | positive |
| AC087318.1 | CD3G | 0.712967 | 7.93E-85 | positive |
| AC087318.1 | PYHIN1 | 0.746693 | 3.78E-97 | positive |
| AC087318.1 | TOX | 0.742051 | 2.44E-95 | positive |
| AC087318.1 | TRAT1 | 0.725552 | 3.29E-89 | positive |
| AC087318.1 | GFI1 | 0.746002 | 7.07E-97 | positive |
| AC092535.4 | SPON2 | 0.858991 | 2.99E-158 | positive |
